# Marine N-3 Fatty Acids Mitigate Hyperglycemia in Prediabetes by Improving Muscular Glucose Transporter 4 Translocation and Glucose Homeostasis

**DOI:** 10.34133/research.0683

**Published:** 2025-04-29

**Authors:** Haoyu Li, Pan Zhuang, Xiaohui Liu, Yin Li, Yang Ao, Yimei Tian, Wei Jia, Yu Zhang, Jingjing Jiao

**Affiliations:** ^1^ Department of Endocrinology, The Second Affiliated Hospital, Zhejiang University School of Medicine, Hangzhou 310009, Zhejiang, China.; ^2^ National Engineering Laboratory of Intelligent Food Technology and Equipment, Zhejiang Key Laboratory of Agri-food Reources and High-value Utilization, College of Biosystems Engineering and Food Science, Zhejiang University, Hangzhou 310058, Zhejiang, China.; ^3^ Department of Gastroenterology, The First Affiliated Hospital, Zhejiang University School of Medicine, Hangzhou 310003, Zhejiang, China.; ^4^ Department of Nutrition, School of Public Health, Zhejiang University School of Medicine, Hangzhou 310058, Zhejiang, China.

## Abstract

Docosahexaenoic acid (DHA) and eicosapentaenoic acid (EPA) have been proposed to benefit cardiometabolic health. However, the relationship between the intake of DHA and EPA and type 2 diabetes (T2D) risk remains equivocal, and the effects of DHA and EPA on skeletal muscle, the primary organ for glucose metabolism, merit further investigation. Here, we show that habitual fish oil supplementation was associated with a 9% lower T2D risk and significantly interacted with variants at GLUT4 in a prospective cohort of 48,358 people with prediabetes. Muscular metabolome analysis in the animal study revealed that DHA and EPA altered branched-chain amino acids, creatine, and glucose oxidation-related metabolites, concurrently with elevated muscular glycogen synthase and pyruvate dehydrogenase contents that promoted glucose disposal. Further myotube investigation revealed that DHA and EPA promoted muscular GLUT4 translocation by elevating Rab GTPases and target-SNARE expression. Together, DHA and EPA supplementation provides a promising approach for T2D prevention through targeting muscular glucose homeostasis, including enhancing GLUT4 translocation, glycogen synthesis, and aerobic glycolysis.

## Introduction

Type 2 diabetes (T2D) characterized by hyperglycemia has reached pandemic levels and become one of the leading causes of morbidity and mortality worldwide [1]. The International Diabetes Federation (IDF) estimated that 1 in 10 adults aged 20 to 79 years (537 million adults) are living with diabetes globally in 2021, and T2D accounts for over 90% of all diabetes [2]. Moreover, a recent study based on data of the National Health and Nutrition Examination Survey (NHANES) revealed that 38.6% of US adults are estimated to have prediabetes in 2020 [3], which suggested a higher risk of developing T2D and diabetes-related complications. Thus, there is an urgent need for effective measures on improving glucose metabolism, which is the key step to preventing T2D development in prediabetes.

High-fat and high-energy dietary patterns, genetic risk, and a sedentary lifestyle were major factors contributing to the high risk of T2D. Particularly, diet, including dietary fats, has been evidenced as a critical contributor to the development of T2D [4]. Among dietary fats, marine n-3 polyunsaturated fatty acids (PUFAs), including eicosapentaenoic acid (EPA) and docosahexaenoic acid (DHA), have been widely reported to alleviate inflammation, improve dyslipidemia, and mitigate hyperglycemia in animals [5,6]. Early human observational studies concluded that the intake of n-3 PUFAs was associated with lower risk of developing T2D in Asia but was related to unaltered or higher risk in North American/European populations [7,8]. Recently, a meta-analysis of randomized controlled trials (RCTs) has suggested no protective effect of fish oil supplements on glucose control and diabetes onset [9]. The above discrepancy between clinical trials may be largely ascribed to restricted follow-up duration and limited newly diagnosed T2D cases, which might not provide sufficient statistical power to detect significant effects. Moreover, the designated patients, ideal and controlled circumstances, race, and region also limit the generalization of findings from these trials to larger and more inclusive populations [10]. Importantly, no clinical studies have reported the effect of long-term n-3 PUFA supplementation on T2D risk in people with prediabetes who are at a high risk for T2D development. Meanwhile, the gene–nutrient interaction between individual genetic risk and n-3 PUFA intake also drives the heterogeneity among different studies [11]. Up to now, many genome-wide association studies have identified more than 500 genomic loci related to T2D, while these single-nucleotide polymorphisms (SNPs) can be further aggregated into genetic risk scores (GRSs) for assessing the genetic risk of T2D [12,13]. Unfortunately, evidence is still scarce whether the genetic predisposition modifies the association of n-3 PUFA intake with T2D risk.

The underlying role of marine n-3 PUFAs in glycemic control via skeletal muscle is still poorly understood, although DHA and EPA could promote insulin secretion [14,15], suppress inflammation status, and enhance the production of adipokines [16,17]. Skeletal muscle as the determinant principal tissue is responsible for approximately 80% of whole-body glucose uptake and disposal under insulin stimulation, wherein glucose is consumed through glycogen synthesis or glycolysis [18]. Importantly, a diet supplemented with fish oil promoted skeletal muscle growth [19,20] and prevented obesity-induced muscle wasting [21], which suggested that n-3 PUFAs have the potential to mitigate hyperglycemia by regulating the metabolic profile in skeletal muscle.

In the insulin-stimulated condition, glucose transporter 4 (GLUT4)-containing vesicles are transferred from perinuclear storage sites to the fiber surface, and further fuse with the plasma membrane in skeletal muscle, which elicits an accumulation of GLUT4 on the plasma membrane and further mediates glucose transport [22]. In this process, Rab guanosine triphosphatases (GTPases) play a determinant role in promoting intracellular traffic of GLUT4 vesicles, which are activated via the pathway of insulin–AKT–Rab GTPase-activating proteins [23]. Subsequently, when GLUT4 vesicles arrive at the cell membrane, the tether and fusion between vesicles and plasma membranes will be elicited by soluble *N*-ethylmaleimide-sensitive factor-attachment protein receptors (SNAREs). One vesicular SNARE (v-SNARE), VAMP2, promotes membrane fusion, while 2 targeted-membrane SNAREs (t-SNAREs) are essential for the tethering process [24]. Notably, skeletal muscle insulin resistance (IR) often occurs before the onset of T2D, in which muscular glucose uptake is depressed due to the lack of GLUT4 on plasma membranes [25]. Therefore, promoting GLUT4 translocation in skeletal muscle is critical for T2D prevention and treatment. However, whether n-3 PUFAs benefit this process remains obscure.

This work aimed to investigate the relationship between fish oil use and the development of T2D, focusing on the underlying mechanism of the hypoglycemic effect regarding the modulation of glucose homeostasis in skeletal muscle. Herein, we hypothesized that long-term habitual fish oil supplementation will be inversely associated with the risk of T2D development among prediabetes participants, which is not modified by genetic predisposition. To further assess the potential causal role of n-3 PUFAs on hyperglycemia mitigation, we next questioned whether DHA and EPA promote GLUT4 translocation, glucose oxidization, and glycogen synthesis in myotubes and skeletal muscle of diabetic mice, which may alleviate muscular IR and restore glucose homeostasis.

## Results

### Habitual fish oil supplementation is associated with lower risk of incident T2D among people with prediabetes

**Fig. 1.**
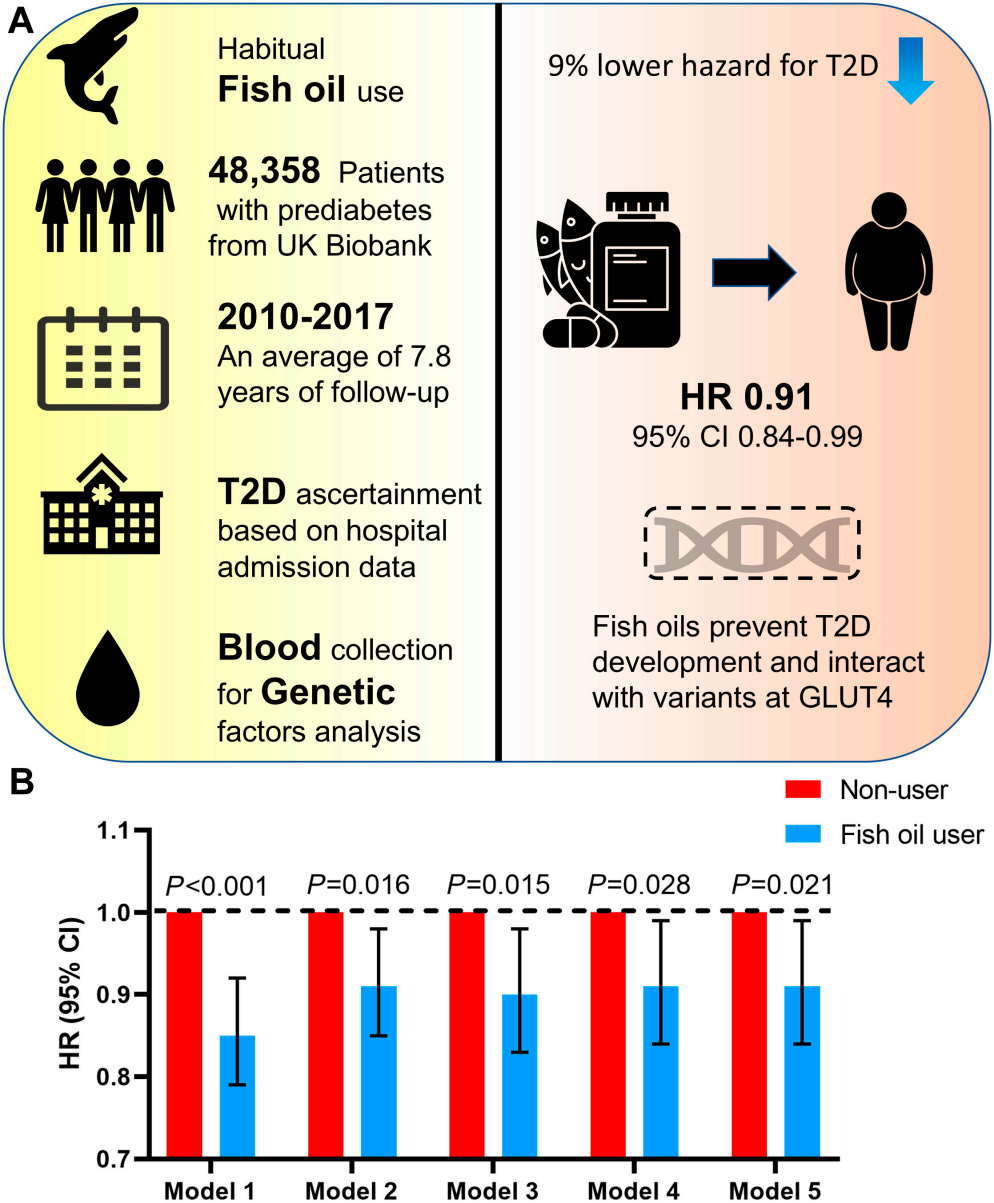
Fish oil supplements prevent T2D development in people with prediabetes. (A) Flow chart of prospective, longitudinal cohort study. In total, 48,358 people with prediabetes from UK Biobank were selected for an average of 7.8 years of follow-up, of which the cumulative hospital inpatient records were provided to identify incident T2D cases. The interactions between fish oil use and genetic factors were subsequently analyzed. (B) HR (95% CI) of T2D according to fish oil use in the UK Biobank in people with prediabetes. Model 1: Results were adjusted for age and sex. Model 2: model 1 + race (white or not), centers (22 centers), BMI (<18.5, 18.5 to 25.0, 25.0 to 30.0, >30.0 kg/m^2^, or missing), education (college or university degree, vocational qualifications, optional national exams at ages 17 to 18 years, national exams at age 16 years, others, or missing), Townsend deprivation index (quintiles), household income (<£18,000, £18,000 to £30,999, £31,000 to £51,999, £52,000 to £100,000, >£100,000, or missing), smoking (never, former, current, or missing), alcohol consumption (<1, 1 or 2, 3 or 4, ≥5 times/week, or missing), physical activity (<150 or ≥150 min/week), history of hypertension (yes or no), history of high cholesterol (yes or no), family history of diabetes (yes or no). Model 3: model 2 + vitamin supplement use (yes or no), mineral supplement use (yes or no), glucosamine use (yes or no), aspirin use (yes or no). Model 4: model 3 + oily fish (<1, 1, or ≥2 times/week), vegetables (<1.0, 1.0 to 2.9, or ≥3.0 servings/day), fruits (<2.0, 2.0 to 3.9, or ≥4.0 servings/day). Model 5: model 3 + healthy diet score. HRs are presented with 95% CI.

To explore the association between fish oil supplementation and T2D development, 48,358 people with prediabetes from UK Biobank were selected and eligible for follow-up (Fig. 1A). Among them, 16,180 (33.5%) patients reported habitually taking fish oil supplements. The baseline characteristics of these patients are shown in Table. Compared with non-users, fish oil users were generally older, more often female, more physically active, and not current smokers and had a healthier diet. They also had lower body mass index (BMI), household income, and Townsend deprivation index (TDI), and were more likely to have prevalent hypertension and hypercholesteremia, and use aspirin, vitamins, minerals, and glucosamine. In addition, fish oil users also tended to consume oily fish, non-oily fish, vegetables, fruits, and whole grains more frequently, whereas they consumed processed meat, refined grains, cheese, and sugar-sweetened beverages less frequently.

**Table.** Basic characteristics of people with prediabetes by the use of fish oil supplement in the UK Biobank cohort. Values are means ± SD or percentages unless stated otherwise.

Characteristics	Overall (*n* = 48,358)	Fish oil non-users (*n* = 32,178)	Fish oil users (*n* = 16,180)
Male/*n* (%)	21,287 (44.0)	14,795 (46.0)	6,492 (40.1)
Age (year)	59.0 ± 7.1	58.2 ± 7.4	60.7 ± 6.3
Race, *n* (%)			
White	43,196 (89.3)	28,528 (88.7)	14,668 (90.7)
Nonwhite/mixed	4,956 (10.3)	3,506 (10.9)	1,450 (9.0)
BMI (kg/m^2^)	28.9 ± 5.3	29.1 ± 5.4	28.5 ± 5.0
Household income (£)			
<18,000 ^a^	11,699 (24.2)	7,724 (24.0)	3,975 (24.6)
18,000–30,999	11,446 (23.7)	7,382 (22.9)	4,064 (25.1)
31,000–51,999	9,652 (20.0)	6,540 (20.3)	3,112 (19.2)
52,000–100,000	5,826 (12.1)	4,113 (12.8)	1,713 (10.6)
>100,000	1,375 (2.8)	1,009 (3.1)	366 (2.3)
Education			
College or university degree	12,970 (26.8)	8,734 (27.1)	4,236 (26.2)
Vocational qualifications	6,449 (13.3)	4,170 (13.0)	2,279 (14.1)
Optional national exams at ages 17–18 years	4,748 (9.8)	3,133 (9.7)	1,615 (10.0)
National exams at age 16 years	12,596 (26.1)	8,420 (26.2)	4,176 (25.8)
Others	10,919 (22.6)	7,268 (22.6)	3,651 (22.6)
Townsend deprivation index	−1.0 ± 3.2	−0.9 ± 3.3	−1.3 ± 3.1
Smoking status, *n* (%)			
Never	24,444 (50.6)	16,199 (50.3)	8,245 (51.0)
Previous	16,154 (33.4)	10,248 (31.9)	5,906 (36.5)
Current	7,538 (15.6)	5,582 (17.4)	1,956 (12.1)
Alcohol consumption, *n* (%)			
<1 times/week	18,559 (38.4)	12,726 (39.6)	5,833 (36.1)
1 or 2 times/week	12,144 (25.1)	8,008 (24.9)	4,136 (25.6)
3 or 4 times/week	9,236 (19.1)	5,941 (18.5)	3,295 (20.4)
≥5 times/week	8,354 (17.3)	5,457 (17.0)	2,897 (17.9)
Physical activity (MET-min/week)			
<150	8,211 (17.0)	5,945 (18.5)	2,266 (14.0)
≥150	29,388 (60.8)	18,884 (58.7)	10,504 (64.9)
History of hypertension, *n* (%)	32,087 (66.4)	21,123 (65.6)	10,964 (67.8)
History of high cholesterol, *n* (%)	10,851 (22.4)	6,873 (21.4)	3,978 (24.6)
Family history of CVD, *n* (%)	28,858 (59.7)	19,011 (59.1)	9,847 (60.9)
Family history of diabetes, *n* (%)	13,292 (27.5)	8,935 (27.8)	4,357 (26.9)
Aspirin use, *n* (%)	6,169 (12.8)	3,718 (11.6)	2,451 (15.2)
Vitamin supplementation, *n* (%)	15,206 (31.4)	6,282 (19.5)	8,924 (55.2)
Mineral supplementation, *n* (%)	6,000 (12.4)	2,671 (8.3)	3,329 (20.6)
Glucosamine use (%)	10,043 (20.8)	3,652 (11.4)	6,391 (39.5)
Lipid-lowering drug, *n* (%)	10,312 (21.3)	6,566 (20.4)	3,746 (23.2)
Dietary consumption			
Oily fish (times/week)			
<1	21,310 (44.1)	15,501 (48.2)	5,809 (35.9)
1	17,970 (37.2)	11,329 (35.2)	6,641 (41.0)
≥2	8,696 (18.0)	5,044 (15.7)	3,652 (22.6)
Non-oily fish (times/week)			
<1	16,312 (33.7)	11,651 (36.2)	4,661 (28.8)
1	23,909 (49.4)	15,425 (47.9)	8,484 (52.4)
≥2	7,779 (16.1)	4,832 (15.0)	2,947 (18.2)
Poultry (times/week)			
<2	25,864 (53.5)	17,272 (53.7)	8,592 (53.1)
2–4	21,277 (44.0)	14,032 (43.6)	7,245 (44.8)
>4	1,100 (2.3)	786 (2.4)	314 (1.9)
Processed meat (times/week)			
<1	18,301 (37.8)	11,802 (36.7)	6,499 (40.2)
1	14,018 (29.0)	9,207 (28.6)	4,811 (29.7)
≥2	15,890 (32.9)	11,067 (34.4)	4,823 (29.8)
Unprocessed red meat (times/week)
<2	23,094 (47.8)	15,274 (47.5)	7,820 (48.3)
2–4	20,611 (42.6)	13,682 (42.5)	6,929 (42.8)
>4	4,548 (9.4)	3,145 (9.8)	1,403 (8.7)
Vegetable (servings/day)			
<1.0	9,079 (18.8)	6,648 (20.7)	2,431 (15.0)
1.0–2.9	34,657 (71.7)	22,471 (69.8)	12,186 (75.3)
≥3.0	4,223 (8.7)	2,769 (8.6)	1,454 (9.0)
Fruit (servings/day)			
<2.0	17,741 (36.7)	12,973 (40.3)	4,768 (29.5)
2.0–3.9	22,337 (46.2)	14,277 (44.4)	8,060 (49.8)
≥4.0	8,130 (16.8)	4,815 (15.0)	3,315 (20.5)
Whole grain (servings/day)			
<1.0	22,325 (46.2)	16,041 (49.9)	6,284 (38.8)
1.0–2.9	19,675 (40.7)	12,137 (37.7)	7,538 (46.6)
≥3.0	5,829 (12.1)	3,625 (11.3)	2,204 (13.6)
Refined grain (servings/day)			
<1.0	26,332 (54.5)	16,623 (51.7)	9,709 (60.0)
1.0–2.9	16,241 (33.6)	11,368 (35.3)	4,873 (30.1)
≥3.0	5,256 (10.9)	3,812 (11.9)	1,444 (8.9)
Cheese (pieces/day)			
<2	19,953 (41.3)	13,222 (41.1)	6,731 (41.6)
2–4	21,177 (43.8)	14,032 (43.6)	7,145 (44.2)
>4	5,944 (12.3)	4,072 (12.7)	1,872 (11.6)
Coffee (cups/day)			
<1	14,705 (30.4)	10,030 (31.2)	4,675 (28.9)
1–2	18,319 (37.9)	11,546 (35.9)	6,773 (41.9)
≥3	15,168 (31.4)	10,466 (32.5)	4,702 (29.1)
Sugar-sweetened beverages consumer, *n* (%)	40,264 (83.3)	27,161 (84.4)	13,103 (81.0)
Healthy diet score	3.0 ± 1.4	2.8 ± 1.4	3.2 ± 1.4
GRS	417.2 ± 11.8	417.2 ± 11.8	417.3 ± 11.8

^a^ £1.00 = $1.30, €1.20

A total of 3,385 patients with T2D developed from prediabetes were documented during an average of 7.8 years of follow-up (378,437 person-years). The relationship between fish oil use and incident T2D is shown in Fig. 1B and Table S1. In the age- and sex-adjusted analysis (model 1), the use of fish oil supplements was inversely associated with the risk of T2D [hazard ratio (HR): 0.85, 95% confidence interval (CI): 0.79 to 0.92; *P* < 0.001]. After further adjustment for other demographic characteristics (model 2) and the use of other supplements and medications (model 3), the inverse association remained significant. The results also did not appreciably change after further adjustment for dietary factors, including consumption of oily fish, vegetables, and fruits (model 4). Finally, fish oil use was still significantly associated with a lower risk of T2D after adjustment for a healthy diet score instead of dietary components (model 5), which revealed a 9% (HR: 0.91, 95% CI: 0.84 to 0.99) lower risk of incident T2D (*P* = 0.021). The inverse association was more prominent in women (*P* interaction = 0.005) (Table S2). Importantly, sensitivity analyses showed that the documented significant inverse association did not substantially change after further adjustment for serum HbA1c levels, C-reactive protein (CRP) levels, lipid-lowering drugs, antihypertensive agents, or hormone replacement therapy and oral contraceptive use (Table S3). Our results also remained similar when we further excluded participants with extreme BMI, incident T2D cases that occurred within 2 years, persons who took any other supplements, or those with missing covariate data.

A previous study demonstrated that circulating fatty acids (FAs) have evident interactions with genetic predisposition to T2D and FA-associated variants [26]. Therefore, we subsequently detected the potential interactions between fish oil use and genetic factors in people with prediabetes. Notably, we did not observe a significant interaction between fish oil use and overall GRS on the risk of developing T2D (*P* interaction = 0.186) (Table S4), whereas there was a significant interaction between fish oil use and rs780094 in GCKR (*P* interaction = 0.049) and marginal interactions were found for rs174555 in FADS1 (*P* interaction = 0.078), rs7200543 in NTAN1 (*P* interaction = 0.061), and rs102275 in TMEM258 (*P* interaction = 0.072) (Table S5), indicating that DHA/EPA metabolic pathways may play a role in the beneficial effect of fish oil. Thus, we first validated the blood n-3 PUFAs elevating effect of fish oil supplementation (Table S6) and then assessed whether the relationship between plasma n-3 PUFAs and fish oil supplementation was modified by genotype at n-3 PUFA-related loci (Table S7). As expected, fish oil use was associated with higher plasma levels of n-3 PUFAs, DHA, and non-DHA n-3 PUFAs, and lower n-6/n-3 PUFA ratio (all *P* < 0.001). Notably, rs1077989 in TMEM229B had significant interactions with fish oil use on plasma levels of DHA (*P* interaction = 0.012) and n-3 PUFAs (*P* interaction = 0.036). The inverse relationship of fish oil supplementation with n-6/n-3 PUFA ratio was significantly modified by the number of n-3 PUFA-related alleles and several specific SNPs (all *P* interaction < 0.001).

### Skeletal muscle metabolome reveals a promotion of glucose disposal in response to DHA and EPA interventions

**Fig. 2.**
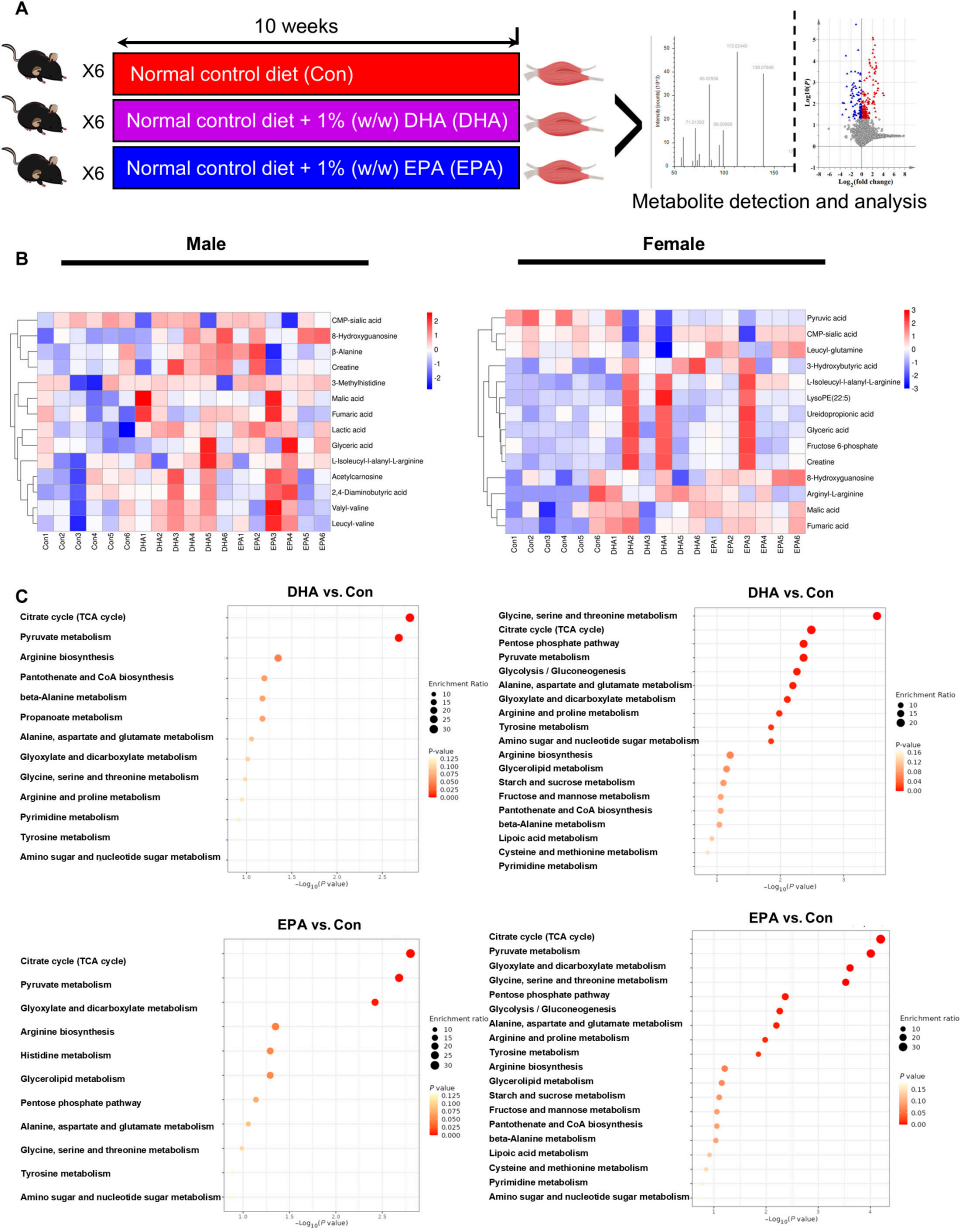
DHA and EPA alter the metabolome in skeletal muscle of *db/db* mice. (A) Flow chart of n-3 PUFA intervention animal study. Four-week *db/db* mice were fed normal control diet with or without DHA/EPA for 10 weeks, and the skeletal muscle was collected for the untargeted metabolome analysis. (B) Heatmaps of potential biomarkers whose VIP value >1. Color bars represent *Z* score. (C) Enrichment analysis of potential biomarkers whose VIP value >1. For (A) to (C), *n* = 6 for all groups. For (C), the sizes of circles represent enrichment ratio, and the shades of color represent *P* value.

Our human study in UK Biobank demonstrated that marine n-3 PUFAs from fish oils may exhibit an anti-diabetes effect in people with prediabetes. To further investigate the underlying mechanism, a long-term intervention with DHA or EPA (1% w/w in diets) was carried out in *db/db* mice. Skeletal muscle is the dominating tissue in glucose metabolism. In the present study, the DHA content and EPA content in skeletal muscle were significantly increased under DHA treatment and EPA treatment, respectively (Fig. S1A and B), which validated our dietary supplementations. We subsequently performed a nontargeted metabolomics analysis of skeletal muscles by ultrahigh-performance liquid chromatography (UHPLC)-Q-Orbitrap-high-resolution mass spectrometry (HRMS) analysis (Fig. 2A). There was no obvious separation in the unsupervised principal components analysis (PCA) plots between the control group and intervention groups (Fig. S2A). However, under DHA and EPA interventions, 257 and 369 metabolites were significantly regulated in female mice, while 262 and 252 metabolites significantly changed in male mice treated with DHA and EPA, respectively (Fig. S2B). Together, DHA and EPA interventions altered the metabolic profiles of skeletal muscle in diabetic mice, which may contribute to glucose disposal.

**Fig. 3.**
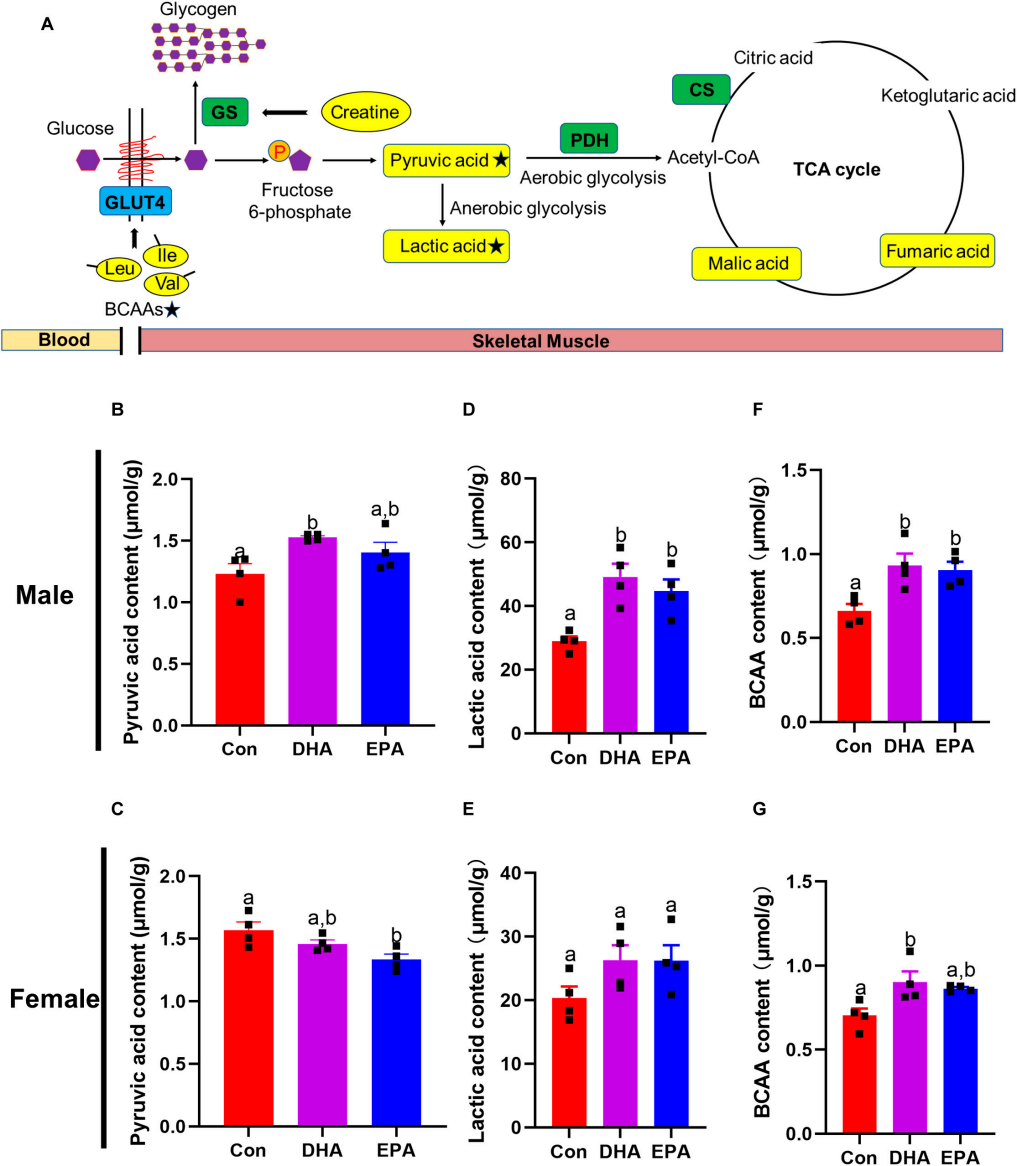
DHA and EPA regulate biomarkers related to glucose homeostasis. (A) Potential biomarkers (yellow background; VIP > 1) involved in glucose metabolism including glucose transport, glycogen synthesis, glycolysis, and TCA cycle. The black star shapes mark the significantly altered metabolites, which were further measured by assay kits. (B and C) Content of pyruvic acid in skeletal muscle. (D and E) Content of lactic acid in skeletal muscle. (F and G) Content of BCAAs in skeletal muscle. For (B) to (G), *n* = 4 for all groups. Data are presented as means ± SEM. Statistical differences were determined by one-way analysis of variance (ANOVA) followed by the Tukey’s multiple comparisons test. Groups with different superscript letters are significantly different (*P* < 0.05).

To further explore the metabolic biomarkers influenced by DHA and EPA, the discrimination between treatment groups and the control group was maximized by means of pairwise orthogonal projections to latent structures discriminant analysis (OPLS-DA) (Fig. S3A to D). Metabolites identified by Compound Discovery software in DHA and EPA groups were selected as potential metabolic biomarkers according to the threshold of variable importance in the projection (VIP) values >1.0 (Fig. 2B). Further Kyoto Encyclopedia of Genes and Genomes (KEGG) analysis demonstrated that those biomarkers were enriched in “TCA cycle” and “pyruvate metabolism” in both sexes (Fig. 2C). In these potential biomarkers with VIP > 1.0 (Fig. 3A), we found that the peak areas of pyruvic acid and lactic acid involved in glucose metabolism were significantly changed in female and male mice, respectively (Table S8). To further confirmed these changes, we directly measured these metabolite concentrations using assay kits. Results showed that EPA intervention significantly reduced the concentration of pyruvic acid in the skeletal muscle of female mice (Fig. 3B) and DHA intervention increased that in male mice (Fig. 3C). Considering that pyruvic acid is the key intermediary metabolite between glycolysis and tricarboxylic acid (TCA) cycle, n-3 PUFAs may accelerate the conversion of glycolysis into aerobic oxidation. In addition, DHA and EPA significantly increased the content of lactic acid, an end-product of anaerobic glycolysis, in skeletal muscle of male mice (Fig. 3D and E), suggesting the anaerobic glycolysis was promoted under n-3 PUFA intervention. Moreover, creatine was also selected as a potential biomarker under DHA and EPA treatments (Fig. 3A), which is pointed out as a regulator in glucose metabolism [27]. Studies on branched-chain amino acids (BCAAs) revealed that dietary intervention of BCAAs, especially leucine and isoleucine, decreased fasting blood glucose by promoting glucose metabolism in skeletal muscle of rats [28]. Additionally, we noted that many screened potential biomarkers in DHA and EPA groups were BCAA derivatives (Fig. 2B and Table S8). Therefore, we also directly measured total BCAA levels, including leucine, isoleucine, and valine, and observed that DHA and EPA significantly enhanced the content of total BCAAs in skeletal muscle of male mice and DHA increased that in female mice (Fig. 3F and G). Altogether, these data suggested that DHA and EPA treatment may promote the glucose disposal, which was consistent with the decreased risk of T2D in people with prediabetes who used to take fish oil supplements. Additionally, among people with prediabetes, we found that plasma n-3 PUFAs levels were inversely associated with plasma pyruvate (*P* = 0.006) and lactate (*P* = 0.010) levels and positively related to isoleucine and leucine levels (both *P* < 0.001) (Table S9).

Considering the potential influence of outlier animals on metabolome, sensitivity analyses were performed by excluding outliers. After exclusion, we still found that important metabolites in glycolysis and TCA cycle such as pyruvic acid, lactic acid, malic acid, and fumaric acid, BCAA derivatives including leucyl-valine and l-isoleucyl–l-alanyl–l-arginine, and creatine were selected as biomarkers in OPLS-DA models (Table S10).

### DHA and EPA promote aerobic glycolysis and glycogen synthesis in diabetic skeletal muscle and IR myotubes

**Fig. 4.**
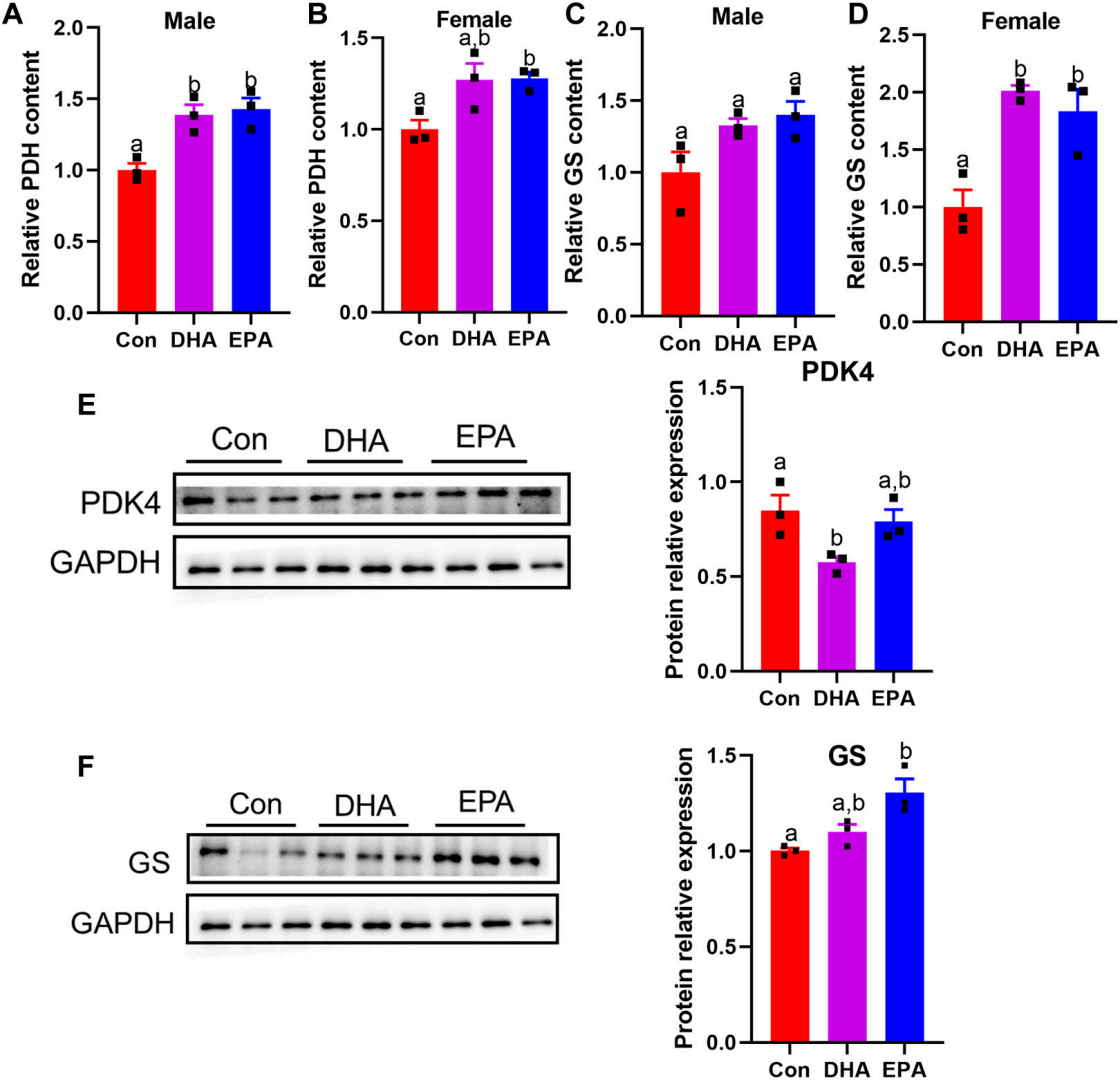
DHA and EPA regulate glycolysis and glycogen synthesis in vivo and in vitro*.* (A and B) ELISA analysis for muscular PDH in male and female *db/db* mice. (C and D) ELISA analysis for muscular GS in male and female *db/db* mice. (E and F) Representative immunoblots for the protein expression of (E) PDK4 and (F) GS in insulin-resistant myotubes. For (A) to (F), *n* = 3 for all groups. Data are presented as means ± SEM. Statistical differences were determined by one-way ANOVA followed by the Tukey’s multiple comparisons test. Groups with different superscript letters are significantly different (*P* < 0.05).

Considering that KEGG analysis of metabolome showed enrichment of glycolysis and TCA cycle in DHA and EPA groups, we then speculated that the process linking the 2 glucose metabolism pathways might be promoted. Consequently, we next examined the content of pyruvate dehydrogenase (PDH) in skeletal muscle, which mediates the transition from pyruvate to acetyl-coenzyme A (CoA). As expected, the content of PDH was significantly enhanced by DHA and EPA supplementation in male mice, while only the EPA treatment significantly elevated PDH content in female mice (Fig. 4A and B). To further explore the underlying mechanism, we measured the pyruvate dehydrogenase kinase 4 (PDK4) expression of myotubes, which was recognized as an inactivator of PDH. Intriguingly, DHA treatment significantly decreased the expression of PDK4, whereas no significant change was observed in the EPA group compared with the control (Fig. 4E). Given that activation of the insulin signal pathway greatly promotes glucose metabolism, we further investigated the effects of DHA and EPA on glycolysis and TCA cycle under acute insulin stimulation. DHA and EPA treatments significantly enhanced the activities of PDH and citrate synthase (CS) in response to insulin (Fig. S4A to D), which demonstrated that DHA and EPA treatments promoted the glucose oxidation through TCA cycle.

Most of the glucose absorbed by myocytes is used to synthesize glycogen under insulin stimulation [29]. In addition, previous animal and human studies demonstrated that the supplementation of creatine increased glycogen contents in skeletal muscle [27]. Notably, creatine as a potential biomarker in DHA and EPA groups indicated the activation of glycogen synthesis (Fig. 3A). Further enzyme-linked immunosorbent assay (ELISA) examination in skeletal muscle proved a significant increase in glycogen synthase (GS) contents in DHA and EPA groups (Fig. 4C and D). In addition, under insulin stimulation, EPA treatment significantly enhanced the glycogen level in skeletal muscles in both sexes and DHA treatment significantly increased glycogen level in female mice (Fig. S4E and F), indicating increased glucose uptake and glycogen synthesis. Moreover, the effects of EPA on GS were further corroborated in vitro (Fig. 4F), revealing that marine n-3 PUFAs may promote muscle glycogen synthesis by elevating GS expression.

### DHA and EPA promote glucose uptake in the skeletal muscle of *db/db* mice

**Fig. 5.**
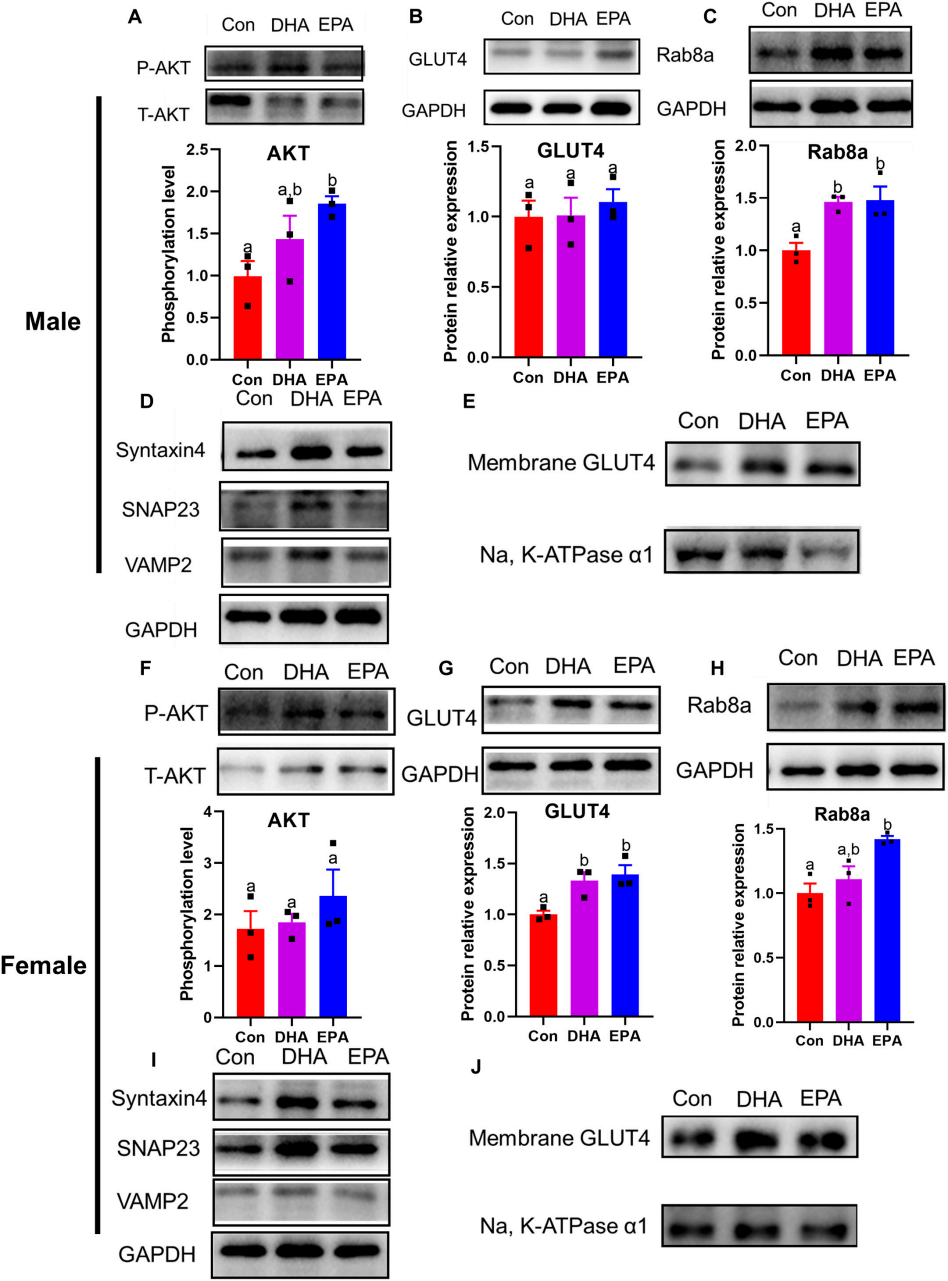
DHA and EPA regulate key proteins on GLUT4 translocation and increase membrane GLUT4 in skeletal muscle. (A and F) Representative immunoblots for the protein expression of AKT phosphorylation. (B and G) Representative immunoblots for the protein expression of GLUT4. (C and H) Representative immunoblots for the protein expression of Rab8a. (D and I) Representative immunoblots for the protein expressions of SNAREs including syntaxin4, SNAP23, and VAMP2. (E and J) Representative immunoblots for the protein expressions of membrane GLUT4 in male and female *db/db* mice. For (A) to (J), *n* = 3 for all groups. Data are presented as means ± SEM. Statistical differences were determined by one-way ANOVA followed by the Tukey’s multiple comparisons test. Groups with different superscript letters are significantly different (*P* < 0.05).

In the present study, we found that DHA and EPA enhanced glucose disposal by elevating aerobic glycolysis and glycogen synthesis in skeletal muscle, suggesting a promotion in glucose uptake. In addition, BCAAs and their dipeptides can stimulate glucose uptake by increasing muscular membrane GLUT4 concentrations, indicating that GLUT4 translocation has been promoted [30,31]. Notably, we also observed an increase in BCAA levels in the skeletal muscle of *db/db* mice fed with n-3 PUFAs (Fig. 3F and G). It is tempting to speculate that GLUT4 vesicle trafficking may be promoted in skeletal muscles of DHA- and EPA-supplemented groups. Therefore, we next investigated the protein alterations related to GLUT4 translocation. As shown in Fig. 5A, in male mice, EPA treatment significantly advanced the phosphorylation of AKT. However, there seemed not to be an enhancement of GLUT4 expression (Fig. 5B), suggesting that n-3 PUFAs improve glucose metabolism by promoting GLUT4 translocation rather than protein expression. To confirm this hypothesis, the key proteins in GLUT4 translocation according to the SNARE theory were further detected. As anticipated, the expression of Rab8a, which plays a dominant role in GLUT4 vesicle trafficking from perinuclear region to the plasma membrane in skeletal muscle, was significantly improved in DHA- and EPA-treated male mice (Fig. 5C). Moreover, DHA treatment significantly promoted SNAP23 expression in skeletal muscle of males (Fig. 5D and Fig. S5A). Furthermore, the increased membrane GLUT4 under insulin stimulation in DHA and EPA groups confirmed the promotion of GLUT4 translocation (Fig. 5E and Fig. S5B). In females, we found a significant increase in GLUT4 expression in DHA- and EPA-treated *db/db* mice, although no significant change in phosphorylation of AKT was observed in these groups (Fig. 5F and G). Similar to males, EPA supplementation significantly enhanced Rab8a expression in females (Fig. 5H). Notably, even the SNARE proteins were not significantly altered under DHA and EPA treatments, and membrane GLUT4 level was significantly enhanced in muscles, suggesting that n-3 PUFA also regulated GLUT4 translocation in female mice (Fig. 5I and J and Fig. S5C and D). Together, the immunoblot results in skeletal muscle demonstrated that DHA and EPA may promote GLUT4 translocation by the vesicle trafficking process, and further advance glucose uptake and disposal. Consistently, we observed significant interactions of fish oil use with variants at GLUT4 (rs5435 in women and rs8082645 in men) on T2D risk (*P* interaction = 0.030 for rs5435 and 0.047 for rs8082645) among people with prediabetes (Table S11), reinforcing GLUT4 activation as the potential target of n-3 PUFA supplementation in T2D prevention.

### DHA and EPA promote GLUT4 translocation in myotubes

**Fig. 6.**
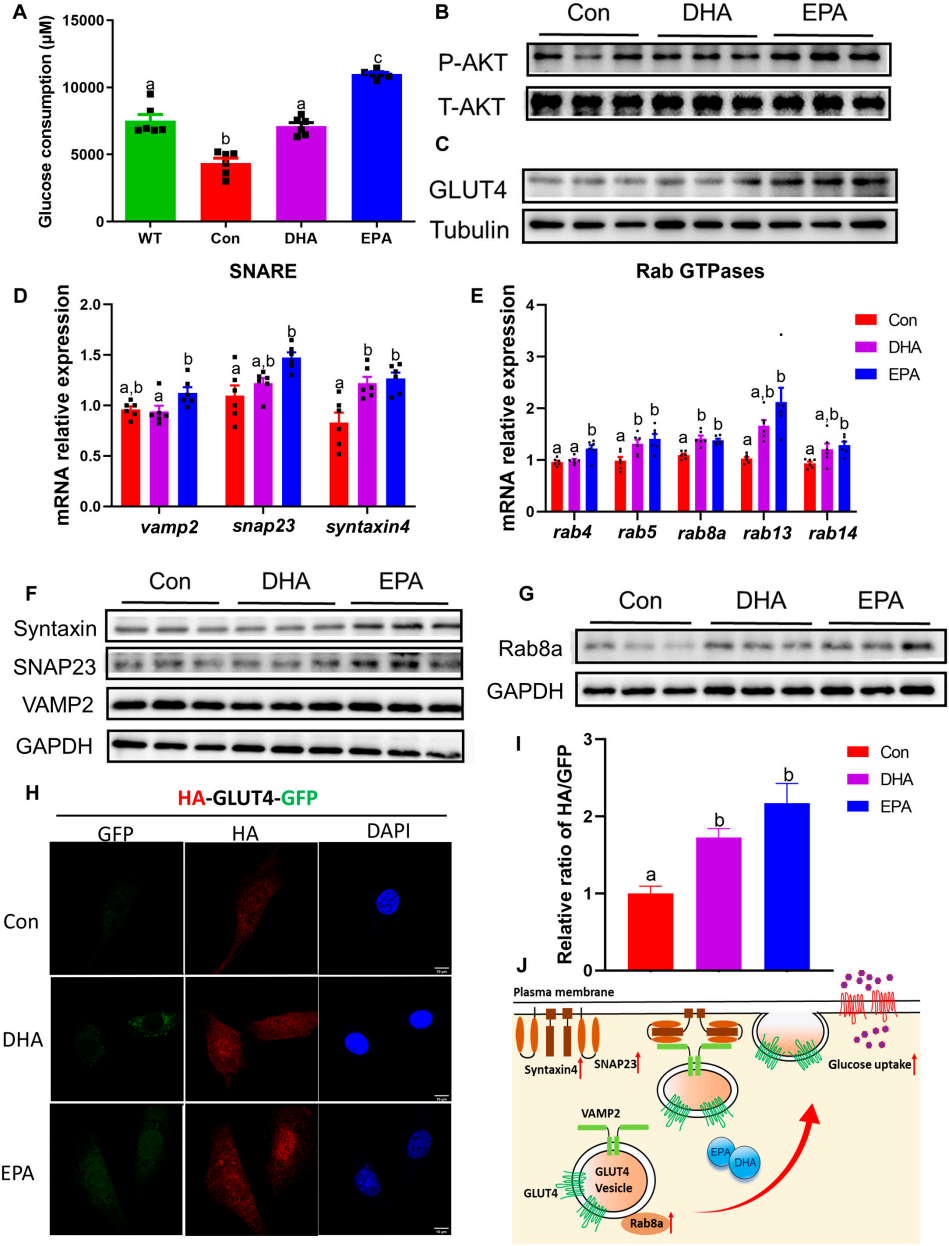
DHA and EPA promote GLUT4 translocation in insulin-resistant C2C12 cells. (A) Glucose consumption in normal C2C12 cells [wild-type (WT)] and insulin-resistant C2C12 cells induced by palmitic acid. (B and C) Representative immunoblots for the protein expression of AKT phosphorylation and GLUT4. (D and E) qPCR analysis for the mRNA expression of (D) SNARE genes and (E) Rab GTPases. (F and G) Representative immunoblots for the protein expression of (F) SNAREs and (G) Rab8a. (H) Immunofluorescence of total GLUT4 (green) and plasma membrane GLUT4 (red). (I) Ratio of fluorescence from exposed HA epitope staining and GFP in C2C12 cells under insulin stimulation. (J) Diagram depicting the process of GLUT4 translocation promoted by DHA and EPA. For (A), (D), and (E), *n* = 6 for all groups. For (B), (C), (F), and (G), *n* = 3 for all groups. For (H) to (I), *n* ≥ 10 for all groups. Data are presented as means ± SEM. Statistical differences were determined by one-way ANOVA followed by the Tukey’s test (for balanced designs) or the Tukey–Kramer method (for unequal sample sizes). Groups with different superscript letters are significantly different (*P* < 0.05).

Considering the beneficial role of DHA and EPA in promoting GLUT4 translocation, we next interrogated whether and how DHA and EPA were functionally involved in glucose metabolism in C2C12 myotubes, which were in an IR state induced by palmitic acid. Compared with normal cultured myotubes in the wild-type group, impaired glucose disposal in insulin-resistant cultured myotubes was observed and expectedly rescued by DHA and EPA treatments (Fig**.** 6A). In line with better phenotypes of blood glucose and HbA1c levels in EPA-treated diabetic mice [32], the increase of glucose consumption in the EPA treatment group was significantly higher than that in the DHA treatment group. Meanwhile, compared with control myotubes, phosphorylation of AKT was significantly promoted in the EPA treatment group under insulin stimulation, coupled with evidently increased GLUT4 expression, suggesting that the insulin signaling pathway was eventually activated (Fig**.** 6B and C and Fig. S6A and B). Moreover, there was no significant change in AKT phosphorylation in DHA-treated myotubes, which further highlighted the better performance of EPA treatment in alleviating hyperglycemia. However, there was no significant change in glucose transporter 1 (GLUT1), another glucose transporter in basal state (Fig. S7A and B). In addition, DHA and EPA significantly decreased tumor necrosis factor-α (TNF-α) expression, while DHA also down-regulated interleukin-6 (IL-6) expression (Fig. S6C and D), suggesting that n-3 PUFA also alleviated inflammation in insulin-resistant myotubes.

Given that the expression of GLUT4 traffic-related protein such as Rab8a was up-regulated in diabetic mice fed with DHA and EPA, indicating that the GLUT4 vesicle trafficking may be promoted, we further explored whether and how the translocation of GLUT4 vesicles was regulated by DHA and EPA interventions in myotubes. The quantitative polymerase chain reaction (qPCR) analyses showed that EPA treatment significantly increased the mRNA expression of *snap23* and *syntaxin4* in IR myotubes under insulin stimulation, while DHA treatment only enhanced the expression of syntaxin4 (Fig. 6D). Similarly, the mRNA levels of Rab GTPases including *rab4*, *rab5*, *rab8a*, *rab13*, and *rab14* were extensively elevated in EPA-treated myotubes. Among these, *rab5* and *rab8a* were significantly up-regulated by DHA intervention (Fig. 6E). To validate the difference of GLUT4 vesicle trafficking regulation between DHA and EPA treatment, we then further performed the protein assessments and exhibited a significant increase in t-SNARE proteins including SNAP23 and syntaxin4 in EPA- but not DHA-treated myotubes (Fig. 6F and Fig. S6E). Remarkably, EPA treatment significantly elevated the protein expression of Rab8a in myotubes, while no significant change was found in the DHA group (Fig. 6G and Fig. S6F). To further observe the profile of GLUT4 translocation, hemagglutinin (HA)–GLUT4–green fluorescent protein (GFP) was transfected into the myoblast, which contains an HA epitope exposed on the cell surface when GLUT4 vesicles have fused with the plasma membrane. In the basal state, the HA fluorescence (red) on the surface of cell membranes was overall too weak to observe any difference between groups (Fig. S8), whereas DHA and EPA significantly increased the cellular surface levels of GLUT4 in myoblasts under insulin stimulation (Fig. 6H and I). In addition, there was a similar tendency in mRNA and protein expressions of Rab GTPases and SNAREs (except syntaxin4) between basal and insulin stimulation state (Fig. 6D to G and Fig. S7A and C to F), indicating that n-3 PUFAs start to play a regulatory role on GLUT4 translocation when cells have not yet been stimulated by insulin. Together, our results suggested that n-3 PUFAs promoted the traffic of GLUT4 to the plasma membrane in skeletal muscle by increasing Rabs and t-SNARE expressions, which further enhanced the glucose uptake and improved glucose homeostasis (Fig. 6J).

## Discussion

In the current study, we observed that fish oil supplementation was related to a 9% lower risk of T2D among people with prediabetes after adjustment for multivariable risk factors for T2D development. The inverse relationship was modified by rs780094 in GCKR and variants at GLUT4. Consistently, both DHA and EPA supplementation alleviated hyperglycemia in diabetic mice. Considering the dominant role of skeletal muscle in glucose metabolism, further skeletal muscle metabolomics study revealed that the marine n-3 PUFAs effectively altered biomarkers such as BCAAs, creatine, and other intermediate metabolites involved in glycolysis and TCA cycle, indicating that DHA and EPA may reduce blood glucose concentrations by regulating glucose uptake, aerobic glycolysis, and glycogen synthesis. Furthermore, subsequent protein immunoblots revealed the underlying role of DHA and EPA treatment in improving glucose metabolism by promoting GLUT4 vesicle translocation and PDH and GS expressions, which was further confirmed in myotubes. Notably, EPA showed a better ability than DHA in rescuing hyperglycemic phenotype by regulating t-SNAREs and Rab GTPases in the GLUT4 vesicle trafficking process. Collectively, these results suggested that DHA and EPA interventions promote GLUT4 translocation and restore glucose homeostasis in skeletal muscle, manifesting a therapeutic potential for improving hyperglycemia.

Previous cohort studies about the association of n-3 PUFA intake with the incidence of T2D have reported inconsistent results. An observational study in China reported an inverse relationship between marine n-3 PUFA intake and T2D incidence in men (HR for 0.20 g/day versus 0.02 g/day = 0.84 [0.74 to 0.95]) [33]. Similarly, a Japanese study found that men in the highest quartile of fish intake had a 27% lower risk of T2D compared with the lowest quartile (median intake: 171.7 versus 36.6 g/day) [34]. However, in a prospective cohort study of 4,472 Dutch participants, dietary intake of long-chain n-3 PUFAs was not related to the risk of T2D (HR for ≥0.15 g/day versus ≤0.05 g/day = 1.05 [0.80 to 1.38]) [35]. Similarly, 2 other studies in Europe also did not find any significant association [36,37]. Moreover, 2 large-scale studies in America showed an increased risk of T2D associated with n-3 PUFA intake [38,39]. Notably, our current study based on 48,358 people with prediabetes from the UK Biobank, to the best of our knowledge, is the first to demonstrate that habitual use of fish oil supplements has a protective effect on developing T2D in a large prospective cohort. Importantly, lots of reliable RCTs are consistent with our findings. A trial in Italy (*n* = 281) showed that taking 3 g of n-3 PUFAs per day for 18 months reduced glycemia and subsequently decreased the risk of developing T2D (HR: 0.19, 95% CI: 0.09 to 0.40) in patients with impaired glucose metabolism (IGM) [40]. Another trial also found that a 6-month EPA treatment corrected hyperglycemia and postprandial insulin secretory ability among patients with IGM [41]. Nonetheless, in a large RCT of patients with multiple cardiovascular disease (CVD) risk factors, supplementation of EPA plus DHA (0.86 g/day) for 5 years did not significantly reduce the occurrence of T2D [42]. The recent meta-analysis of 17 RCTs concluded that n-3 PUFA could not lower the risk of developing T2D [9]. Some other trials even revealed that n-3 PUFA supplementation at a high dose (>4.4 g/d) could worsen glucose metabolism [9,43]. Conflicting results from previous RCTs could be due to different doses of fish oil supplements, treatment duration, insufficient sample sizes, and use of hypoglycemic drugs or insulin therapy, which could mask the effects of fish oil supplementation in patients with hyperglycemia. Importantly, previous RCTs had a limited sample size and follow-up duration so they could not observe sufficient incident T2D cases. Based on large sample size and long-term follow-up, our study provided sufficient statistical power to detect a robust inverse relationship of fish oil use with T2D risk. Besides, we also explored the interaction between fish oil use and genetic risk of T2D. Paramount evidence concluded that fish oil supplementation was related to a lower risk of T2D among hyperglycemia individuals, regardless of the overall genetic predisposition. However, we found that the risk-lowering effect of fish oil was modified by rs780094 in GCKR. C-allele of GCKR rs780094 was associated with lower circulating docosapentaenoic acid(DPA) levels and was also related to glycemic dysfunction [44,45], which might be in need of n-3 supplementation. Our results suggested that participants with more C-alleles of GCKR rs780094 may derive more benefits from fish oil supplementation. Overall, participants with fewer n-3 PUFA-related alleles seemed to have a more prominent increase in plasma n-3 PUFAs levels or n-3/n-6 PUFA ratio upon fish oil supplementation. This might be due to the low basal level of plasma n-3 PUFAs among participants lacking in n-3 PUFA-related alleles.

In accordance with the present finding in humans, a previous study showed that long-term intervention with pure DHA or EPA significantly reduced blood glucose and HbA1c, improved glucose tolerance, and elevated insulin sensitivity in *db/db* mice [32]. Similar results were shown in another intervention experiment that n-3 PUFAs effectively mitigated hyperglycemia in diet-induced obese rodents [5]. Considering the dominance of skeletal muscle in glucose disposal, n-3 PUFAs may modulate muscular glucose metabolism to maintain whole-body glucose homeostasis. Nevertheless, few studies have focused on the relationship between n-3 PUFA intake and glucose metabolism in skeletal muscle, and the mechanism underlying hypoglycemic effects remains to be elucidated. Therefore, we collected the muscle tissues from *db/db* diabetic mice in the n-3 PUFA intervention experiment and performed muscular metabolome analysis to investigate how DHA and EPA regulated glucose homeostasis through the skeletal muscle. In aerobic glycolysis, phosphorylated glucose is converted to pyruvate, 90% of which is oxidized to acetyl-CoA by PDH and then enters TCA cycle for oxidation [29]. It was previously reported that fish oil gavage (2 g/kg body weight) attenuated IR coupled with improved muscular TCA cycle intermediates, including citrate, α-ketoglutarate, malate, and oxaloacetate in mice [46]. In agreement, in the present study, n-3 PUFA supplementation accelerated glycolysis and TCA cycle by advancing fructose 6-phosphate, malic acid, and fumaric acid in *db/db* mice, while the declined pyruvate levels, which were also detected in people with prediabetes, suggested elevating the PDH-mediated process from glycolysis to TCA cycle. Previous evidence demonstrated that fish oils enhanced the activity of PDH in pancreatic islets and improved IR induced by a sucrose-rich diet in rats [47]. Other studies demonstrated that PDH and CS play a key role in glucose oxidation [48,49], of which activities were significantly enhanced in DHA and EPA groups under insulin stimulation in our study. Notably, PDK4 phosphorylates and inactivates PDH levels in skeletal muscle, which is positively associated with IR [50]. Similarly, our cellular experiments showed that DHA and EPA treatments enhanced the activity of PDH in diabetic skeletal muscle, which could be due to the down-regulation of PDK4.

In skeletal muscle, glycogen synthesis is a principal pathway of non-oxidative glucose disposal, and about 70% of glucose enters glycogen synthesis under postprandial high-level insulin [18], while impaired muscle glycogen synthesis is a core defect in T2D [51]. Creatine supplementation has been reported to stimulate muscle glycogen storage in the skeletal muscle of rats [52]. A human study in Korea demonstrated that lower levels of serum creatine were significantly related to higher risk of T2D in men [53]. In addition, l-leucine, a regulator of energy balance [54], facilitates glycogen synthesis by inactivating glycogen synthase kinase-3, which suppresses the activity of GS and blocks glycogen synthesis [55]. In the present study, the leucine derivatives and creatine were screened as potential biomarkers in the skeletal muscle of n-3 PUFA-treated mice, indicating that DHA and EPA may improve glucose uptake and glycogen synthesis. Furthermore, previous evidence suggested that fish oil supplementation increases glycogen content in the liver and muscle of mice coupled with impaired glucose tolerance induced by trimethylamine N-oxide [56]. Consistently, in the present study, EPA treatment significantly increased glycogen contents of skeletal muscle in both sexes, while DHA treatment significantly increased muscle glycogen contents in female mice under insulin stimulation. Furthermore, we found that n-3 PUFA treatment up-regulated the expression of GS both in vivo and in vitro. In addition, the activity of GS is promoted by phosphorylated AKT [57], which was enhanced by EPA intervention in the current study.

BCAAs, facilitators in improving glucose homeostasis, have been proven to promote AS160 phosphorylation by activating PI3K and AKT, which further release the inhibition on Rab GTPases and enhance GLUT4 translocation [31]. Similarly, isoleucine supplementation increased cellular 2-deoxyglucose uptake along with the enhancement of muscular membrane concentrations of GLUT1 and GLUT4 in C2C12 myotubes [58]. Importantly, the current study showed that the content of BCAAs in skeletal muscle was significantly increased by DHA and EPA in male mice, suggesting the GLUT4 translocation may be promoted. In concert, our human study showed increased isoleucine and leucine levels and significant interactions with GLUT4 for fish oil use. Notably, in subsequent immunoblotting analysis, we found that both DHA and EPA elevated the expression of Rab8a in male mice, which drives the departure of GLUT4 vesicles from the perinuclear region, based on gain- and loss-of-function experiments [59,60].

Interestingly, we found that n-3 PUFAs did not significantly increase GLUT4 expression in all cases, such as DHA-treated C2C12 myotubes and DHA- and EPA-treated male mice, which further suggested that n-3 PUFAs alleviated hyperglycemia by promoting GLUT4 translocation rather than depending on increased GLUT4 expression. Consistent with this speculation, the content of GLUT4 on membrane was significantly elevated in DHA- and EPA-treated mice under insulin stimulation. According to the SNARE hypothesis, which expounds a range of SNARE protein-mediated processes in GLUT4 transport from vesicles to plasma membrane [61], we further revealed how DHA and EPA promoted the GLUT4 translocation in skeletal muscle. The deficiency of t-SNAREs leads to inhibiting GLUT4 traffic into the membrane under insulin stimulation, while sufficient t-SNAREs increase membrane GLUT4 and improve glucose metabolism [24,62]. Our previous study has shown that n-3 PUFAs accelerated docking and fusion between vesicles and membrane by specifically enhancing the expression of VAMP2, the v-SNARE protein, in 3T3-L1 adipocytes [63]. Inconsistently, in the current study of myotubes, both DHA and EPA treatments did not alter VAMP2 expression, but EPA promoted the expression of syntaxin4 and SNAP23, the t-SMARE proteins, suggesting that the tethering, docking, and fusion of the GLUT4 vesicles to the plasma membrane were promoted. In agreement with our hypothesis, a previous study demonstrated that fish oil containing 43.8% DHA and 8.6% EPA supplemented with 4% taurine accelerated the GLUT4 distribution in the plasma membrane of the skeletal muscle of diabetic mice [64], but did not further point out the molecular role in the vesicle trafficking process. As originally hypothesized, we demonstrated that DHA and EPA reversed the impaired glucose consumption in insulin-resistant myotubes and promoted the GLUT4 translocation to plasma membranes visualized by the immunofluorescence imaging. Furthermore, DHA and EPA treatments significantly elevated Rab8a expression in mRNA level. Besides Rab8a, other GTPases have been reported to be involved in GLUT4 translocation. A recent study showed that loss-of-function mutation of Rab5 suppressed GLUT4 translocation and glucose uptake in myoblasts and myotubes under insulin stimulation [65]. In addition, another study demonstrated that Rab13 insulin-dependently localized GLUT4 vesicles at the muscular cell membrane by binding to MICAL-2 and α-actinin-4 [66]. Moreover, *Rab4* and *Rab14* mediate the replenishment of GLUT4 vesicles from sorting endosomes [67,68]. Here, we found that EPA up-regulated the mRNA expression of *Rab4*, *Rab5*, *Rab13*, and *Rab14*, while DHA only elevated the expression of *Rab5* and *Rab13*. Taken together, DHA and EPA manipulate GLUT4 vesicle trafficking by regulating various Rab proteins as small GTPases, while EPA exhibited a better alleviative effect.

In our human study, we detected that the protective relationship of fish oil use with T2D risk mainly existed in female people with prediabetes. The interaction of fish oil use with the variant at GLUT4 was more prominent in women than in men. In our mouse study, although we detected that muscular glucose metabolism was improved in both male and female mice after DHA/EPA supplementation, up-regulations in GLUT4 and GS were more pronounced in females. Moreover, DHA/EPA-treated female mice had a greater increase in PDH activity in response to insulin compared with male mice. The sex hormones and sex-specific absorption of FAs may account for the sex difference [69,70]. Associations detected for metabolites in the human study were generally congruent with results from our mouse study (decrease in pyruvate in females and increase in BCAAs), whereas associations documented in humans were not sex-specific and the direction of change in lactate in humans seemed to be opposite from that in mice. One possible explanation could be that these metabolites were detected in plasma in humans but measured in muscles in mice, and thus, changes may not be synchronized. Moreover, levels of metabolites were detected in humans at baseline and could not reflect long-term changes after fish oil supplementation. Additional human study is needed to assess sex differences in muscular glucose metabolism in response to n-3 PUFAs.

N-3 PUFAs, as a constituent of membrane phospholipids, have a remarkable contribution to the physical properties of biological membranes. Due to differences in acyl chain length and unsaturation degree, DHA and EPA could have different effects on membrane structure and function [71]. These different effects on membrane structure may partially explain the difference between DHA and EPA on glucose metabolism regulation. However, the underlying mechanism merits further investigation. Previous evidence revealed that EPA activated peroxisome proliferator-activated receptor (PPAR), which mediated the oxidation of FAs in the liver and exhibited a better anti-obesity effect than DHA [72], whereas DHA more effectively induced a browning process and promoted lipid metabolism in white adipose tissue [73], which indicated potential differences between DHA and EPA in regulating metabolic homeostasis. Intriguingly, it has been reported that EPA exhibited lower serum glucose and HbA1c in male mice compared with that in DHA-treated mice [32]. Moreover, EPA, but not DHA, also evidently up-regulated syntaxin4 and SNAP23 levels in myotubes. Combined with the better promotion of EPA in Rab GTPase expression, EPA may have a more promising hypoglycemic effect than DHA.

Taken together, our results demonstrated that marine n-3 PUFAs from fish oils lower the risk of T2D development in people with prediabetes and interacted with variants at GLUT4, which may owe to the improvement of glucose homeostasis in skeletal muscle. Specifically, DHA and EPA, major n-3 PUFA ingredients, promoted oxidative metabolism of glucose and glycogen synthesis in skeletal muscle of diabetic mice. These results offer an in-depth understanding of the causal relationship and underlying mechanisms of the alleviative effects of marine n-3 PUFAs on hyperglycemia in humans. Moreover, DHA and EPA up-regulated the expression of Rab GTPases and t-SNAREs, which mediated the approaching and fusing process of GLUT4 vesicles with plasma membranes, and further promoted blood glucose transfer into skeletal muscle, suggesting that marine n-3 PUFAs mitigate hyperglycemia by advancing GLUT4 vesicle translocation. Intriguingly, we also noticed that EPA performed a better hypoglycemic effect than DHA. Overall, our findings offer new robust evidence on the beneficial effects of marine n-3 PUFAs on T2D prevention by targeting muscular glucose homeostasis, opening up new research avenues into pioneer treatment for enhancing GLUT4 translocation, glycogen synthesis, and aerobic glycolysis.

## Materials and Methods

A total of 48,358 people with prediabetes from the UK Biobank were selected for the analysis of the relationships between fish oil supplementation and incident T2D in our human study.

The study design was approved by the North West Multi-Centre Research Ethics Committee (reference number 06/MRE09/65). All the participants were required to provide written informed consent. Mouse and cell models were used in this study to reveal the mechanisms of marine n-3 FA treatment for mitigating hyperglycemia in prediabetes by improving muscular GLUT4 translocation and glucose homeostasis. Animal experiments were approved by the Institutional Animal Care and Use Committee of Zhejiang University School of Medicine (approval ID: ZSLL-2018-016). For a detailed description of materials and methods, see Supplementary Methods.

## Data Availability

All data are available in the main text or the Supplementary Materials. Additional data related to this paper may be requested from the authors.

## Supplementary Materials

Supplementary 1 Detailed Methods Figs. S1 to S9 Tables S1 to S19
