# Supplementary material for: Marine N-3 Fatty Acids Mitigate Hyperglycemia in Prediabetes by Improving Muscular Glucose Transporter 4 Translocation and Glucose Homeostasis
Source: Research (Wash D C). 2025 Apr 29;8:0683. doi: 10.34133/research.0683 (PMC12038161; doi:10.34133/research.0683)
Supplement: Supplementary 1 — Detailed Methods Figs. S1 to S9 Tables S1 to S19 [file research.0683.f1.zip › Revised Suppl Mater-Li et al-Research.docx]

Supplementary Material

Marine n-3 fatty acids mitigate hyperglycemia in prediabetes by improving muscular GLUT4 translocation and glucose homeostasis

**Detailed Methods**

**Supplementary Figures**

**Figure S1.** DHA and EPA contents of skeletal muscles in male and female *db/db* mice.

**Figure S2.** Metabolome analysis of skeletal muscle in male and female *db/db* mice.

**Figure S3.** OPLS-DA analysis of muscular metabolome in male and female *db/db* mice.

**Figure S4.** Effects of DHA and EPA on glycogen synthesis and glucose oxidation in skeletal muscles under insulin stimulation.

**Figure S5.** Quantitative results of Western blotting analysis in *db/db* mice.

**Figure S6.** The expression of markers in insulin signaling and inflammation in insulin-resistant C2C12 cells.

**Figure S7.** DHA and EPA regulate GLUT4 translocation in insulin-resistant C2C12 cells without insulin stimulation.

**Figure S8.** Immunofluorescence of GLUT4 in C2C12 cells.

**Figure S9.** Flow of participants in the current UK biobank study.

**Supplementary Tables**

**Table S1.** HR (95% CI) of type 2 diabetes according to fish oil use in the UK Biobank in prediabetes patients.

**Table S2.** HR (95% CI) of type 2 diabetes according to fish oil use in men and women with prediabetes

**Table S3.** HR (95% CI) of type 2 diabetes in prediabetes in sensitivity analyses.

**Table S4.** Interactions of fish oil with GRS for T2D.

**Table S5.** Interactions between fish oil and n-3 PUFAs-associated alleles on T2D risk.

**Table S6.** Associations of fish oil use with plasma fatty acids and metabolites.

**Table S7.** Interactions between fish oil and n-3 PUFAs-associated SNPs on plasma levels of n-3 PUFAs and n-6/n-3 PUFAs ratio (*n*=10267).

**Table S8.** The *P* value and FDR of potential biomarkers (VIP>1 for EPA/DHA group) in male and female mice.

**Table S9.** Associations of plasma n-3 PUFAs with pyruvate, lactate, isoleucine and leucine.

**Table S10.** The biomarkers of each group in metabolome analysis from OPLS-DA models excluding outlier animals.

**Table S11.** Interactions between fish oil use and SNPs of GLUT4, GYS1/2, GSK3β and PDPR on T2D risk.

**Table S12.** Spearman correlations between fish oil use assessed at baseline and in resurvey.

**Table S13.** Diet component definitions used in the UK Biobank study.

**Table S14.** Definitions of prevalent diabetes and incident T2D.

**Table S15.** Characteristics of 424 T2D-associated SNPs in the UK biobank.

**Table S16.** Single-nucleotide polymorphisms associated with circulating n-3 PUFAs.

**Table S17.** Diet composition of each animal group.

**Table S18.** qPCR primers.

**Table S19.** Antibodies used in this study.

**Detailed Methods**

**Human study**

The UK Biobank Study is a large prospective cohort consisting of about 0.5 million men and women aged 40‒69 years and recruited across the UK from 2006 to 2010 [1, 2]. At baseline, participants were required to complete a series of touch-screen questionnaires, provide biological samples and undergo various physical assessments. All participants gave informed consent at recruitment. The study design was approved by the North West Multi-Centre Research Ethics Committee (reference number 06/MRE09/65). The UK Biobank dataset for this project included 502,505 participants, and 60,577 were diagnosed with prediabetes. We excluded participants who subsequently withdrew, patients with CVD or cancer at baseline, and those who lacked data on fish oil use. Then, 48,358 people with prediabetes were selected for the analysis of the relationships between fish oil supplementation and incident T2D. To explore gene-nutrient interaction, we further excluded participants with no genetic data and who were not of White British descent, and 42909 persons were selected for the gene-nutrient interaction analysis (Figure S9).

**Assessment of fish oil use and covariates**

In assessment centers, participants completed a touch-screen questionnaire which included the question: “Do you regularly take any of the following?” They could select more than one answer from a list of supplements including fish oils. Two repeated surveys conducted in 16,469 and 41,612 participants, respectively, were used to assess the reproducibility and validity of fish oil use. In the first repeated survey completed from 2012 to 2013, 72.5% of fish oil users at baseline reported that they continued to use fish oil supplements (Spearman *r*=0.61), and in the second repeated survey conducted in 2014+, 55.2% of fish oil users at baseline reported continuous intake of fish oil supplements (Spearman *r*=0.47) (Table S12).

Several potential confounders were also assessed through the touch-screen questionnaire, including age, sex, race, weight and height, education, TDI [3], household income, smoking, alcohol consumption, physical activity, history of various diseases, other dietary supplementation, medication records, and dietary intakes. BMI was calculated by dividing weight (kg) by height squared (m^2^). Metabolic equivalent of task (MET) was calculated according to the International Physical Activity Questionnaire short form [4]. Hypertension was defined as a self-reported history of hypertension, systolic blood pressure ≥140 mm Hg, diastolic blood pressure ≥90 mm Hg, or taking antihypertensive drugs. We created a healthy diet score ranging from 0 to 10 according to the recent definition of the ideal intake of dietary components for cardiometabolic health (Table S13) [5, 6].

**Ascertainment of T2D**

Definitions used to define prevalent and incident T2D cases are presented in Table S14. Prevalent T2D was ascertained using the UK Biobank algorithms for the diagnosis of diabetes [7]. Incident T2D cases were identified using cumulative hospital inpatient records with the ICD-10 code E11. Hospital admission data were available for participants until 31 March 2017. Detailed information on T2D ascertainment can be found at https://biobank.ctsu.ox.ac.uk/showcase/label.cgi?id52000. Follow-up duration was calculated from the data of attending the baseline assessment center to the time of T2D diagnosis, lost to follow-up, death, or the end of follow-up (31 March 2017), whichever occurred earlier.

**GRS calculation and interaction analysis**

The GRS calculation and interaction analysis with GRS and FA-associated SNP were mainly referred to our pervious study [8]. Specifically, an overall GRS for T2D was constructed based on 424 selected SNPs (Table S15) from the largest genome-wide multiethnic meta-analysis [9]. Then, we calculated the GRS through a widely used weighting method [10] using the following equation: GRS = β1 × SNP1+β2 ×SNP2+…+β424 × SNP424) × (424/sum of the β coefficients), in which SNPi is the number of risk allele of each SNP. Among the 358.4 to 465.2 calculated GRSs, the higher GRS was associated with a higher genetic predisposition to T2D [11]. We also obtained SNPs associated with circulating n-3 PUFAs from previous GWASs [12,13, 14] (Table S16) and SNPs at genes involved in glucose metabolism, including GLUT4, GYS1/2, GSK3β and PDPR [15‒20] (Table S11).

The potential interaction of fish oil use with GRS, SNPs related to circulating n-3 PUFAs levels or variants at genes involved in glucose metabolism for T2D was analyzed through multivariable Cox proportional hazards models including a multiplicative interaction term.

**Plasma metabolites**

Detailed descriptions of plasma metabolites measurements and quality control can be found at https://biobank.ctsu.ox.ac.uk/crystal/label.cgi?id=220. Briefly, about 120,000 plasma samples were randomly selected from the baseline collection (2006-2010) and plasma metabolites, including fatty acids, pyruvate, isoleucine, lactate and leucine, were measured by a high-throughput NMR-based metabolic biomarker profiling platform (Nightingale Health Ltd.).

**Animal study**

Four-week-old C57BL/KsJ-*db/db* mice were obtained from the Model Animal Research Center of Nanjing University (Nanjing, Jiangsu, China). The experimental design was approved by the Institutional Animal Care and Use Committee of Zhejiang University School of Medicine (Approval ID: ZSLL-2018-016). The animals were housed in a Specific Pathogen Free room at Zhejiang Chinese Medical University (Hangzhou, Zhejiang, China). A 12-hour light-dark cycle was maintained in the animal room with the temperature controlled at 23‒25 °C and humidity controlled at 55% ± 5%. The *db/db* mice were randomly divided into three diet groups and fed with a normal control diet (AIN93G; Research Diets, Inc., USA) (*db/db*), a diet enriched with 1% (w/w) of DHA (purity >99%; Larodan Fine Chemicals, Malmo, Sweden) (DHA), and a diet enriched with 1% (w/w) of EPA (purity >99%; Larodan Fine Chemicals) (EPA) for 10 weeks, respectively. Each group consisted of 6 male mice and 6 female mice, while all the animals were housed with free access to food and water. The composition and content of the experimental diets are shown in Table S17. At the end of the experiment, the mice were sacrificed and skeletal muscle was harvested for further analysis.

To explore the effects of DHA and EPA on insulin-dependent glucose uptake and oxidation, another 24 mice (*n*=4 male and *n*=4 female per group) were fasted for 4 h prior to receiving intraperitoneal injections of insulin (0.75 U/kg body weight) [21‒22]. Skeletal muscles were harvested from injected animals in each group after 15 min and then snap frozen.

**Cell study**

The mouse C2C12 myoblasts were purchased from Cell bank of Chinese Academy of Sciences (Shanghai, China) and cultured in a growth medium containing Dulbecco’s modified Eagle medium (DMEM, Corning, China) with high glucose, 10% fetal bovine serum (FBS, Gibco, USA), and 1% penicillin-streptomycin (Sigma-Aldrich, USA). When the cells grew to approximately 80% confluency, the addition of 10% FBS was replaced by 2% horse serum (HS, Gibco) and the cells were cultured for 4‒6 days until the myotubes formed. The insulin resistance model was induced by growth medium with 400 μM palmitic acid (Sigma-Aldrich, USA) after myotubes differentiated. The pure DHA and EPA (Nu-Chek, USA) were dissolved in ethanol at a concentration of 400 mM. Working solutions of DHA and EPA were made by diluting each stock solution in a pre-warmed differentiation medium containing 2% (w/v) fatty acid-free BSA (Sigma-Aldrich) to achieve a final concentration of 200 µM for each n-3 PUFA. Then, the C2C12 cells were treated with 200 μM DHA or EPA for 24 h, and the vehicle was used as the control.

**Metabolome analysis**

The skeletal muscle samples were weighed to a 1.5 mL EP tube with steel beads on ice, and homogenized with 500 μL 80% methanol. The mixture was centrifuged at 12,000 rpm for 10 min at 4°C. Then, the supernatant was filtered through a 0.22 μm hydrophilic microporous filter membrane, and transferred to a pre-opened sample vial for analysis.

For the UHPLC-Q-Orbitrap-HRMS analysis, chromatographic separation was carried out on a Dionex 3000 Ultimate UHPLC equipped with quadrupole-orbitrap mass spectrometer (Q-Exactive, Thermo Fisher Scientific, USA). Acquity UPLC HSS T3 column was chosen whose oven was held at 40 ℃, and a gradient elution program was used in the UHPLC analysis. High resolution mass spectrometry analysis was carried out with a heat electrospray ionization (HESI), while the parameters of HRMS were set as follows: Sheath Gas Flow Rate, 40L/min; Aux Gas Flow Rate, 10L/min; Spray Voltage, 3.5 kV in positive ion mode and 3 kV in negative ion mode; Capillary Temp., 350 °C. The mass scan ranged m/z 50‒750. Full MS/dd-MS2 (TopN) mode was used in quadrupole-orbitrap analysis under three stepped normalized collision energy including 20, 40 and 60 V. Data extraction was preformed using Xcalibur 4.1 software (Thermo Fisher Scientific, USA). The quality control (QC) sample was made by pooling of each extracted skeletal muscle samples. The Compound Discoverer 2.1 software (Thermo Fisher Scientific, USA) was used to preformed data analysis. According to the accurate mass of MS and tandem MS spectra, the structural formulas of metabolites were identified based on mzCloud (https://www.mzcloud.org) and Human Metabolome Database (HMDB) (http://www.hmdb.ca).

After normalization by weight of skeletal muscle samples, all the extracted ions from positive and negative ion modes by UHPLC-Q-Orbitrap-HRMS analysis were imported into SIMCA software (version 14.1, Umetrics, Sweden) for statistical analysis. A total of 3527 metabolites were detected and included in PCA and partial least squares discriminant analysis (OPLS-DA), in which a UV scaling process was applied. Variable contribution of the OPLS-DA model was ranked by the VIP. Metabolites whose VIP values exceeded 1 were considered as potential biomarkers which were evidently different between *db/db* group and DHA/EPA treatment group. The screened biomarkers were uploaded to MetaboAnalyst (https://www.metaboanalyst.ca/). All the biomarkers were uploaded to MetaboAnalyst (https://www.metaboanalyst.ca/) to identify perturbed pathways based on KEGG pathway database (https://www.genome.jp/kegg/).

For sensitivity analyses, according to results in PCA and heatmap, “outlier animals” including male mice numbered Con1, DHA1, and EPA3 and female mice numbered DHA2, DHA4, and EPA3 were excluded. Subsequently, the OPLS-DA models were rebuilt and biomarkers were selected according to VIP>1.

**Fatty acid content measurement**

To extract fatty acids, skeletal muscle tissues were weighed to a 10 mL glass tube and homogenized with 5 mL chloroform/methanol mixture for 1 min. After adding 1 mL 0.9% NaCl solution, the mixture was centrifuged at 2,000 rpm for 15 min. Then, the chloroform phase was collected and reacted with 10% sulfuric acid/methanol mixture in 65℃ for 30 min. Subsequently, fatty acids in skeletal muscle were extracted by isooctane. Extracted fatty acids were measured by Agilent 8890 chromatograph (Agilent Technologies, USA) coupled to Agilent 7000D mass spectrometer. An Agilent DB-23 column (60 m × 0.25 mm × 0.25 μm) was used. The GC conditions were as follows: the initial temperature was 100 ℃, which was kept for 1 min, and subsequently, the temperature was increased at 20 ℃ per min up to 175 ℃ and kept for 3 min, and then it was further increased at 3 ℃ per min to 190 ℃ and held for 3 min, and finally the temperature was increased at 0.5 ℃ per min up to 210 ℃ and held for 2 min. The injector temperature was 250 °C with 1 μL sample and the flow rate was 1.0 mL/min (carrier gas: helium). MS was operated in electron ionization (EI) mode (70 eV, 230 °C) with a solvent delay of 5 min. MS1 Scan of target ions was performed with 40‒400 m/z.

**GS and PDH measurement**

The skeletal muscle tissues were weighed to a 1.5 ml EP tube and homogenized with PBS for 1 min. The mixture was centrifuged at 12,000 rpm for 10 min at 4°C, and the supernatant was then collected for further measurement. The contents of GS and PDH were measured using ELISA kits (MYBioSource, MBS166026 and MBS269561) according to the manufacturer’s instruction.

**Potential biomarkers measurement**

The contents of pyruvic acid, lactic acid, and total BCAAs in skeletal muscle after insulin injection was measured using corresponding assay kits (Solarbio, BC2205; Solarbio, BC2235; COIBO BIO, CB13837-Mu) according to the manufacturer’s instruction.

**PDH and CS activities analysis**

The activities of PDH and CS in skeletal muscle after insulin injection were measured using PDH activity assay kit (Solarbio, BC0385) and CS activity assay kit (Solarbio, BC1065) according to the manufacturer’s instruction.

**Glucose consumption assay**

DHA and EPA were delivered to differentiated C2C12 myotubes. After 24 h of fatty acid intervention, these myotubes were incubated with 100 nM insulin (Solarbio, I8830) for 30 min, and glucose concentration was further measured by an assay kit (Applygen, E1010) using the glucose oxidase method. The glucose consumption is the primary glucose concentration in differentiation medium minus the terminal glucose concentration in cell culture supernatants.

**Cell transfection**

HA-Glut4-GFP plasmids was a gift from Dr. Yingke Xu. For each construct, several clones were chosen with 50 μM ampicillin and performed PCR program with GLUT4 primers (Forward: 5′-GCAGATCGGCTCTGACGATG-3′, Reverse: 5′- CAATCACCTTCTGTGGGGCA-3′) to confirm their identity. All plasmids were purified using a miniprep kit (TianGen, China) for use in transfection experiments. The Escherichia coli strain DH5α was used as a host for the propagation of the clones. The C2C12 cells were electrically transfected with using a Nucleofector kit (Lonza, V4XC) according to the manufacturer’s instruction.

**Cell-surface HA-GLUT4-GFP detection**

HA-GLUT4-GFP at cell surface was detected by immunofluorescence in C2C12 myoblasts seeded on 15-mm confocal culture dishes. Subsequently, myoblasts were intervened with 200 nM DHA or EPA for 24 h, and then treated with 100 nM insulin for 30 min. Cells were then rinsed with PBS, fixed with 4% paraformaldehyde (PFA), and blocked with a blocking solution containing 5% skimmed milk powder for 1 h. HA-GLUT4-GFP at cell surface was detected by anti-HA (Affinity Biosciences, T0008) overnight. After three washes with PBS, cells were incubated with DyLight 594 conjugated anti-mouse secondary antibody (EarthOx Life Sciences, E032410) in the dark for 1 h, and rinsed with PBS for 3 times. Fluorescence images were acquired with an OLYMPUS IX83-FV3000-OSR high-resolution confocal microscopy (Olympus, Japan).

**Real-time PCR**

The related mRNA expressions in skeletal muscle and C2C12 myotubes were detected by RT-PCR. Total RNA was extracted using Trizol (Takara, 9108). The inverse transcription of RNA was performed using the RT reagent kit (Takara, RR047A). The levels of mRNA were quantified with the LightCycler480 (Roche, Germany) using a two-step quantitative RT-PCR method [23]. Then, the cycle threshold values were calculated using the LightCycler480 Software (Roche, Germany). The GAPDH gene was used as an internal control to quantify the expression of the related maker genes and all primers were synthesized by Tsingke Biotechnology (Beijing, China). The primers used are shown in Table S18.

**Western blot analysis**

Total protein was extracted from skeletal muscle or C2C12 myotubes using RIPA lysis buffer (Meilunbio, MA0151) containing 1 mM protease inhibitors (Meilunbio, MA0151). For cell membrane protein extraction, the membrane protein extraction kit (Beyotime, P0033) was used according to the manufacturer’s instruction. The protein samples mixed with loading buffer were separated by SDS PAGE and transferred onto a PVDF membrane (Millipore, IPVH00010). The protein-carried PVDF membrane was blocked with 5% BSA. Western blotting was carried out using corresponding detection antibodies and reacted with ECL reagent (Meilunbio, MA0186). The detailed information of antibodies used in this study are listed in Table S19.

**Statistical Analysis**

For human study, we used Cox proportional hazards regression models to calculate hazard ratios (HRs) and 95% confidence intervals (CIs) for T2D according to fish oil use by comparing users with non-users after checking the violation of the proportional hazard assumption. Several potential confounders were included in our multivariable-adjusted models. Model 1 was adjusted for age and sex. Model 2 was further adjusted for race, centers, BMI, education, TDI, household income, smoking, alcohol consumption, physical activity, history of hypertension, history of high cholesterol, and family history of diabetes. Model 3 was additionally adjusted for vitamin supplement use, mineral supplement use, glucosamine use, and aspirin use. Model 4 was additionally adjusted for the consumption of oily fish, vegetables, and fruits. The final model 5 was further adjusted for a healthy diet score based on model 3. Missing data were coded as a missing indicator category if necessary. Subgroup analysis by sex was then performed to detect sex differences. A general linear model was used to assess the association of fish oil supplementation with baseline levels of HbA1c and CRP. Multivariable-adjusted general linear models were used to evaluate the relationships of fish oil use with baseline plasma levels of n-3 PUFAs and also tested whether relationships were modified by n-3 PUFAs-associated loci. We also created an overall healthy lifestyle score based on BMI (<30 kg/m^2^), smoking (never), physical activity (≥600 MET min/week), and healthy diet (yes) [24]. Several sensitivity analyses were conducted to test the robustness of our findings. We tested whether the association was affected by further adjusting for HbA1c, CRP, extreme BMIs (BMI <18.5 kg/m^2^ or BMI >40 kg/m^2^), antihypertensive agents, or hormone replacement therapy and oral contraceptive use. We also performed the sensitivity analyses by further excluding incident T2D cases that occurred within 2 years to minimize the possibility of reverse causation, or participants who took any other supplements to rule out the influence of other supplements. Finally, analyses were restricted to participants with no missing covariate data. We also used multivariable-adjusted general linear models to evaluate the relationships of baseline plasma levels of n-3 PUFAs with pyruvate, isoleucine, lactate and leucine. Statistical analyses were performed with the SAS 9.4 software (SAS Institute, Cary, NC, USA). A two-tailed *P*<0.05 was considered to be statistically significant.

For intervention experiments in mice and C2C12 myotubes, data were represented as mean ± standard error of the mean (SEM). Differences among groups were analyzed by two-tailed Student's *t*-test for two groups, and one-way analysis of variance (ANOVA) for more than two groups using Graphpad Prism 8.0. Post hoc comparisons were performed using either the Tukey test (for balanced group designs) or the Tukey-Kramer test (for unbalanced designs), with selection contingent upon sample size homogeneity. *P*<0.05 was considered statistically significant.

References：

1. Palmer, L. J. UK Biobank: bank on it. *Lancet*. 2007;369(9578):1980-1982.

2. Sudlow, C., Gallacher, J., Allen, N., Beral, V., Burton, P., Danesh, J., Downey, P., Elliott, P., Green, J., Landray, M. *et al.* UK biobank: an open access resource for identifying the causes of a wide range of complex diseases of middle and old age. *PLoS. Med.* 2015;12(3):e1001779.

3. Townsend, P. Deprivation. *J. Soc. Policy.* 1987;16:125-146.

4. Organization, W. H. Global recommendations on physical activity for health. (World Health Organization, Geneva, Switzerland, 2010).

5. Mozaffarian, D. Dietary and policy priorities for cardiovascular disease, diabetes, and obesity. *Circulation*. 2016;133(2):187-225.

6. Said, M. A., Verweij, N., van der Harst, P. Associations of combined genetic and lifestyle risks with incident cardiovascular disease and diabetes in the UK Biobank Study. *JAMA. Cardiol.* 2018;3(8):693-702.

7. Eastwood, S. V., Mathur, R., Atkinson, M., Brophy, S., Sudlow, C., Flaig, R., de Lusignan, S., Allen, N., Chaturvedi, N. Algorithms for the capture and adjudication of prevalent and incident diabetes in UK Biobank. *PLoS One.* 2016;11(9):e0162388.

8. Zhuang, P., Liu, X., Li, Y., Li, H., Zhang, L., Wan, X., Wu, Y., Zhang, Y., Jiao, J. Circulating fatty acids and genetic predisposition to type 2 diabetes: gene-nutrient interaction analysis. *Diabetes Care*. 2022;45(3):564-575.

9. Vujkovic, M., Keaton, J. M., Lynch, J. A., Miller, D. R., Zhou, J., Tcheandjieu, C., Huffman, J. E., Assimes, T. L., Lorenz, K., Zhu, X. *et al.* Discovery of 318 new risk loci for type 2 diabetes and related vascular outcomes among 1.4 million participants in a multi-ancestry meta-analysis. *Nat. Genet.* 2020;52(7):680-691.

10. Huang, T., Qi, Q., Zheng, Y., Ley, S. H., Manson, J. E., Hu, F. B., Qi, L. Genetic predisposition to central obesity and risk of type 2 diabetes: two independent cohort studies. *Diabetes Care*. 2015;38(7):1306-1311.

11. Udler, M. S., Kim, J., von Grotthuss, M., Bonas-Guarch, S., Cole, J. B., Chiou, J., Christopher, D. A. o. b. o. M., the, I., Boehnke, M., Laakso, M. *et al.* Type 2 diabetes genetic loci informed by multi-trait associations point to disease mechanisms and subtypes: A soft clustering analysis. *PLoS. Med.* 2018;15(9):e1002654.

12. Lemaitre, R. N., Tanaka, T., Tang, W., Manichaikul, A., Foy, M., Kabagambe, E. K., Nettleton, J. A., King, I. B., Weng, L. C., Bhattacharya, S. *et al.* Genetic loci associated with plasma phospholipid n-3 fatty acids: a meta-analysis of genome-wide association studies from the CHARGE Consortium. *PLoS genetics.* 2011;7(7): e1002193.

13. Draisma, H. H. M., Pool, R., Kobl, M., Jansen, R., Petersen, A. K., Vaarhorst, A. A. M., Yet, I., Haller, T., Demirkan, A., Esko, T. *et al.* Genome-wide association study identifies novel genetic variants contributing to variation in blood metabolite levels. *Nat. Commun.* 2015;6:7208.

14. Shin, S. Y., Fauman, E. B., Petersen, A. K., Krumsiek, J., Santos, R., Huang, J., Arnold, M., Erte, I., Forgetta, V., Yang, T. P. *et al.* An atlas of genetic influences on human blood metabolites. *Nat. Genet.* 2014;46(6):543-550.

15. Yu, F., Liu, F., Li, X. M., Zhao, Q., Luo, J. Y., Zhang, J. Y., Yang, Y. N. GLUT4 gene rs5418 polymorphism is associated with increased coronary heart disease risk in a Uygur Chinese population. *Bmc. Cardiovasc. Disor.* 2022;22(1):191.

16. Malodobra-Mazur, M., Bednarska-Chabowska, D., Olewinski, R., Chmielecki, Z., Adamiec, R., Dobosz, T. Single nucleotide polymorphisms in 5'-UTR of the SLC2A4 gene regulate solute carrier family 2 member 4 gene expression in visceral adipose tissue. *Gene*. 2016;576(1 Pt 3):499-504.

17. Yang, L., Sun, Z., Li, J., Pan, X., Wen, J., Yang, J., Wang, Q., Chen, P. Genetic variants of glycogen metabolism genes were associated with liver PDFF without increasing NAFLD risk. *Front. Genet.* 2022;13:830445.

18. Lin, Y. F., Huang, M. C., Liu, H. C. Glycogen synthase kinase 3β gene polymorphisms may be associated with bipolar I disorder and the therapeutic response to lithium. *J. Affect. Disorders.* 2013;147(1-3):401-406.

19. Shim, S. H., Hwangbo, Y., Kwon, Y. J., Lee, H. Y., Kim, J. H., Yoon, H. K., Hwang, J. A., Kim, Y. K. Association between glycogen synthase kinase-3β gene polymorphisms and attention deficit hyperactivity disorder in Korean children: a preliminary study. *Prog. Neuro-Psychoph.* 2012;39(1):57-61.

20. Riveros-Mckay, F., Oliver-Williams, C., Karthikeyan, S., Walter, K., Kundu, K., Ouwehand, W. H., Roberts, D., Di Angelantonio, E., Soranzo, N., Danesh, J. *et al.* The influence of rare variants in circulating metabolic biomarkers. *PLoS genetics*. 2020;16(3):e1008605.

21. Hausen, A.C., Ruud, J., Jiang, H., Hess, S., Varbanov, H., Kloppenburg, P., Bruning, J.C. Insulin-dependent activation of MCH neurons impairs locomotor activity and insulin sensitivity in obesity. *Cell Rep.* 2016;17(10):2512-2521.

22. Gonzalez-Rodriguez, A., Mas Gutierrez, J.A., Sanz-Gonzalez, S., Ros, M., Burks, D.J., Valverde, A.M. Inhibition of PTP1B restores IRS1-mediated hepatic insulin signaling in IRS2-deficient mice. *Diabetes.* 2010;59(3):588-599.

23. Bustin, S. A., Benes, V., Garson, J. A., Hellemans, J., Huggett, J., Kubista, M., Mueller, R., Nolan, T., Pfaffl, M. W., Shipley, G. L. The MIQE guidelines: minimum information for publication of quantitative real-time PCR experiments. *Clin. Chem.* 2009;55(4):611-622*.*

24. Ma, H., Li, X., Zhou, T., Sun, D., Liang, Z., Li, Y., Heianza, Y., Qi, L. Glucosamine use, inflammation, and genetic susceptibility, and incidence of type 2 diabetes: a prospective study in UK Biobank. *Diabetes Care*. 2020;43(4):719-725.


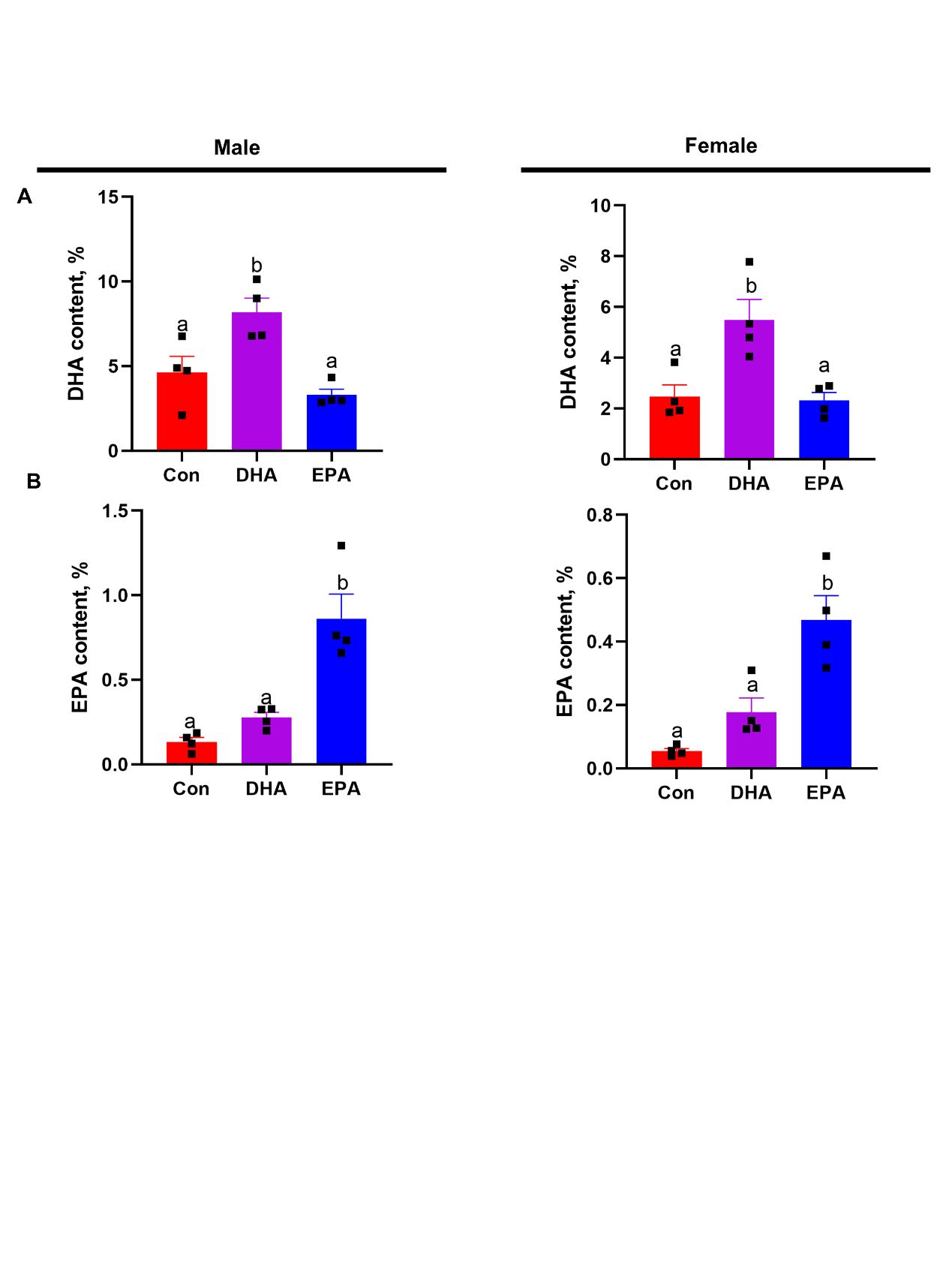


**Figure S1. DHA and EPA contents of skeletal muscles in male and female *db/db* mice.** (**A**) DHA contents of skeletal muscles in male and female *db/db* mice. (**B**) EPA contents of skeletal muscles in male and female *db/db* mice. *n=*4 for all groups. Data are presented as means ± SEM. Statistical differences are determined by One-way ANOVA followed by the Tukey's multiple comparisons test. Groups with different superscript letters are significantly different (*P*<0.05).

**
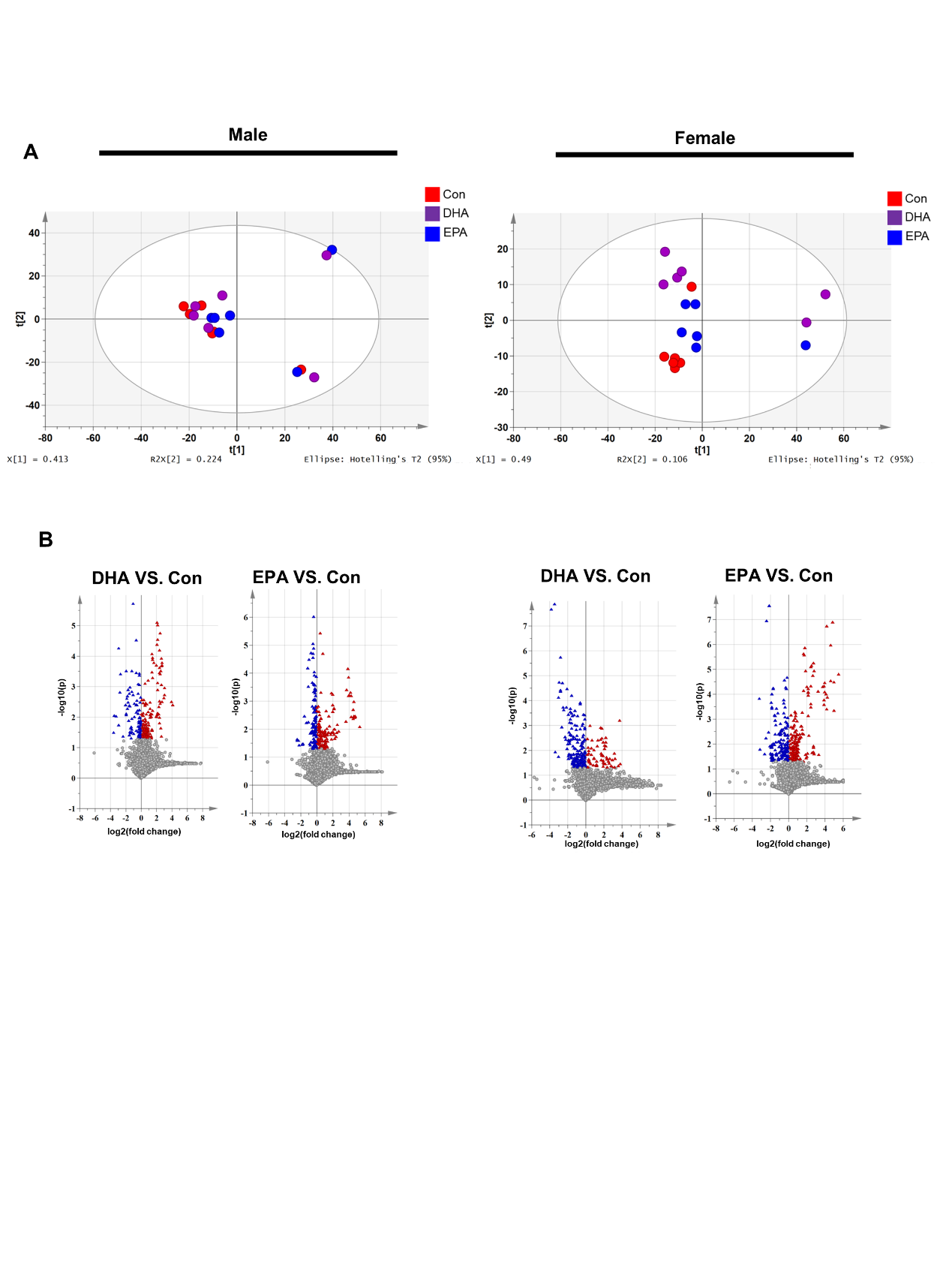
**

**Figure S2. Metabolome analysis of skeletal muscle in male and female *db/db* mice.** (**A**) PCA analysis of muscular metabolome in male and female *db/db* mice. (**B**) Volcano plots of metabolites (*P*<0.05) which significantly changed in DHA or EPA fed male and female *db/db* mice. Blue points represent metabolites significantly down-regulated by DHA/EPA; red points represent metabolites significantly up-regulated by DHA/EPA.

**
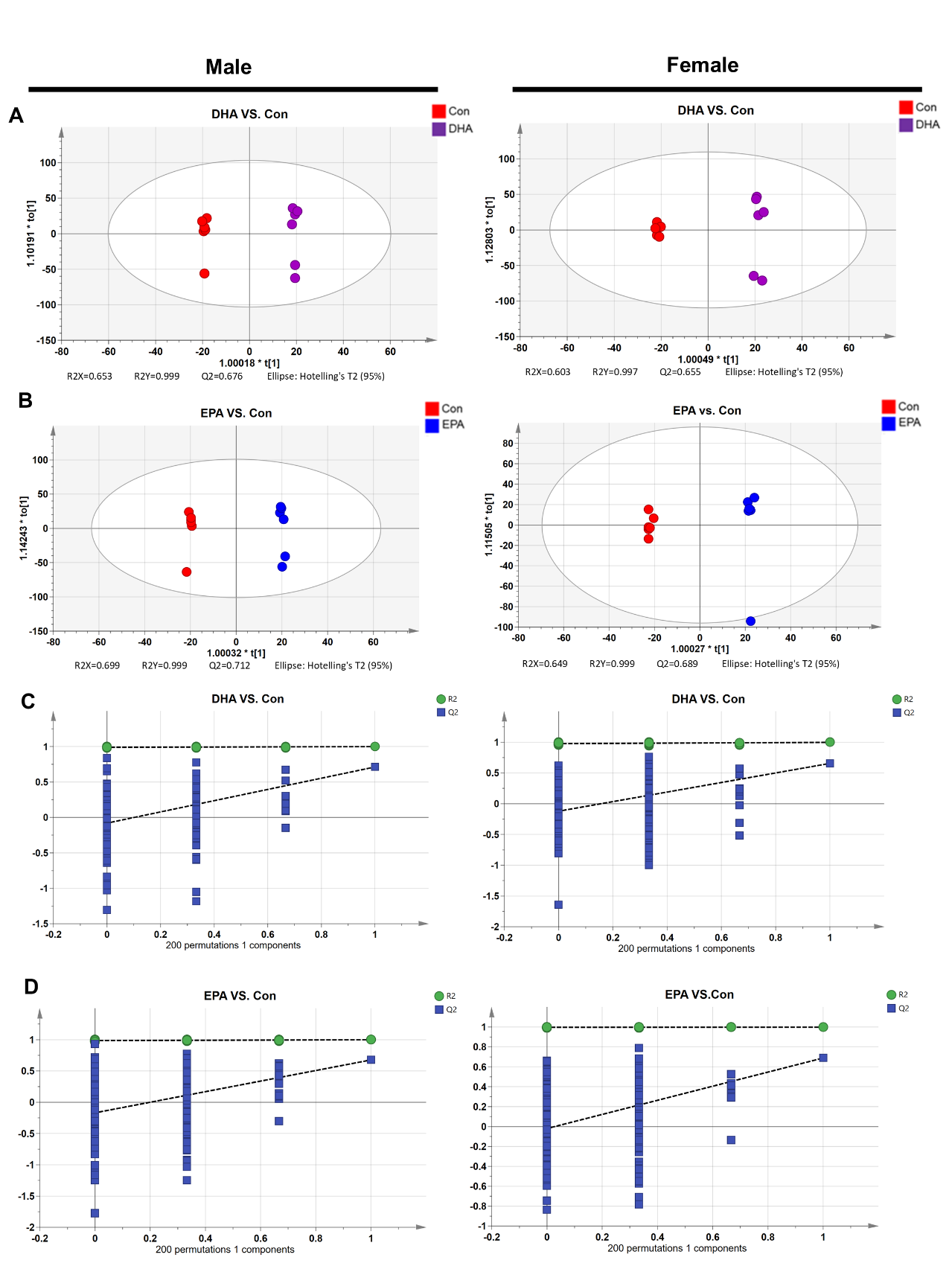
**

**Figure S3. OPLS‒DA analysis of muscular metabolome in male and female *db/db* mice.** (**A**) OPLS‒DA analysis between DHA treatment group and the control group (Male-DHA: *R*^2^X=0.699, *R*^2^Y=0.99, *Q*^2^=0.712; Female-DHA: *R*^2^X=0.603, *R*^2^Y=0.997, *Q*^2^=0.655). (**B**) OPLS-DA analysis between EPA treatment group and the control group (Male‒EPA: *R*^2^X=0.653, *R*^2^Y=0.999, *Q*^2^=0.676; Female‒EPA: *R*^2^X=0.649, *R*^2^Y=0.999, *Q*^2^=0.689). (**C**) 200-time permutation test in OPLS‒DA model between DHA treatment group and the control group. (**D**) 200-time permutation test in OPLS-DA model between EPA treatment group and the control group. *n=*6 for all groups.

**
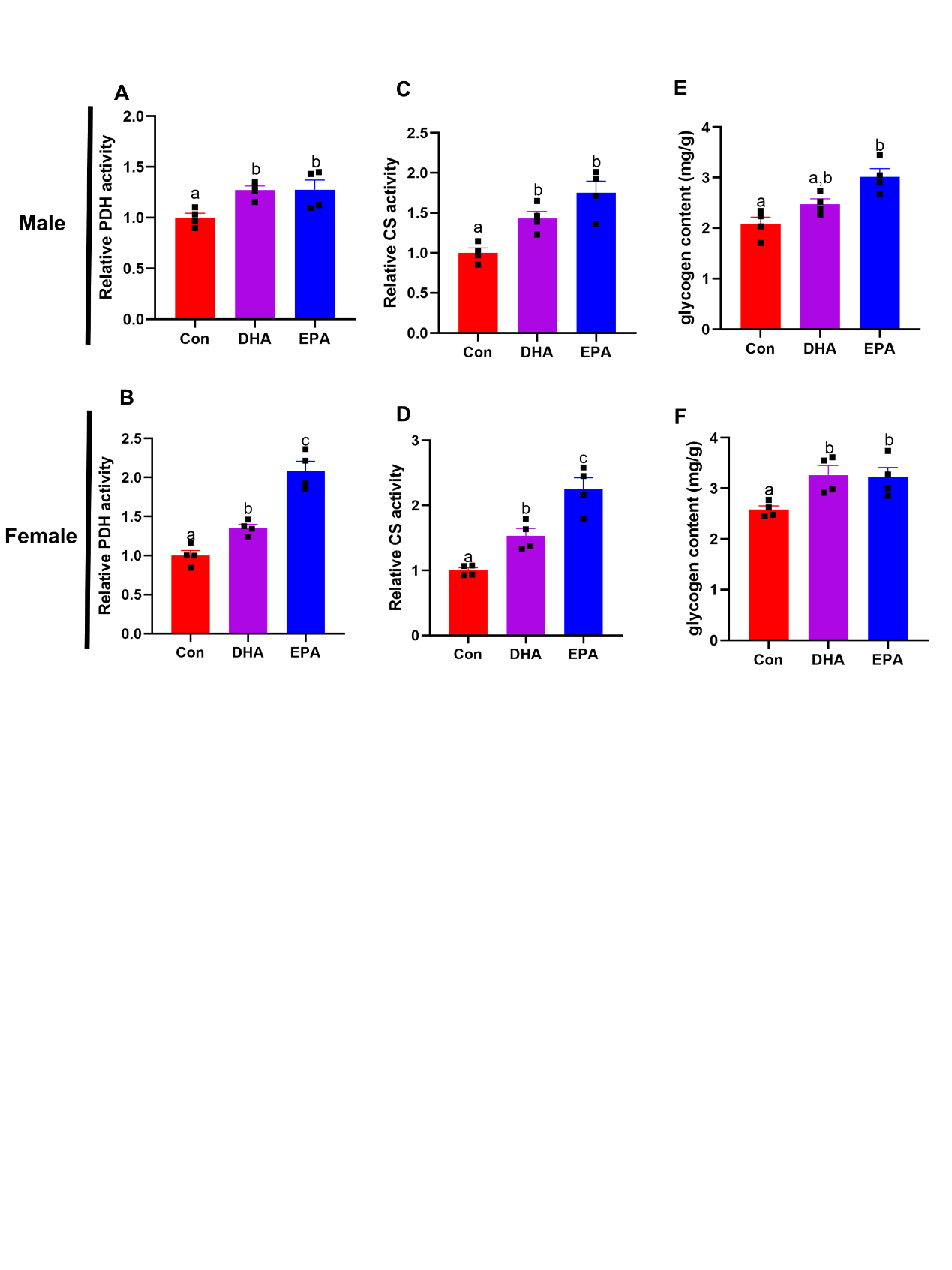
**

**Figure S4. Effects of DHA and EPA on glycogen synthesis and glucose oxidation in skeletal muscles under insulin stimulation.** (**A-B**) PDH activity. (**C-D**) CS activity. (**E-F**) The content of glycogen. *n=*4 for all groups. Data are presented as means ± SEM. Statistical differences were determined by One-way ANOVA followed by the Tukey's multiple comparisons test. Groups with different superscript letters are significantly different (*P*<0.05).

**
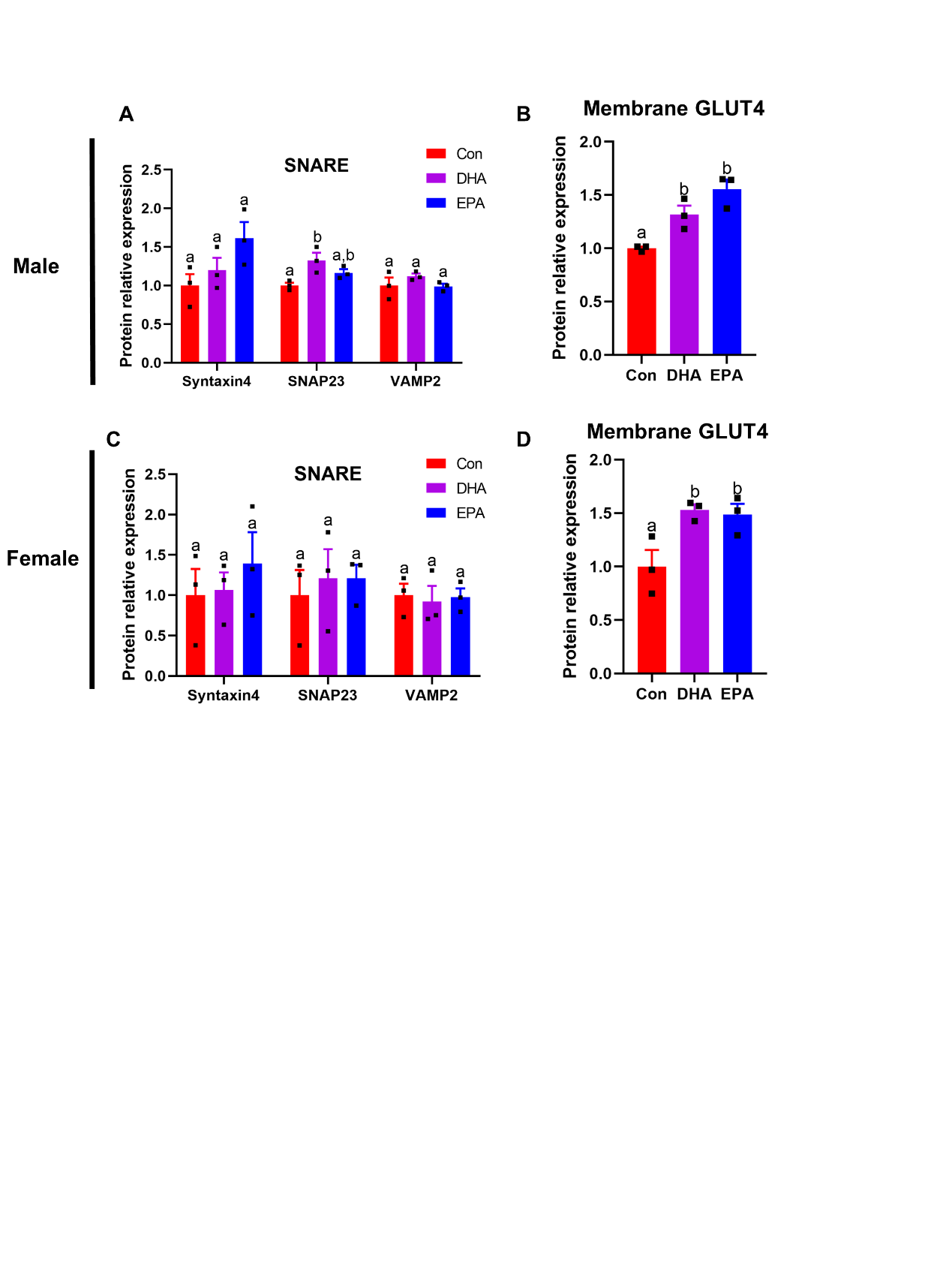
**

**Figure S5. Quantitative results of Western blotting analysis in *db/db* mice.** (**A**) The SNARE proteins expression in male mice. (**B**) The membrane GLUT4 expression under insulin stimulation in male mice. (**C**) The SNARE proteins expression in female mice. (**D**) The membrane GLUT4 expression under insulin stimulation in female mice. *n*=3 for all groups. Data are presented as means ± SEM. Statistical differences were determined by One-way ANOVA followed by the Tukey's multiple comparisons test. Groups with different superscript letters are significantly different (*P*<0.05).

**
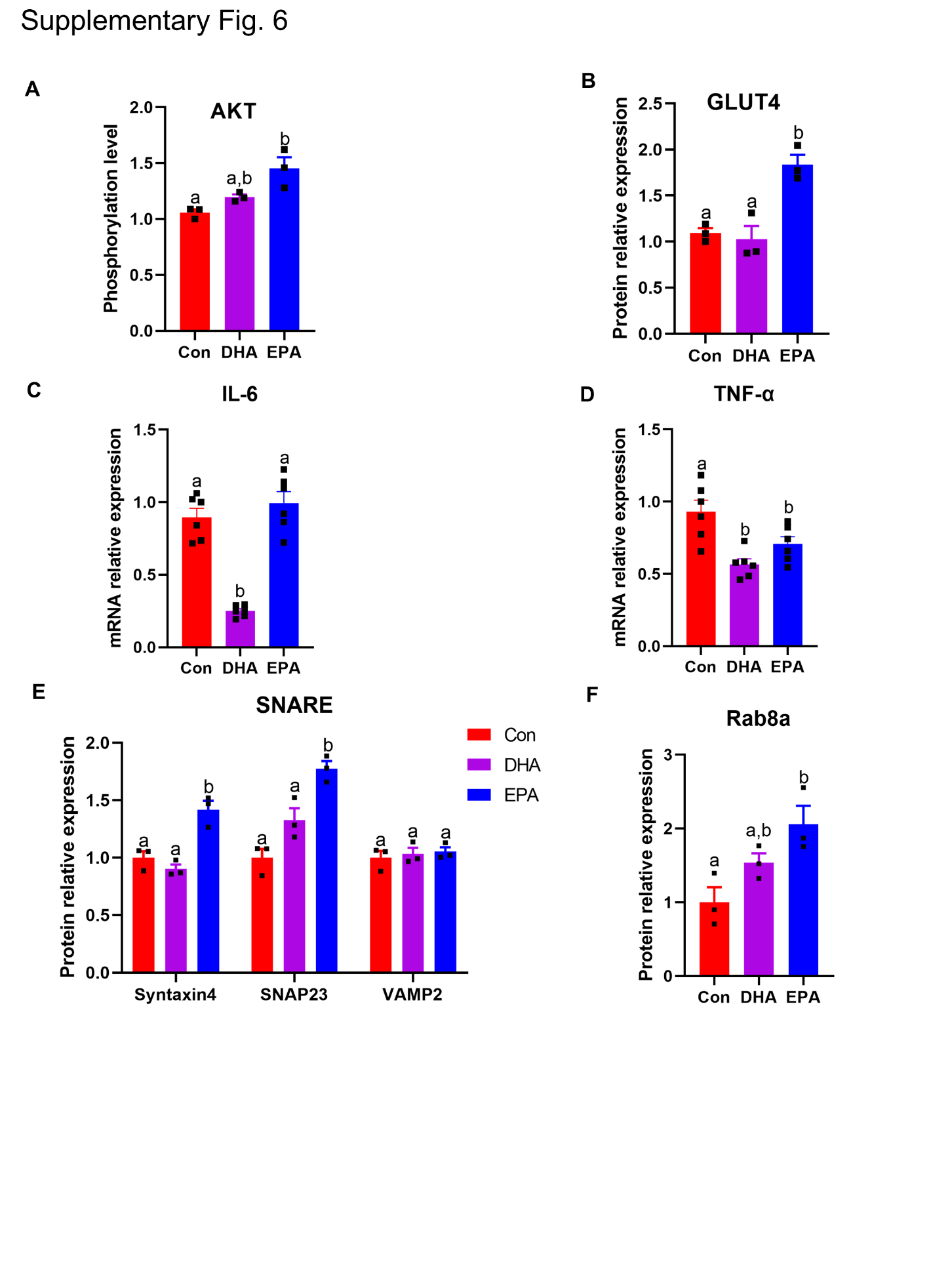
**

**Figure S6. The expression of markers in insulin signaling and inflammation in insulin-resistant C2C12 cells.** (**A-B**) Quantitative results of immunoblots on AKT phosphorylation and GLUT4. (**C-D**) qPCR analysis for inflammation factors. (**E-F**) Quantitative results of immunoblots on SNAREs and Rab8a. For (**A-B**, **E-F**) *n=*3 for all groups. For (**C-D**) *n=*6 for all groups. Data are presented as means ± SEM. Statistical differences were determined by One-way ANOVA followed by the Tukey's multiple comparisons test. Groups with different superscript letters are significantly different (*P*<0.05).

**
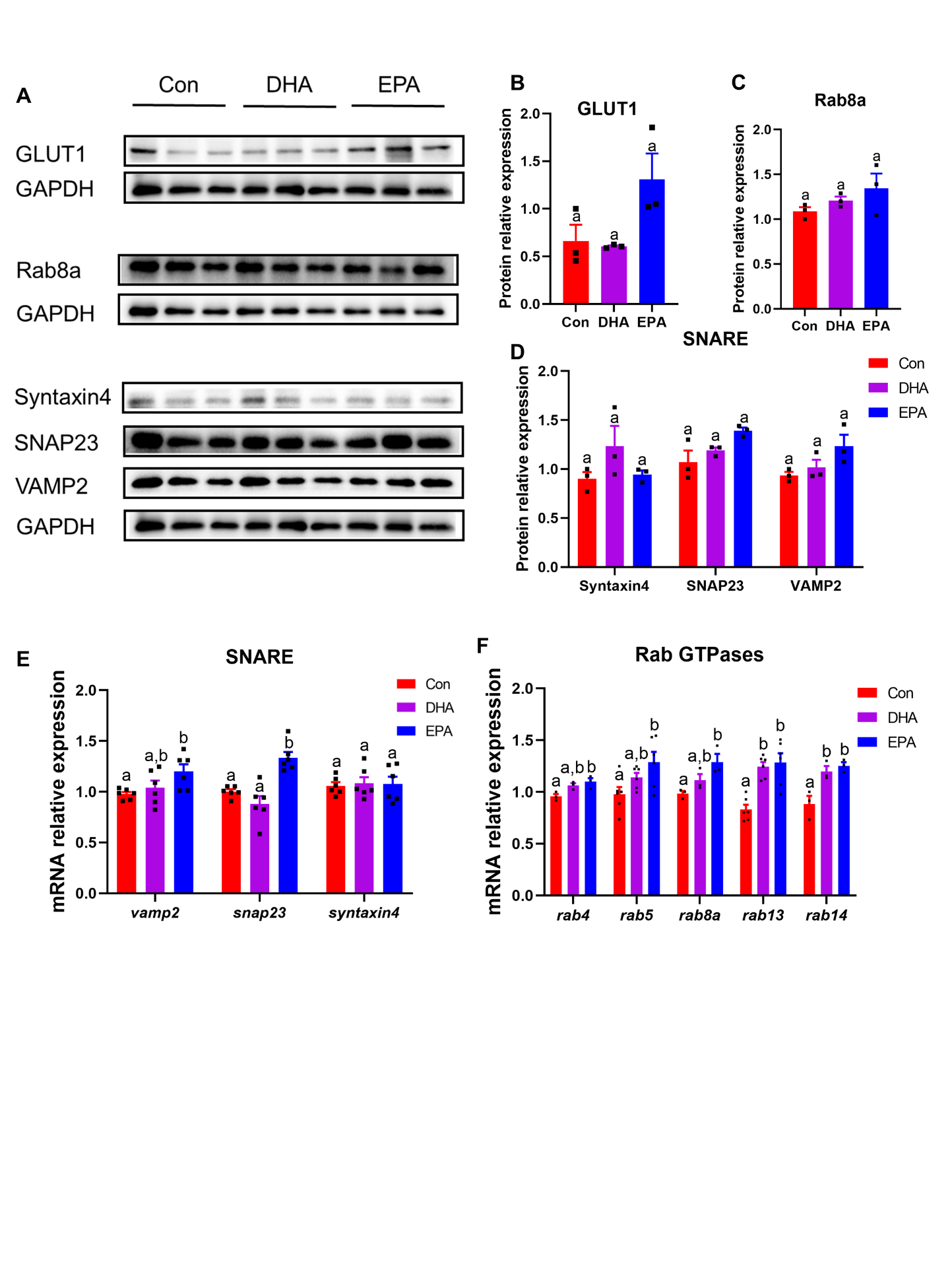
**

**Figure S7. DHA and EPA regulate GLUT4 translocation in insulin-resistant C2C12 cells without insulin stimulation.** (**A**) Representative immunoblots for GLUT1, SNAREs, and Rab8a in insulin-resistant myotubes. (**B‒D**) Quantitative results of Western blotting analysis. (**E‒F**) qPCR analysis for SNARE genes and Rab GTPases in basal state. *n=*3 for all groups. Data are presented as means ± SEM. Statistical differences were determined by One-way ANOVA followed by the Tukey's multiple comparisons test. Groups with different superscript letters are significantly different (*P*<0.05).

**
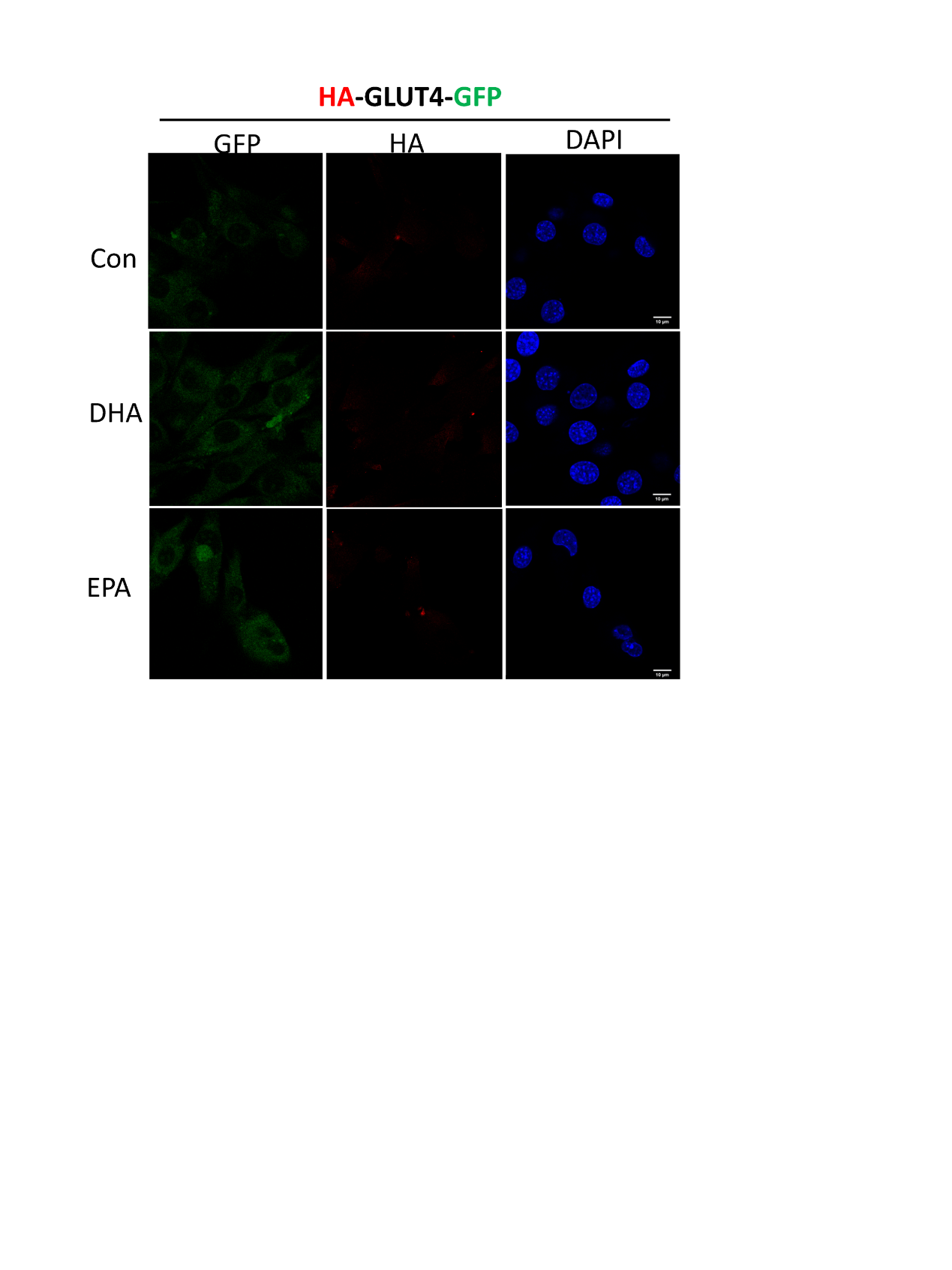
**

**Figure S8. Immunofluorescence of GLUT4 in C2C12 cells.** Immunofluorescence of total GLUT4 (green) and plasma membrane GLUT4 (red) in C2C12 cells under basal state.

**
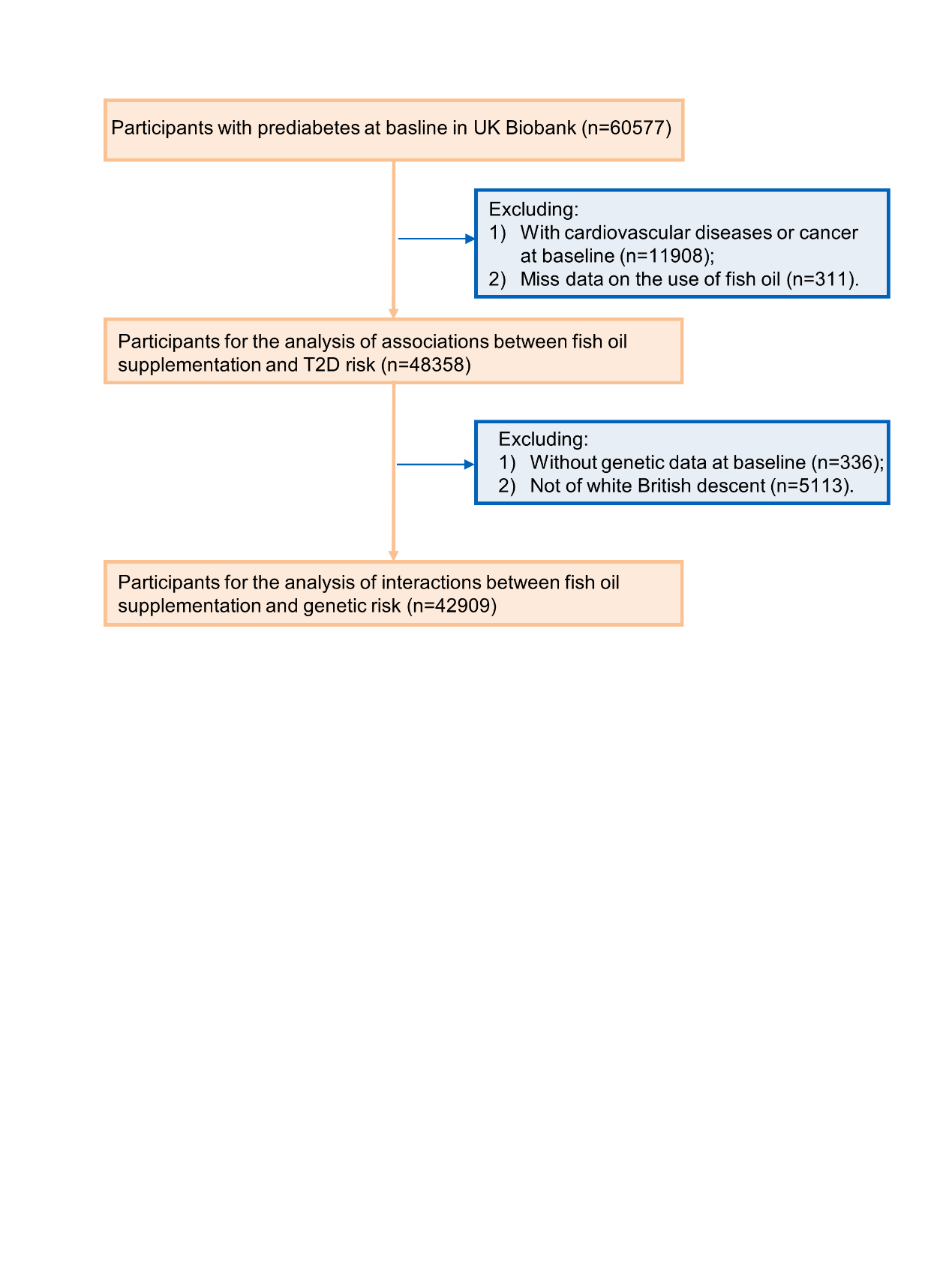
**

**Figure S9. Flow of participants in the current UK biobank study.**

| **Table S1. HR (95% CI) of type 2 diabetes according to fish oil use in the UK Biobank in prediabetes patients** | | | |
| --- | --- | --- | --- |
|  | **Fish oil non-users** | **Fish oil users** |  |
|  | **(*n*=32178)** | **(*n*=16180)** | ***P* value** |
| No of cases (%) | 2365 (7.4) | 1020 (6.3) |  |
| Person-years | 251201 | 127236 |  |
| Model 1 | 1 [Reference] | 0.85 (0.79‒0.92) | <0.001 |
| Model 2 | 1 [Reference] | 0.91 (0.85‒0.98) | 0.016 |
| Model 3 | 1 [Reference] | 0.90 (0.83‒0.98) | 0.015 |
| Model 4 | 1 [Reference] | 0.91 (0.84‒0.99) | 0.028 |
| Model 5 | 1 [Reference] | 0.91 (0.84‒0.99) | 0.021 |
| CI = confidence interval; HR = hazard ratio. | | | |
| **Model 1**: Results were adjusted for age and sex. | | | |
| **Model 2**: model 1 + race (white or not), centers (22 centers), BMI (<18.5, 18.5‒25.0, 25.0‒30.0, >30.0 kg/m^2^, or missing), education (college or university degree, vocational qualifications, optional national exams at ages 17–18 years, national exams at age 16 years, others, or missing), Townsend deprivation index (quintiles), household income (<£18,000, £18,000‒£30,999, £31,000‒£51,999, £52,000‒£100,000, >£100,000, or missing), smoking (never, former, current, or missing), alcohol consumption (<1, 1 or 2, 3 or 4, ≥5 times/week, or missing), physical activity (<150 or ≥150 min/week), history of hypertension (yes or no), history of high cholesterol (yes or no), family history of diabetes (yes or no). | | | |
| **Model 3**: model 2 + vitamin supplement use (yes or no), mineral supplement use (yes or no), glucosamine use (yes or no), aspirin use (yes or no). | | | |
| **Model 4:** model 3 + oily fish (<1, 1, or ≥2 times/week), vegetables (<1.0, 1.0‒2.9, or ≥3.0 servings/day), fruits (<2.0, 2.0‒3.9, or ≥4.0 servings/day). | | | |
| **Model 5**: model 3 + healthy diet score. | | | |

| **Table S2. HR (95% CI) of type 2 diabetes according to fish oil use in men and women with prediabetes** | | | | |
| --- | --- | --- | --- | --- |
| **Subgroup** | **No of participants** | **Fish oil non-users** | **Fish oil users** | **P value for interaction** |
| **Sex** |  |  |  | 0.005 |
| Women | 27071 | 1.00 | 0.84 (0.75-0.95) |  |
| Men | 21287 | 1.00 | 0.98 (0.88-1.10) |  |
| The multivariate model was adjusted for age, sex, race, centers, BMI, education, Townsend deprivation index, household income, smoking, alcohol consumption, physical activity, history of hypertension, history of high cholesterol, family history of diabetes, vitamin supplement use, mineral supplement use, glucosamine use, aspirin use, and healthy diet score. | | | | |

| **Table S3. HR (95% CI) of type 2 diabetes in prediabetes in sensitivity analyses** | | |  |
| --- | --- | --- | --- |
|  | **Fish oil non-users** | **Fish oil users** | ***P* value** |
| **Further adjusted for HbA1c** |  |  |  |
| No. of cases (%) | 2365 (7.4) | 1020 (6.3) |  |
| Age- and sex-adjusted HR (95% CI) | 1 [Reference] | 0.88 (0.81-0.95) | <0.001 |
| Multivariable-adjusted HR (95% CI) | 1 [Reference] | 0.90 (0.83-0.98) | 0.012 |
| **Further adjusted for CRP** |  |  |  |
| No. of cases (%) | 2251 (7.4) | 974 (6.3) |  |
| Age- and sex-adjusted HR (95% CI) | 1 [Reference] | 0.87 (0.81-0.94) | <0.001 |
| Multivariable-adjusted HR (95% CI) | 1 [Reference] | 0.91 (0.84-1.00) | 0.037 |
| **Further adjusted for lipid-lowering drug** | |  |  |
| No. of cases (%) | 2365 (7.4) | 1020 (6.3) |  |
| Age- and sex-adjusted HR (95% CI) | 1 [Reference] | 0.85 (0.79-0.91) | <0.001 |
| Multivariable-adjusted HR (95% CI) | 1 [Reference] | 0.91 (0.84-0.99) | 0.023 |
| **Further adjusted for antihypertensive agents** | |  |  |
| No. of cases (%) | 2365 (7.4) | 1020 (6.3) |  |
| Age- and sex-adjusted HR (95% CI) | 1 [Reference] | 0.86 (0.80-0.92) | <0.001 |
| Multivariable-adjusted HR (95% CI) | 1 [Reference] | 0.91 (0.84-0.99) | 0.025 |
| **Further adjusted for hormone replacement therapy and oral contraceptive use** | | | |
| No. of cases (%) | 2365 (7.4) | 1020 (6.3) |  |
| Age- and sex-adjusted HR (95% CI) | 1 [Reference] | 0.85 (0.79-0.91) | <0.001 |
| Multivariable-adjusted HR (95% CI) | 1 [Reference] | 0.91 (0.84-0.99) | 0.021 |
| **Excluding incident T2D cases within 2 years** | |  |  |
| No. of cases (%) | 2197 (6.9) | 951 (5.9) |  |
| Age- and sex-adjusted HR (95% CI) | 1 [Reference] | 0.85 (0.79-0.92) | <0.001 |
| Multivariable-adjusted HR (95% CI) | 1 [Reference] | 0.90 (0.83-0.99) | 0.022 |
| **Excluding participants with extreme BMI** | |  |  |
| No. of cases (%) | 2105 (6.9) | 949 (6.1) |  |
| Age- and sex-adjusted HR (95% CI) | 1 [Reference] | 0.87 (0.81-0.94) | <0.001 |
| Multivariable-adjusted HR (95% CI) | 1 [Reference] | 0.92 (0.84-1.00) | 0.055 |
| **Excluding participants who took any other supplements** | | |  |
| No. of cases (%) | 1722 (7.5) | 301 (6.9) |  |
| Age- and sex-adjusted HR (95% CI) | 1 [Reference] | 0.90 (0.80-1.02) | 0.101 |
| Multivariable-adjusted HR (95% CI) | 1 [Reference] | 0.95 (0.84-1.07) | 0.382 |
| **Excluding participants with missing covariate data** | |  |  |
| No. of cases (%) | 1474 (6.9) | 668 (6.21) |  |
| Age- and sex-adjusted HR (95% CI) | 1 [Reference] | 0.89 (0.81-0.98) | 0.017 |
| Multivariable-adjusted HR (95% CI) | 1 [Reference] | 0.93 (0.84-1.04) | 0.192 |
| CI = confidence interval; HR = hazard ratio. | | | |
| The multivariate model was adjusted for age, sex, race, centers, BMI, education, Townsend deprivation index, household income, smoking, alcohol consumption, physical activity, history of hypertension, history of high cholesterol, family history of diabetes, vitamin supplement use, mineral supplement use, glucosamine use, aspirin use, and healthy diet score. | | | |

| **Table S4 Interactions of fish oil with GRS for T2D** | | | | | |  |  |  |  |  |  |
| --- | --- | --- | --- | --- | --- | --- | --- | --- | --- | --- | --- |
|  | **GRS (*n* Loci = 424)** | | | | | | | | | | |
|  | **Fish oil** | | |  | **GRS** | | |  | **Fish oil × GRS** | | |
|  | **β** | **SE** | ***P*** |  | **β** | **SE** | ***P*** |  | **β** | **SE** | ***P*** |
| T2D | -0.098 | 0.046 | **0.033** |  | 0.223 | 0.022 | **<0.001** |  | 0.019 | 0.041 | 0.633 |
| The multivariate model was adjusted for age, sex, race, centers, BMI, education, Townsend deprivation index, household income, smoking, alcohol consumption, physical activity, history of hypertension, history of high cholesterol, vitamin supplement use, mineral supplement use, glucosamine use, aspirin use, and healthy diet score. | | | | | | | | | | | |

| **Table S5. Interactions between fish oil and n-3 PUFAs-associated alleles on T2D risk** | | | | | | |  |  |  |  |  |
| --- | --- | --- | --- | --- | --- | --- | --- | --- | --- | --- | --- |
|  | **Fish oil** | | |  | **Number of alleles** | | |  | **Number of alleles×Fish oil** | | |
|  | **β** | **SE** | ***P*** |  | **β** | **SE** | ***P*** |  | **β** | **SE** | ***P*** |
| **n-3 PUFA-associated alleles** | -0.461 | 0.230 | 0.045 |  | -0.007 | 0.004 | 0.086 |  | 0.013 | 0.008 | 0.099 |
| **long chain n-3 PUFA-associated alleles** | -0.289 | 0.145 | 0.046 |  | -0.005 | 0.003 | 0.080 |  | 0.008 | 0.005 | 0.146 |
| **EPA-associated alleles** | -0.230 | 0.117 | 0.050 |  | -0.011 | 0.007 | 0.094 |  | 0.016 | 0.012 | 0.193 |
| **DPA-associated alleles** | -0.231 | 0.110 | 0.036 |  | -0.027 | 0.012 | 0.022 |  | 0.030 | 0.021 | 0.158 |
| **DHA-associated alleles** | -0.238 | 0.142 | 0.094 |  | -0.006 | 0.006 | 0.373 |  | 0.013 | 0.012 | 0.270 |
| **rs174547** | -0.051 | 0.061 | 0.398 |  | 0.049 | 0.033 | 0.140 |  | -0.063 | 0.060 | 0.297 |
| **rs1535** | -0.043 | 0.061 | 0.483 |  | 0.054 | 0.033 | 0.102 |  | -0.068 | 0.060 | 0.253 |
| **rs174555** | -0.024 | 0.058 | 0.687 |  | 0.064 | 0.034 | 0.060 |  | -0.109 | 0.062 | **0.078** |
| **rs964184** | -0.083 | 0.051 | 0.101 |  | 0.056 | 0.045 | 0.215 |  | -0.024 | 0.083 | 0.771 |
| **rs968567** | -0.066 | 0.053 | 0.213 |  | 0.067 | 0.040 | 0.090 |  | -0.064 | 0.073 | 0.383 |
| **rs7200543** | -0.255 | 0.099 | 0.010 |  | -0.051 | 0.034 | 0.130 |  | 0.117 | 0.062 | **0.061** |
| **rs3798713** | -0.097 | 0.066 | 0.140 |  | -0.041 | 0.031 | 0.192 |  | 0.004 | 0.057 | 0.948 |
| **rs174538** | -0.151 | 0.095 | 0.113 |  | -0.044 | 0.034 | 0.189 |  | 0.042 | 0.061 | 0.496 |
| **rs174556** | -0.236 | 0.098 | 0.016 |  | -0.065 | 0.034 | 0.056 |  | 0.105 | 0.062 | 0.090 |
| **rs174535** | -0.201 | 0.090 | 0.026 |  | -0.057 | 0.033 | 0.081 |  | 0.086 | 0.060 | 0.152 |
| **rs174536** | -0.184 | 0.091 | 0.043 |  | -0.050 | 0.033 | 0.132 |  | 0.072 | 0.060 | 0.231 |
| **rs174546** | -0.192 | 0.091 | 0.036 |  | -0.052 | 0.033 | 0.116 |  | 0.077 | 0.060 | 0.197 |
| **rs2295602** | -0.079 | 0.080 | 0.319 |  | 0.039 | 0.031 | 0.213 |  | -0.009 | 0.057 | 0.873 |
| **rs780094** | -0.180 | 0.063 | 0.004 |  | -0.073 | 0.033 | 0.026 |  | 0.114 | 0.058 | **0.049** |
| **rs3734398** | -0.101 | 0.065 | 0.124 |  | -0.047 | 0.031 | 0.134 |  | 0.008 | 0.057 | 0.887 |
| **rs9393915** | -0.083 | 0.055 | 0.128 |  | 0.014 | 0.037 | 0.705 |  | -0.015 | 0.067 | 0.827 |
| **rs2236212** | -0.097 | 0.081 | 0.229 |  | 0.041 | 0.032 | 0.194 |  | 0.003 | 0.057 | 0.959 |
| **rs102275** | -0.231 | 0.091 | 0.011 |  | -0.056 | 0.033 | 0.090 |  | 0.108 | 0.060 | **0.072** |
| **rs1077989** | -0.141 | 0.074 | 0.058 |  | -0.042 | 0.031 | 0.182 |  | 0.050 | 0.057 | 0.382 |
| **rs3794991** | 0.030 | 0.182 | 0.868 |  | -0.064 | 0.054 | 0.238 |  | -0.066 | 0.097 | 0.496 |
| **rs4711171** | -0.071 | 0.080 | 0.376 |  | 0.041 | 0.031 | 0.194 |  | -0.016 | 0.057 | 0.774 |
| β coefficients were derived from Cox proportional hazard models adjusted for age, sex, race, centers, BMI, education, Townsend deprivation index, household income, smoking, alcohol consumption, physical activity, history of hypertension, history of high cholesterol, vitamin supplement use, mineral supplement use, glucosamine use, aspirin use, and healthy diet score. | | | | | | | | | | | |

| **Table S6. Associations of fish oil use with plasma fatty acids and metabolites** | | | | | |
| --- | --- | --- | --- | --- | --- |
| **NMR metabolites** | **N** | **β** | **SE** | **P value** |  |
| PUFAs | 11528 | 0.066 | 0.02 | <0.001 |  |
| n-3 PUFAs | 11528 | 0.365 | 0.02 | <0.001 |  |
| DHA | 11528 | 0.339 | 0.02 | <0.001 |  |
| non-DHA n-3 PUFAs | 11528 | 0.337 | 0.021 | <0.001 |  |
| n-6/n-3 PUFAs ratio | 11528 | -0.306 | 0.021 | <0.001 |  |
| Pyruvate | 11495 | 0.022 | 0.022 | 0.319 |  |
| Isoleucine | 11534 | 0.002 | 0.022 | 0.925 |  |
| Lactate | 11505 | 0.006 | 0.022 | 0.792 |  |
| Leucine | 11534 | -0.002 | 0.021 | 0.939 |  |
| β coefficients were derived from general linear regression models adjusted for age, sex, race, centers, BMI, education, Townsend deprivation index, household income, smoking, alcohol consumption, physical activity, history of hypertension, history of high cholesterol, vitamin supplement use, mineral supplement use, glucosamine use, aspirin use, and healthy diet score. | | | | |  |

| **Table S7. Interactions between fish oil and n-3 PUFAs-associated SNPs on plasma levels of n-3 PUFAs and n-6/n-3 PUFAs ratio (*n*=10267)** | | | | | | | | | | | | |
| --- | --- | --- | --- | --- | --- | --- | --- | --- | --- | --- | --- | --- |
| **Fatty acids (% of total FAs)** | **SNP** | **SNP** | | |  | **Fish oil** | | |  | **SNP×Fish oil** | | |
|  |  | **β** | **SE** | ***P*** |  | **β** | **SE** | ***P*** |  | **β** | **SE** | ***P*** |
| **Plasma n-3 PUFAs** | **n-3 PUFAs associated alleles** | 0.069 | 0.003 | <0.001 |  | 0.789 | 0.155 | <0.001 |  | -0.009 | 0.005 | **0.079** |
|  | **ALA associated alleles** | -0.111 | 0.006 | <0.001 |  | 0.529 | 0.054 | <0.001 |  | -0.002 | 0.011 | 0.829 |
|  | **Long chain n-3 PUFAs associated alleles** | 0.048 | 0.002 | <0.001 |  | 0.622 | 0.099 | <0.001 |  | -0.004 | 0.004 | 0.281 |
|  | **EPA associated alleles** | 0.107 | 0.005 | <0.001 |  | 0.558 | 0.081 | <0.001 |  | -0.004 | 0.009 | 0.641 |
|  | **DPA associated alleles** | 0.128 | 0.008 | <0.001 |  | 0.557 | 0.076 | <0.001 |  | -0.009 | 0.015 | 0.516 |
|  | **DHA associated alleles** | 0.082 | 0.005 | <0.001 |  | 0.652 | 0.098 | <0.001 |  | -0.011 | 0.008 | 0.166 |
|  | **rs174547** | -0.520 | 0.024 | <0.001 |  | 0.512 | 0.041 | <0.001 |  | 0.016 | 0.041 | 0.705 |
|  | **rs3798713** | -0.003 | 0.023 | 0.894 |  | 0.527 | 0.047 | <0.001 |  | -0.009 | 0.040 | 0.818 |
|  | **rs174538** | 0.487 | 0.024 | <0.001 |  | 0.573 | 0.066 | <0.001 |  | -0.035 | 0.042 | 0.404 |
|  | **rs780094** | 0.073 | 0.024 | 0.002 |  | 0.548 | 0.044 | <0.001 |  | -0.041 | 0.040 | 0.306 |
|  | **rs3734398** | -0.003 | 0.023 | 0.901 |  | 0.512 | 0.047 | <0.001 |  | 0.008 | 0.040 | 0.846 |
|  | **rs2236212** | 0.002 | 0.023 | 0.928 |  | 0.526 | 0.056 | <0.001 |  | -0.006 | 0.040 | 0.876 |
|  | **rs1535** | -0.519 | 0.024 | <0.001 |  | 0.510 | 0.041 | <0.001 |  | 0.016 | 0.041 | 0.694 |
|  | **rs174555** | -0.505 | 0.024 | <0.001 |  | 0.530 | 0.040 | <0.001 |  | -0.010 | 0.042 | 0.817 |
|  | **rs964184** | 0.072 | 0.034 | 0.033 |  | 0.533 | 0.035 | <0.001 |  | -0.052 | 0.059 | 0.378 |
|  | **rs968567** | -0.364 | 0.030 | <0.001 |  | 0.513 | 0.037 | <0.001 |  | 0.027 | 0.051 | 0.600 |
|  | **rs7200543** | 0.168 | 0.025 | <0.001 |  | 0.527 | 0.068 | <0.001 |  | -0.005 | 0.043 | 0.914 |
|  | **rs174556** | 0.502 | 0.024 | <0.001 |  | 0.510 | 0.067 | <0.001 |  | 0.010 | 0.042 | 0.807 |
|  | **rs174535** | 0.513 | 0.023 | <0.001 |  | 0.535 | 0.062 | <0.001 |  | -0.010 | 0.041 | 0.811 |
|  | **rs174536** | 0.515 | 0.023 | <0.001 |  | 0.537 | 0.062 | <0.001 |  | -0.011 | 0.041 | 0.787 |
|  | **rs174546** | 0.520 | 0.024 | <0.001 |  | 0.540 | 0.063 | <0.001 |  | -0.013 | 0.041 | 0.747 |
|  | **rs2295602** | 0.010 | 0.023 | 0.660 |  | 0.540 | 0.056 | <0.001 |  | -0.019 | 0.040 | 0.644 |
|  | **rs9393915** | -0.033 | 0.027 | 0.233 |  | 0.503 | 0.039 | <0.001 |  | 0.036 | 0.047 | 0.439 |
|  | **rs102275** | 0.515 | 0.023 | <0.001 |  | 0.544 | 0.062 | <0.001 |  | -0.019 | 0.041 | 0.647 |
|  | **rs1077989** | 0.016 | 0.023 | 0.483 |  | 0.607 | 0.053 | <0.001 |  | -0.084 | 0.040 | **0.036** |
|  | **rs3794991** | 0.110 | 0.040 | 0.006 |  | 0.692 | 0.132 | <0.001 |  | -0.095 | 0.071 | 0.176 |
|  | **rs4711171** | 0.011 | 0.023 | 0.629 |  | 0.559 | 0.056 | <0.001 |  | -0.036 | 0.040 | 0.376 |
| **Plasma DHA** | **n-3 PUFAs associated alleles** | 0.021 | 0.001 | <0.001 |  | 0.323 | 0.068 | <0.001 |  | -0.004 | 0.002 | 0.091 |
|  | **ALA associated alleles** | -0.036 | 0.003 | <0.001 |  | 0.216 | 0.023 | <0.001 |  | -0.002 | 0.005 | 0.752 |
|  | **Long chain n-3 PUFAs associated alleles** | 0.015 | 0.001 | <0.001 |  | 0.253 | 0.043 | <0.001 |  | -0.002 | 0.002 | 0.299 |
|  | **EPA associated alleles** | 0.034 | 0.002 | <0.001 |  | 0.224 | 0.035 | <0.001 |  | -0.001 | 0.004 | 0.689 |
|  | **DPA associated alleles** | 0.035 | 0.004 | <0.001 |  | 0.230 | 0.033 | <0.001 |  | -0.005 | 0.006 | 0.464 |
|  | **DHA associated alleles** | 0.026 | 0.002 | <0.001 |  | 0.266 | 0.043 | <0.001 |  | -0.005 | 0.003 | 0.178 |
|  | **rs174547** | -0.164 | 0.010 | <0.001 |  | 0.208 | 0.018 | <0.001 |  | 0.004 | 0.018 | 0.806 |
|  | **rs3798713** | -0.008 | 0.010 | 0.402 |  | 0.214 | 0.020 | <0.001 |  | -0.004 | 0.017 | 0.808 |
|  | **rs174538** | 0.157 | 0.011 | <0.001 |  | 0.234 | 0.029 | <0.001 |  | -0.016 | 0.018 | 0.387 |
|  | **rs780094** | -0.005 | 0.010 | 0.605 |  | 0.223 | 0.019 | <0.001 |  | -0.017 | 0.017 | 0.320 |
|  | **rs3734398** | -0.010 | 0.010 | 0.339 |  | 0.205 | 0.020 | <0.001 |  | 0.006 | 0.017 | 0.740 |
|  | **rs2236212** | 0.009 | 0.010 | 0.384 |  | 0.213 | 0.024 | <0.001 |  | -0.002 | 0.017 | 0.896 |
|  | **rs1535** | -0.163 | 0.010 | <0.001 |  | 0.208 | 0.018 | <0.001 |  | 0.004 | 0.018 | 0.812 |
|  | **rs174555** | -0.156 | 0.011 | <0.001 |  | 0.218 | 0.017 | <0.001 |  | -0.011 | 0.019 | 0.557 |
|  | **rs964184** | -0.040 | 0.014 | 0.006 |  | 0.215 | 0.015 | <0.001 |  | -0.024 | 0.025 | 0.350 |
|  | **rs968567** | -0.106 | 0.013 | <0.001 |  | 0.206 | 0.016 | <0.001 |  | 0.013 | 0.022 | 0.545 |
|  | **rs7200543** | 0.062 | 0.011 | <0.001 |  | 0.216 | 0.029 | <0.001 |  | -0.004 | 0.018 | 0.844 |
|  | **rs174556** | 0.155 | 0.011 | <0.001 |  | 0.196 | 0.029 | <0.001 |  | 0.011 | 0.019 | 0.551 |
|  | **rs174535** | 0.163 | 0.010 | <0.001 |  | 0.216 | 0.027 | <0.001 |  | -0.004 | 0.018 | 0.827 |
|  | **rs174536** | 0.163 | 0.010 | <0.001 |  | 0.217 | 0.027 | <0.001 |  | -0.005 | 0.018 | 0.801 |
|  | **rs174546** | 0.164 | 0.010 | <0.001 |  | 0.216 | 0.027 | <0.001 |  | -0.003 | 0.018 | 0.846 |
|  | **rs2295602** | 0.013 | 0.010 | 0.197 |  | 0.223 | 0.024 | <0.001 |  | -0.011 | 0.017 | 0.528 |
|  | **rs9393915** | -0.025 | 0.012 | 0.035 |  | 0.209 | 0.017 | <0.001 |  | 0.003 | 0.020 | 0.880 |
|  | **rs102275** | 0.163 | 0.010 | <0.001 |  | 0.218 | 0.027 | <0.001 |  | -0.006 | 0.018 | 0.753 |
|  | **rs1077989** | 0.006 | 0.010 | 0.565 |  | 0.256 | 0.023 | <0.001 |  | -0.043 | 0.017 | **0.012** |
|  | **rs3794991** | -0.002 | 0.017 | 0.910 |  | 0.258 | 0.057 | <0.001 |  | -0.026 | 0.030 | 0.391 |
|  | **rs4711171** | 0.014 | 0.010 | 0.169 |  | 0.232 | 0.024 | <0.001 |  | -0.019 | 0.017 | 0.261 |
| **Plasma non-DHA n-3 PUFAs** | **n-3 PUFAs associated alleles** | 0.048 | 0.002 | <0.001 |  | 0.465 | 0.103 | <0.001 |  | -0.005 | 0.003 | 0.126 |
|  | **ALA associated alleles** | -0.075 | 0.004 | <0.001 |  | 0.313 | 0.036 | <0.001 |  | -0.001 | 0.007 | 0.906 |
|  | **Long chain n-3 PUFAs associated alleles** | 0.033 | 0.001 | <0.001 |  | 0.369 | 0.066 | <0.001 |  | -0.002 | 0.002 | 0.348 |
|  | **EPA associated alleles** | 0.073 | 0.003 | <0.001 |  | 0.334 | 0.054 | <0.001 |  | -0.002 | 0.006 | 0.661 |
|  | **DPA associated alleles** | 0.094 | 0.006 | <0.001 |  | 0.327 | 0.051 | <0.001 |  | -0.005 | 0.010 | 0.618 |
|  | **DHA associated alleles** | 0.055 | 0.003 | <0.001 |  | 0.386 | 0.065 | <0.001 |  | -0.006 | 0.005 | 0.229 |
|  | **rs174547** | -0.356 | 0.016 | <0.001 |  | 0.304 | 0.027 | <0.001 |  | 0.011 | 0.027 | 0.684 |
|  | **rs3798713** | 0.005 | 0.016 | 0.734 |  | 0.313 | 0.031 | <0.001 |  | -0.005 | 0.027 | 0.850 |
|  | **rs174538** | 0.330 | 0.016 | <0.001 |  | 0.339 | 0.044 | <0.001 |  | -0.019 | 0.028 | 0.492 |
|  | **rs780094** | 0.079 | 0.016 | <0.001 |  | 0.325 | 0.029 | <0.001 |  | -0.024 | 0.027 | 0.371 |
|  | **rs3734398** | 0.007 | 0.016 | 0.667 |  | 0.307 | 0.031 | <0.001 |  | 0.002 | 0.027 | 0.939 |
|  | **rs2236212** | -0.007 | 0.016 | 0.670 |  | 0.313 | 0.038 | <0.001 |  | -0.004 | 0.027 | 0.881 |
|  | **rs1535** | -0.355 | 0.016 | <0.001 |  | 0.302 | 0.027 | <0.001 |  | 0.012 | 0.027 | 0.663 |
|  | **rs174555** | -0.349 | 0.016 | <0.001 |  | 0.312 | 0.026 | <0.001 |  | 0.001 | 0.028 | 0.971 |
|  | **rs964184** | 0.111 | 0.022 | <0.001 |  | 0.318 | 0.023 | <0.001 |  | -0.028 | 0.039 | 0.471 |
|  | **rs968567** | -0.258 | 0.020 | <0.001 |  | 0.306 | 0.024 | <0.001 |  | 0.013 | 0.034 | 0.693 |
|  | **rs7200543** | 0.106 | 0.017 | <0.001 |  | 0.311 | 0.045 | <0.001 |  | -0.001 | 0.029 | 0.972 |
|  | **rs174556** | 0.347 | 0.016 | <0.001 |  | 0.314 | 0.045 | <0.001 |  | -0.001 | 0.028 | 0.982 |
|  | **rs174535** | 0.350 | 0.016 | <0.001 |  | 0.319 | 0.041 | <0.001 |  | -0.006 | 0.027 | 0.829 |
|  | **rs174536** | 0.351 | 0.016 | <0.001 |  | 0.320 | 0.041 | <0.001 |  | -0.007 | 0.027 | 0.810 |
|  | **rs174546** | 0.356 | 0.016 | <0.001 |  | 0.324 | 0.042 | <0.001 |  | -0.010 | 0.027 | 0.721 |
|  | **rs2295602** | -0.003 | 0.016 | 0.862 |  | 0.317 | 0.037 | <0.001 |  | -0.008 | 0.027 | 0.774 |
|  | **rs9393915** | -0.008 | 0.018 | 0.667 |  | 0.293 | 0.026 | <0.001 |  | 0.033 | 0.031 | 0.288 |
|  | **rs102275** | 0.353 | 0.016 | <0.001 |  | 0.327 | 0.041 | <0.001 |  | -0.013 | 0.027 | 0.630 |
|  | **rs1077989** | 0.011 | 0.015 | 0.496 |  | 0.351 | 0.035 | <0.001 |  | -0.040 | 0.027 | 0.130 |
|  | **rs3794991** | 0.112 | 0.027 | <0.001 |  | 0.434 | 0.088 | <0.001 |  | -0.069 | 0.047 | 0.140 |
|  | **rs4711171** | -0.003 | 0.016 | 0.871 |  | 0.327 | 0.037 | <0.001 |  | -0.016 | 0.027 | 0.547 |
| **Plasma n-6/n-3 PUFAs** | **n-3 PUFAs associated alleles** | -0.262 | 0.009 | <0.001 |  | -4.266 | 0.455 | <0.001 |  | 0.104 | 0.015 | **<0.001** |
|  | **ALA associated alleles** | 0.423 | 0.019 | <0.001 |  | -0.733 | 0.159 | <0.001 |  | -0.129 | 0.033 | **<0.001** |
|  | **Long chain n-3 PUFAs associated alleles** | -0.180 | 0.006 | <0.001 |  | -2.910 | 0.289 | <0.001 |  | 0.066 | 0.011 | **<0.001** |
|  | **EPA associated alleles** | -0.403 | 0.014 | <0.001 |  | -2.485 | 0.236 | <0.001 |  | 0.140 | 0.025 | **<0.001** |
|  | **DPA associated alleles** | -0.507 | 0.025 | <0.001 |  | -2.170 | 0.225 | <0.001 |  | 0.199 | 0.043 | **<0.001** |
|  | **DHA associated alleles** | -0.305 | 0.014 | <0.001 |  | -2.574 | 0.289 | <0.001 |  | 0.113 | 0.024 | **<0.001** |
|  | **rs174547** | 1.948 | 0.069 | <0.001 |  | -0.816 | 0.121 | <0.001 |  | -0.660 | 0.121 | **<0.001** |
|  | **rs3798713** | -0.029 | 0.069 | 0.674 |  | -1.302 | 0.139 | <0.001 |  | 0.068 | 0.119 | 0.568 |
|  | **rs174538** | -1.811 | 0.071 | <0.001 |  | -2.229 | 0.194 | <0.001 |  | 0.699 | 0.124 | **<0.001** |
|  | **rs780094** | -0.362 | 0.070 | <0.001 |  | -1.349 | 0.130 | <0.001 |  | 0.152 | 0.120 | 0.204 |
|  | **rs3734398** | -0.040 | 0.069 | 0.565 |  | -1.300 | 0.138 | <0.001 |  | 0.066 | 0.119 | 0.578 |
|  | **rs2236212** | 0.034 | 0.069 | 0.623 |  | -1.189 | 0.168 | <0.001 |  | -0.047 | 0.119 | 0.696 |
|  | **rs1535** | 1.945 | 0.069 | <0.001 |  | -0.802 | 0.121 | <0.001 |  | -0.668 | 0.120 | **<0.001** |
|  | **rs174555** | 1.928 | 0.071 | <0.001 |  | -0.910 | 0.117 | <0.001 |  | -0.594 | 0.124 | **<0.001** |
|  | **rs964184** | -0.414 | 0.100 | <0.001 |  | -1.329 | 0.105 | <0.001 |  | 0.305 | 0.175 | 0.081 |
|  | **rs968567** | 1.396 | 0.087 | <0.001 |  | -1.044 | 0.108 | <0.001 |  | -0.580 | 0.151 | **<0.001** |
|  | **rs7200543** | -0.489 | 0.074 | <0.001 |  | -1.505 | 0.202 | <0.001 |  | 0.184 | 0.127 | 0.149 |
|  | **rs174556** | -1.918 | 0.071 | <0.001 |  | -2.094 | 0.197 | <0.001 |  | 0.590 | 0.124 | **<0.001** |
|  | **rs174535** | -1.932 | 0.069 | <0.001 |  | -2.133 | 0.182 | <0.001 |  | 0.665 | 0.120 | **<0.001** |
|  | **rs174536** | -1.932 | 0.069 | <0.001 |  | -2.124 | 0.183 | <0.001 |  | 0.656 | 0.120 | **<0.001** |
|  | **rs174546** | -1.948 | 0.069 | <0.001 |  | -2.131 | 0.184 | <0.001 |  | 0.658 | 0.121 | **<0.001** |
|  | **rs2295602** | 0.025 | 0.069 | 0.719 |  | -1.205 | 0.165 | <0.001 |  | -0.034 | 0.119 | 0.778 |
|  | **rs9393915** | 0.028 | 0.081 | 0.734 |  | -1.241 | 0.114 | <0.001 |  | -0.008 | 0.140 | 0.954 |
|  | **rs102275** | -1.926 | 0.069 | <0.001 |  | -2.113 | 0.182 | <0.001 |  | 0.658 | 0.120 | **<0.001** |
|  | **rs1077989** | -0.142 | 0.069 | 0.039 |  | -1.463 | 0.156 | <0.001 |  | 0.210 | 0.118 | 0.076 |
|  | **rs3794991** | -0.559 | 0.120 | <0.001 |  | -1.842 | 0.392 | <0.001 |  | 0.331 | 0.209 | 0.113 |
|  | **rs4711171** | 0.017 | 0.069 | 0.802 |  | -1.225 | 0.165 | <0.001 |  | -0.016 | 0.119 | 0.893 |
| β coefficients were derived from general linear regression models adjusted for age, sex, race, centers, BMI, education, Townsend deprivation index, household income, smoking, alcohol consumption, physical activity, history of hypertension, history of high cholesterol, vitamin supplement use, mineral supplement use, glucosamine use, aspirin use, and healthy diet score. | | | | | | | | | | | | |

| **Table S8. The *P* value and FDR of potential biomarkers (VIP>1 for EPA/DHA group) in male and female mice** | | | |
| --- | --- | --- | --- |
| **Group** | **Compound name** | ***P* Value** | **FDR** |
| Male | Lactic acid | 0.0054 | 0.0756 |
|  | 8-Hydroxyguanosine | 0.0572 | 0.4004 |
|  | Leucyl-Valine | 0.1864 | 0.8699 |
|  | 3-Methylhistidine | 0.2436 | 0.8526 |
|  | L-Isoleucyl-l-alanyl-L-arginine | 0.3074 | 0.8607 |
|  | Valyl-Valine | 0.3218 | 0.7509 |
|  | Creatine | 0.3485 | 0.6970 |
|  | 2,4-Diaminobutyric acid | 0.3487 | 0.6102 |
|  | Fumaric acid | 0.3887 | 0.6046 |
|  | Malic acid | 0.42 | 0.5880 |
|  | Acetylcarnosine | 0.4255 | 0.5415 |
|  | CMP-Sialic acid | 0.4658 | 0.5434 |
|  | Glyceric acid | 0.5152 | 0.5548 |
|  | β-Alanine | 0.5833 | 0.5833 |
| Female | 8-Hydroxyguanosine | 0.0214 | 0.2996 |
|  | Pyruvic acid | 0.0368 | 0.2576 |
|  | Fumaric acid | 0.0572 | 0.2669 |
|  | 3-Hydroxybutyric acid | 0.0605 | 0.2118 |
|  | Leucyl-Glutamine | 0.0713 | 0.1996 |
|  | Malic acid | 0.185 | 0.4317 |
|  | LysoPE(22:5) | 0.2716 | 0.5432 |
|  | Creatine | 0.3058 | 0.5352 |
|  | L-Isoleucyl-l-alanyl-L-arginine | 0.3665 | 0.5701 |
|  | Glyceric acid | 0.3933 | 0.5506 |
|  | Fructose 6-phosphate | 0.4297 | 0.5469 |
|  | Ureidopropionic acid | 0.4484 | 0.5231 |
|  | Arginyl-L-arginine | 0.6081 | 0.6549 |
|  | CMP-Sialic acid | 0.637 | 0.6370 |
| The ANOVA analysis was performed in each potential biomarker (screened by VIP>1). Subsequently, false fiscovery rate (FDR) was calculated. | | | |

| **Table S9. Associations of plasma n-3 PUFAs with pyruvate, lactate, isoleucine and leucine** | | | | | | | | | | |  |  |  |  |  |
| --- | --- | --- | --- | --- | --- | --- | --- | --- | --- | --- | --- | --- | --- | --- | --- |
| **NMR metabolites** | | **Total** | | | |  | **Women** | | | |  | **Men** | | | |
|  |  | **N** | **β** | **SE** | **P value** |  | **N** | **β** | **SE** | **P value** |  | **N** | **β** | **SE** | **P value** |
| Pyruvate | n-3 PUFAs | 11489 | -0.017 | 0.006 | 0.006 |  | 6426 | -0.014 | 0.009 | 0.098 |  | 5063 | -0.024 | 0.009 | 0.010 |
|  | DHA | 11489 | -0.063 | 0.015 | <0.001 |  | 6426 | -0.045 | 0.020 | 0.026 |  | 5063 | -0.091 | 0.021 | <0.001 |
|  | Non-DHA n-3 PUFAs | 11489 | -0.013 | 0.010 | 0.173 |  | 6426 | -0.015 | 0.013 | 0.269 |  | 5063 | -0.014 | 0.014 | 0.343 |
|  | n-6/n-3 PUFAs | 11489 | -0.001 | 0.002 | 0.708 |  | 6426 | -0.001 | 0.003 | 0.676 |  | 5063 | 0.000 | 0.003 | 0.999 |
| Lactate | n-3 PUFAs | 11505 | -0.016 | 0.006 | 0.010 |  | 6429 | -0.020 | 0.008 | 0.019 |  | 5076 | -0.020 | 0.010 | 0.039 |
|  | DHA | 11505 | -0.095 | 0.015 | <0.001 |  | 6429 | -0.095 | 0.020 | <0.001 |  | 5076 | -0.107 | 0.021 | <0.001 |
|  | Non-DHA n-3 PUFAs | 11505 | 0.003 | 0.010 | 0.767 |  | 6429 | -0.007 | 0.013 | 0.600 |  | 5076 | 0.004 | 0.015 | 0.778 |
|  | n-6/n-3 PUFAs | 11505 | -0.007 | 0.002 | 0.002 |  | 6429 | -0.004 | 0.003 | 0.234 |  | 5076 | -0.008 | 0.003 | 0.008 |
| Isoleucine | n-3 PUFAs | 11528 | 0.046 | 0.006 | <0.001 |  | 6442 | 0.030 | 0.008 | <0.001 |  | 5086 | 0.058 | 0.010 | <0.001 |
|  | DHA | 11528 | -0.096 | 0.015 | <0.001 |  | 6442 | -0.107 | 0.020 | <0.001 |  | 5086 | -0.093 | 0.022 | <0.001 |
|  | Non-DHA n-3 PUFAs | 11528 | 0.148 | 0.010 | <0.001 |  | 6442 | 0.114 | 0.013 | <0.001 |  | 5086 | 0.179 | 0.015 | <0.001 |
|  | n-6/n-3 PUFAs | 11528 | -0.027 | 0.002 | <0.001 |  | 6442 | -0.024 | 0.003 | <0.001 |  | 5086 | -0.028 | 0.003 | <0.001 |
| Leucine | n-3 PUFAs | 11528 | 0.023 | 0.006 | <0.001 |  | 6442 | 0.000 | 0.008 | 0.975 |  | 5086 | 0.045 | 0.010 | <0.001 |
|  | DHA | 11528 | -0.134 | 0.015 | <0.001 |  | 6442 | -0.162 | 0.019 | <0.001 |  | 5086 | -0.106 | 0.022 | <0.001 |
|  | Non-DHA n-3 PUFAs | 11528 | 0.110 | 0.010 | <0.001 |  | 6442 | 0.066 | 0.012 | <0.001 |  | 5086 | 0.156 | 0.015 | <0.001 |
|  | n-6/n-3 PUFAs | 11528 | -0.023 | 0.002 | <0.001 |  | 6442 | -0.017 | 0.003 | <0.001 |  | 5086 | -0.027 | 0.003 | <0.001 |
| β coefficients were derived from general linear regression models adjusted for age, sex, race, centers, BMI, education, Townsend deprivation index, household income, smoking, alcohol consumption, physical activity, history of hypertension, history of high cholesterol, vitamin supplement use, mineral supplement use, glucosamine use, aspirin use, and healthy diet score. | | | | | | | | | | | | | | | |

| **Table S10. The biomarkers of each group in metabolome analysis from OPLS-DA models excluding outlier animals** | | |
| --- | --- | --- |
| **Group** | **Compound name** | **VIP value** |
| Male DHA | Lactic acid | 1.565 |
|  | 8-Hydroxyguanosine | 1.511 |
|  | Leucyl-Valine | 1.443 |
|  | Valyl-Valine | 1.389 |
|  | Malic acid | 1.329 |
|  | 2,4-Diaminobutyric acid | 1.251 |
|  | Fumaric acid | 1.250 |
|  | β-Alanine | 1.112 |
|  | L-Isoleucyl-l-alanyl-L-arginine | 1.106 |
|  | Acetylcarnosine | 1.105 |
|  | CMP-Sialic acid | 1.013 |
| Male EPA | Lactic acid | 1.553 |
|  | Malic acid | 1.264 |
|  | Glyceric acid | 1.148 |
|  | Fumaric acid | 1.041 |
|  | 8-Hydroxyguanosine | 1.664 |
|  | Glucosaminic acid 6-phosphate | 1.284 |
|  | CMP-Sialic acid | 1.140 |
|  | 3-Methylhistidine | 1.020 |
|  | Leucyl-Valine | 1.017 |
| Female DHA | 3-Hydroxybutyric acid | 1.519 |
|  | Pyruvic acid | 1.328 |
|  | L-Isoleucyl-l-alanyl-L-arginine | 1.286 |
|  | Arginyl-L-arginine | 1.253 |
|  | Creatine | 1.231 |
|  | LysoPE(22:5) | 1.199 |
|  | Fumaric acid | 1.181 |
|  | CMP-Sialic acid | 1.126 |
|  | Glyceric acid | 1.125 |
|  | Leucyl-Glutamine | 1.114 |
|  | Fructose 6-phosphate | 1.082 |
|  | Ureidopropionic acid | 1.067 |
| Female EPA | L-Isoleucyl-l-alanyl-L-arginine | 1.569 |
|  | Pyruvic acid | 1.509 |
|  | 8-Hydroxyguanosine | 1.441 |
|  | Fumaric acid | 1.371 |
|  | Creatine | 1.340 |
|  | LysoPE(22:5) | 1.227 |
|  | Malic acid | 1.171 |
|  | 3-Hydroxybutyric acid | 1.127 |
|  | Glyceric acid | 1.102 |
|  | Ureidopropionic acid | 1.062 |
|  | Fructose 6-phosphate | 1.033 |

| **Table S11. Interactions between fish oil use and SNPs of GLUT4, GYS1/2, GSK3β and PDPR on T2D risk** | | | | | | | | | | | |  |  |
| --- | --- | --- | --- | --- | --- | --- | --- | --- | --- | --- | --- | --- | --- |
| **SNP†** | **Related gene** | **N** | **Fish oil** | | |  | **SNP** | | |  | **SNP×Fish oil** | | |
|  |  |  | **β*** | **SE** | ***P*** |  | **β*** | **SE** | ***P*** |  | **β*** | **SE** | ***P*** |
| **Women** |  |  |  |  |  |  |  |  |  |  |  |  |  |
| **rs5418** | **GLUT4** | 24169 | -0.224 | 0.101 | 0.026 |  | 0.023 | 0.047 | 0.619 |  | 0.092 | 0.085 | 0.278 |
| **rs5435** | **GLUT4** | 24169 | -0.291 | 0.096 | 0.003 |  | -0.035 | 0.048 | 0.469 |  | 0.187 | 0.086 | **0.030** |
| **rs5417** | **GLUT4** | 24169 | -0.224 | 0.101 | 0.027 |  | 0.023 | 0.047 | 0.625 |  | 0.091 | 0.085 | 0.280 |
| **rs8082645** | **GLUT4** | 24169 | -0.273 | 0.198 | 0.168 |  | 0.086 | 0.067 | 0.198 |  | 0.088 | 0.123 | 0.476 |
| **rs7409311** | **GYS1** | 24169 | 0.033 | 0.324 | 0.919 |  | 0.146 | 0.097 | 0.131 |  | -0.093 | 0.169 | 0.582 |
| **rs140496340** | **GYS1** | 24169 | -0.021 | 0.102 | 0.835 |  | 0.106 | 0.047 | 0.025 |  | -0.134 | 0.086 | 0.118 |
| **rs62125989** | **GYS1** | 24169 | -0.003 | 0.144 | 0.985 |  | 0.094 | 0.051 | 0.066 |  | -0.099 | 0.092 | 0.281 |
| **rs61928672** | **GYS2** | 24169 | -0.035 | 0.212 | 0.870 |  | 0.022 | 0.066 | 0.738 |  | -0.063 | 0.119 | 0.596 |
| **rs187630** | **GYS2** | 24169 | -0.282 | 0.148 | 0.057 |  | -0.023 | 0.051 | 0.643 |  | 0.100 | 0.093 | 0.285 |
| **rs6438552** | **GSK3B** | 24169 | -0.217 | 0.128 | 0.090 |  | 0.013 | 0.047 | 0.781 |  | 0.060 | 0.087 | 0.489 |
| **rs334558** | **GSK3B** | 24169 | -0.292 | 0.139 | 0.036 |  | -0.017 | 0.049 | 0.730 |  | 0.111 | 0.090 | 0.218 |
| **rs3755557** | **GSK3B** | 24169 | -0.015 | 0.220 | 0.946 |  | 0.036 | 0.069 | 0.604 |  | -0.073 | 0.122 | 0.548 |
| **rs201013643** | **PDPR** | 24169 | 176.424 | 314.260 | 0.575 |  | 113.743 | 147.655 | 0.441 |  | -88.283 | 157.131 | 0.574 |
| **Men** |  |  |  |  |  |  |  |  |  |  |  |  |  |
| **rs5418** | **GLUT4** | 18740 | -0.028 | 0.091 | 0.758 |  | -0.023 | 0.043 | 0.595 |  | -0.017 | 0.079 | 0.833 |
| **rs5435** | **GLUT4** | 18740 | 0.011 | 0.087 | 0.895 |  | -0.020 | 0.044 | 0.650 |  | -0.070 | 0.081 | 0.385 |
| **rs5417** | **GLUT4** | 18740 | -0.026 | 0.091 | 0.771 |  | -0.023 | 0.043 | 0.598 |  | -0.018 | 0.079 | 0.814 |
| **rs8082645** | **GLUT4** | 18740 | -0.379 | 0.182 | 0.038 |  | -0.004 | 0.061 | 0.948 |  | 0.226 | 0.114 | **0.047** |
| **rs7409311** | **GYS1** | 18740 | -0.280 | 0.290 | 0.334 |  | -0.074 | 0.082 | 0.363 |  | 0.128 | 0.152 | 0.400 |
| **rs140496340** | **GYS1** | 18740 | -0.017 | 0.092 | 0.855 |  | -0.067 | 0.044 | 0.127 |  | -0.031 | 0.080 | 0.702 |
| **rs62125989** | **GYS1** | 18740 | 0.007 | 0.135 | 0.957 |  | 0.054 | 0.048 | 0.258 |  | -0.035 | 0.086 | 0.682 |
| **rs61928672** | **GYS2** | 18740 | -0.048 | 0.194 | 0.803 |  | -0.041 | 0.060 | 0.494 |  | 0.004 | 0.109 | 0.973 |
| **rs187630** | **GYS2** | 18740 | 0.090 | 0.134 | 0.500 |  | 0.017 | 0.048 | 0.721 |  | -0.095 | 0.086 | 0.266 |
| **rs6438552** | **GSK3B** | 18740 | -0.043 | 0.116 | 0.711 |  | -0.017 | 0.044 | 0.704 |  | 0.001 | 0.079 | 0.992 |
| **rs334558** | **GSK3B** | 18740 | -0.028 | 0.125 | 0.821 |  | -0.004 | 0.045 | 0.936 |  | -0.010 | 0.082 | 0.901 |
| **rs3755557** | **GSK3B** | 18740 | 0.030 | 0.205 | 0.883 |  | 0.021 | 0.062 | 0.739 |  | -0.042 | 0.113 | 0.712 |
| **rs201013643** | **PDPR** | 18740 | 596.419 | 490.493 | 0.224 |  | 300.053 | 245.035 | 0.221 |  | -298.232 | 245.247 | 0.224 |
| *β coefficients were derived from Cox proportional hazard models adjusted for age, sex, race, centers, BMI, education, Townsend deprivation index, household income, smoking, alcohol consumption, physical activity, history of hypertension, history of high cholesterol, vitamin supplement use, mineral supplement use, glucosamine use, aspirin use, and healthy diet score. | | | | | | | | | | | | | |
| **†**rs5418 and rs5435 were referenced from Yu, F. *et al*. GLUT4 gene rs5418 polymorphism is associated with increased coronary heart disease risk in a Uygur Chinese population. *BMC Cardiovasc Disord.* **22**, 191 (2022). rs5417 and rs8082645 were referenced from Malodobra-Mazur, M. *et al*. Single nucleotide polymorphisms in 5'-UTR of the SLC2A4 gene regulate solute carrier family 2 member 4 gene expression in visceral adipose tissue. *Gene*. **576**, 499–504 (2016). rs7409311, rs140496340, rs62125989, rs61928672, rs187630 were referenced from Yang, L. *et al*. Genetic variants of glycogen metabolism genes were associated with liver PDFF without increasing NAFLD Risk. *Front. Genet.* **13**, 830445 (2022). rs6438552 and rs334558 were referenced from Lin, Y. F., Huang, M. C., and Liu, H. C. Glycogen synthase kinase 3β gene polymorphisms may be associated with bipolar I disorder and the therapeutic response to lithium. *J. Affect. Disord*. **147**, 401–406 (2013). rs3755557 was referenced from Shim, S. H. *et al*. Association between glycogen synthase kinase-3β gene polymorphisms and attention deficit hyperactivity disorder in Korean children: a preliminary study. *Prog. Neuropsychopharmacol. Biol. Psychiatry.* ***39***, 57–61 (2012). rs201013643 was referenced from Riveros-Mckay, F. *et al*. The influence of rare variants in circulating metabolic biomarkers. *PLoS Genet*. ***16***, e1008605 (2020). | | | | | | | | | | | | | |

| **Table S12. Spearman correlations between fish oil use assessed at baseline and in resurvey** | | | |
| --- | --- | --- | --- |
|  | **Baseline** | **First repeat assessment (2012-13)*** | **Imaging visit (2014+)**† |
| **Baseline** | 1.00 | 0.61 | 0.47 |
| **First repeat assessment (2012-13)** |  | 1.00 | 0.62 |
| **Imaging visit (2014+)** |  |  | 1.00 |
| *Data were available for 16,469 participants. | | |  |
| †Data were available for 41,612 participants. | | |  |

| **Table S13. Diet component definitions used in the UK Biobank study** | | | |
| --- | --- | --- | --- |
| **Components** | **Intake goal** | **Field IDs** | **Amount per serving** |
| Fruit | 3 servings/day | 1309 (pieces fresh fruit/day) 1319 (pieces dried fruit/day) | 1309 – 1 piece |
|  |  |  | 1319 – 5 pieces |
| Vegetable | 3 servings/day | 1289 (tablespoons cooked vegetables/day) 1299 (salad/raw vegetables/day) | 3 heaped tablespoons |
| Whole grains | 3 servings/day | 1438, 1448 (wholemeal/wholegrain bread slices/week) | 1438/1448 – 1 slice/day 1458/1468 – 1 bowl/day |
|  |  | 1458, 1468 (bran/oat/muesli cereal bowls/week) |  |
| Fish | ≥2 servings/week | 1329 (oily fish/week) | Once/week |
|  |  | 1339 (non-oily fish/week) |  |
| Dairy | 2 servings/day | 1408 (cheese/week) | 1408 – 1 piece/day |
|  |  | 1418 (milk type) | 1418 – 1 glass/day if consumption of any type of milk |
| Vegetable oils | 2 servings/day | 1428 (Flora Pro-Active/Benecol spread) | 1 serving/day if in combination with eating at least 2 slices of bread (ID 1438) |
|  |  | 2654 (Flora Pro-Active/Benecol, soft margarine -, olive oil based -, polyunsaturated/sunflower oil based -, other low/reduced fat spread) |  |
|  |  | 1438 (bread slices/week) |  |
| Refined grains | ≤2 servings/day | 1438, 1448 (white, brown, other bread slices/week) 1458, 1468 (biscuit, other cereals/week) | 1438/1448 – 1 slice/day 1458/1468 – 1 bowl/day |
| Processed meats | ≤1 serving/week | 1349 (processed meat/week or daily) 3680 (age when last ate meat) | 1349 – 1 piece/day |
|  |  |  | 3680 – 0 pieces/day if indicated having never eaten meat |
| Unprocessed meats | ≤2 serving/wk | 1359 (poultry/week or day) 1369 (beef/week or day) | 1359-1389 – once/week |
|  |  | 1379 (lamb or mutton/week or day) 1389 (pork/week or day) | 3680 – 0 pieces/day if indicated having never eaten meat |
|  |  | 3680 (age when last ate meat) |  |
| Sugar-sweetened beverages | Don’t drink | 6144 (never consumes drinks containing sugar) | 0 servings |
| Field IDs and serving sizes used per diet component in UK Biobank with available data from the general baseline questionnaire. If participants achieved the intake goal they were given 1 point for the diet component. The total healthy diet score was the sum of all the diet component scores and ranged from 0 to 10. | | | |

| **Table S14. Definitions of prevalent diabetes and incident T2D** | | | |  |
| --- | --- | --- | --- | --- |
|  | ICD-9 | ICD-10 | Self-reported fields |  |
| Diabetes at baseline [Both “possible” diabetes (T1D, T2D and other types of diabetes) and “probable” diabetes (T1D, T2D and other types of diabetes) were excluded at baseline.]* | 250, 6480 | E10, E11, E12, E13, E14, O24 | 2443(1), 2976, 6153(3), 6177(3), 20002(1220, 1222, 1223), 20003 |  |
|  |  |  |  |  |
|  |  |  |  |  |
|  |  |  |  |  |
|  |  |  |  |  |
| Incident T2D |  | E11 |  |  |
| *Eastwood SV, Mathur R, Atkinson M, et al. Algorithms for the capture and adjudication of prevalent and incident diabetes in UK Biobank. PLoS One. 2016. 11(9): e0162388. | | | |  |

| **Table S15. Characteristics of 424 T2D-associated SNPs in the UK biobank** | | | |  |  |
| --- | --- | --- | --- | --- | --- |
| **rsID** | **Chr** | **leadSNP** | **Gene with fine-mapped coding variant** | **EAF** | **β** |
| rs197379 | 1 | chr1:112292303 |  | 0.380 | 0.027 |
| rs4845987 | 1 | chr1:11306279 |  | 0.709 | 0.028 |
| rs1127215 | 1 | chr1:117532790 | PTGFRN | 0.580 | 0.043 |
| rs41276588 | 1 | chr1:118148384 |  | 0.285 | 0.038 |
| rs1493694 | 1 | chr1:120526982 |  | 0.107 | 0.071 |
| rs79489938 | 1 | chr1:147121000 |  | 0.984 | 0.113 |
| rs72692805 | 1 | chr1:149894355 | MTMR11 | 0.921 | 0.054 |
| rs145904381 | 1 | chr1:151017991 |  | 0.988 | 0.174 |
| rs1194606 | 1 | chr1:154294260 |  | 0.235 | 0.030 |
| rs3020781 | 1 | chr1:155269776 |  | 0.265 | 0.033 |
| rs4916253 | 1 | chr1:172361032 |  | 0.433 | 0.027 |
| rs545608 | 1 | chr1:177899121 |  | 0.206 | 0.036 |
| rs567185 | 1 | chr1:201763499 | SHISA4 | 0.649 | 0.037 |
| rs12048743 | 1 | chr1:205114873 |  | 0.430 | 0.032 |
| rs7538321 | 1 | chr1:205789455 |  | 0.127 | 0.042 |
| rs2336938 | 1 | chr1:206618799 |  | 0.477 | 0.030 |
| rs10916784 | 1 | chr1:20729451 |  | 0.581 | 0.027 |
| rs340874 | 1 | chr1:214159256 |  | 0.568 | 0.067 |
| rs2820444 | 1 | chr1:219741820 |  | 0.702 | 0.047 |
| rs348330 | 1 | chr1:229672955 |  | 0.367 | 0.053 |
| rs10737818 | 1 | chr1:235542023 |  | 0.643 | 0.040 |
| rs6685701 | 1 | chr1:26868639 | RPS6KA1 | 0.271 | 0.030 |
| rs111824905 | 1 | chr1:28110797 |  | 0.052 | 0.069 |
| rs10915188 | 1 | chr1:29024956 |  | 0.583 | 0.029 |
| rs61779275 | 1 | chr1:39820310 | MACF1 | 0.212 | 0.075 |
| rs3176466 | 1 | chr1:51438365 |  | 0.910 | 0.064 |
| rs2269247 | 1 | chr1:64107284 |  | 0.818 | 0.035 |
| rs10889560 | 1 | chr1:65989878 |  | 0.082 | 0.047 |
| rs12741141 | 1 | chr1:6669970 |  | 0.355 | 0.040 |
| rs4655617 | 1 | chr1:67010654 |  | 0.440 | 0.028 |
| rs2613503 | 1 | chr1:72839774 |  | 0.803 | 0.039 |
| rs34506349 | 2 | chr2:100598726 |  | 0.959 | 0.068 |
| rs17624303 | 2 | chr2:105148418 |  | 0.730 | 0.029 |
| rs72836348 | 2 | chr2:111888043 |  | 0.898 | 0.056 |
| rs34589210 | 2 | chr2:112795492 |  | 0.145 | 0.039 |
| rs9784137 | 2 | chr2:121325908 |  | 0.845 | 0.061 |
| rs2033159 | 2 | chr2:145261174 |  | 0.229 | 0.035 |
| rs7609422 | 2 | chr2:146348037 |  | 0.408 | 0.030 |
| rs7559658 | 2 | chr2:147920213 |  | 0.189 | 0.034 |
| rs13020443 | 2 | chr2:152167830 | NMI | 0.508 | 0.031 |
| rs7568172 | 2 | chr2:158335340 |  | 0.937 | 0.067 |
| rs6432613 | 2 | chr2:161145612 |  | 0.724 | 0.039 |
| rs13389219 | 2 | chr2:165528876 |  | 0.606 | 0.065 |
| rs11680058 | 2 | chr2:16574669 |  | 0.870 | 0.056 |
| rs12992995 | 2 | chr2:175197545 |  | 0.724 | 0.031 |
| rs6715901 | 2 | chr2:179650954 |  | 0.503 | 0.027 |
| rs6741676 | 2 | chr2:181618654 |  | 0.662 | 0.032 |
| rs7558413 | 2 | chr2:18721662 |  | 0.580 | 0.029 |
| rs12463719 | 2 | chr2:203450680 |  | 0.283 | 0.032 |
| rs34329895 | 2 | chr2:208870017 |  | 0.396 | 0.028 |
| rs13005841 | 2 | chr2:212302573 |  | 0.708 | 0.029 |
| rs17354348 | 2 | chr2:213835977 |  | 0.743 | 0.029 |
| rs2972145 | 2 | chr2:227101309 |  | 0.646 | 0.090 |
| rs7561798 | 2 | chr2:228973660 |  | 0.481 | 0.028 |
| rs838735 | 2 | chr2:234324192 | SAG | 0.385 | 0.029 |
| rs34845373 | 2 | chr2:25635771 |  | 0.727 | 0.037 |
| rs72803684 | 2 | chr2:26192802 |  | 0.047 | 0.069 |
| rs1260326 | 2 | chr2:27730940 | GCKR | 0.604 | 0.064 |
| rs13414140 | 2 | chr2:43671176 | THADA | 0.885 | 0.118 |
| rs980183 | 2 | chr2:59311536 |  | 0.397 | 0.036 |
| rs243018 | 2 | chr2:60586707 |  | 0.451 | 0.056 |
| rs2540949 | 2 | chr2:65284231 |  | 0.624 | 0.050 |
| rs10188334 | 2 | chr2:653874 |  | 0.828 | 0.050 |
| rs1430780 | 2 | chr2:67878328 |  | 0.320 | 0.027 |
| rs6438247 | 3 | chr3:115084080 |  | 0.131 | 0.044 |
| rs11708067 | 3 | chr3:123065778 |  | 0.757 | 0.078 |
| rs17036160 | 3 | chr3:12329783 | PPARG | 0.882 | 0.103 |
| rs9873519 | 3 | chr3:124921457 |  | 0.532 | 0.038 |
| rs181752889 | 3 | chr3:128579324 |  | 0.002 | 0.282 |
| rs9828772 | 3 | chr3:129333182 |  | 0.897 | 0.052 |
| rs1225052 | 3 | chr3:131644937 |  | 0.375 | 0.027 |
| rs9852406 | 3 | chr3:135625498 |  | 0.253 | 0.038 |
| rs6766859 | 3 | chr3:138055136 |  | 0.372 | 0.033 |
| rs73872717 | 3 | chr3:141134569 |  | 0.954 | 0.086 |
| rs34573045 | 3 | chr3:149196752 |  | 0.433 | 0.031 |
| rs62271373 | 3 | chr3:150066540 |  | 0.060 | 0.069 |
| rs74672008 | 3 | chr3:152451616 |  | 0.964 | 0.080 |
| rs4465929 | 3 | chr3:15741389 |  | 0.402 | 0.030 |
| rs56394279 | 3 | chr3:160171092 |  | 0.471 | 0.031 |
| rs1449348 | 3 | chr3:168225055 |  | 0.860 | 0.045 |
| rs9873618 | 3 | chr3:170733076 |  | 0.710 | 0.058 |
| rs686998 | 3 | chr3:173119768 |  | 0.530 | 0.027 |
| rs2313211 | 3 | chr3:183738626 |  | 0.445 | 0.029 |
| rs10937208 | 3 | chr3:184877626 |  | 0.136 | 0.044 |
| rs9854769 | 3 | chr3:185520948 |  | 0.314 | 0.108 |
| rs3887925 | 3 | chr3:186665645 |  | 0.549 | 0.042 |
| rs6777684 | 3 | chr3:187741842 |  | 0.610 | 0.057 |
| rs7619708 | 3 | chr3:195810187 |  | 0.760 | 0.033 |
| rs35352848 | 3 | chr3:23455582 |  | 0.796 | 0.062 |
| rs10490871 | 3 | chr3:35667761 |  | 0.365 | 0.027 |
| rs9842137 | 3 | chr3:3649850 |  | 0.001 | 0.425 |
| rs11129735 | 3 | chr3:36870230 |  | 0.455 | 0.026 |
| rs62262091 | 3 | chr3:47693664 |  | 0.094 | 0.056 |
| rs4688760 | 3 | chr3:49980596 | MST1R | 0.689 | 0.034 |
| rs2581787 | 3 | chr3:53127677 |  | 0.560 | 0.025 |
| rs76263492 | 3 | chr3:54828827 |  | 0.043 | 0.068 |
| rs2292662 | 3 | chr3:63897215 |  | 0.849 | 0.056 |
| rs4132228 | 3 | chr3:64708114 |  | 0.711 | 0.047 |
| rs844215 | 3 | chr3:71656045 |  | 0.583 | 0.026 |
| rs11922794 | 3 | chr3:72813582 | SHQ1 | 0.245 | 0.030 |
| rs1437055 | 3 | chr3:86831077 |  | 0.617 | 0.027 |
| rs11716527 | 3 | chr3:89986280 |  | 0.105 | 0.049 |
| rs3872707 | 3 | chr3:9514016 | SETD5 | 0.124 | 0.045 |
| rs7695096 | 4 | chr4:103932556 |  | 0.514 | 0.038 |
| rs17035289 | 4 | chr4:106048291 | TET2 | 0.160 | 0.043 |
| rs72501964 | 4 | chr4:1267203 |  | 0.963 | 0.083 |
| rs12509379 | 4 | chr4:129179458 |  | 0.202 | 0.031 |
| rs1724557 | 4 | chr4:137094048 |  | 0.413 | 0.025 |
| rs12505942 | 4 | chr4:140906390 |  | 0.656 | 0.030 |
| rs75686861 | 4 | chr4:145621328 |  | 0.092 | 0.047 |
| rs6819331 | 4 | chr4:153504295 |  | 0.681 | 0.040 |
| rs28819812 | 4 | chr4:157652753 |  | 0.678 | 0.038 |
| rs56337234 | 4 | chr4:1784403 |  | 0.506 | 0.041 |
| rs2011603 | 4 | chr4:18025484 |  | 0.737 | 0.038 |
| rs72695645 | 4 | chr4:185713608 |  | 0.859 | 0.061 |
| rs35901985 | 4 | chr4:186580062 |  | 0.821 | 0.035 |
| rs11940813 | 4 | chr4:20210953 |  | 0.133 | 0.037 |
| rs362307 | 4 | chr4:3241845 |  | 0.074 | 0.050 |
| rs10938398 | 4 | chr4:45186139 |  | 0.434 | 0.043 |
| rs62310934 | 4 | chr4:48880627 |  | 0.618 | 0.030 |
| rs114447556 | 4 | chr4:53207093 |  | 0.082 | 0.058 |
| rs10937721 | 4 | chr4:6306763 |  | 0.591 | 0.084 |
| rs1531583 | 4 | chr4:744972 |  | 0.039 | 0.099 |
| rs2055997 | 4 | chr4:76535086 |  | 0.708 | 0.031 |
| rs11723275 | 4 | chr4:77528821 |  | 0.464 | 0.027 |
| rs10471048 | 4 | chr4:83587562 |  | 0.337 | 0.034 |
| rs987949 | 4 | chr4:85384069 |  | 0.010 | 0.153 |
| rs7660000 | 4 | chr4:89751858 |  | 0.718 | 0.031 |
| rs7656001 | 4 | chr4:91243865 |  | 0.550 | 0.026 |
| rs6821438 | 4 | chr4:95091911 |  | 0.530 | 0.029 |
| rs3755879 | 4 | chr4:96114385 | UNC5C | 0.301 | 0.033 |
| rs75432112 | 5 | chr5:102586407 | PPIP5K2 | 0.050 | 0.134 |
| rs329122 | 5 | chr5:133864599 |  | 0.421 | 0.026 |
| rs112667817 | 5 | chr5:137823156 |  | 0.883 | 0.061 |
| rs146886108 | 5 | chr5:14751305 | ANKH | 0.993 | 0.390 |
| rs890940 | 5 | chr5:158026744 |  | 0.211 | 0.048 |
| rs114136102 | 5 | chr5:36084426 |  | 0.040 | 0.072 |
| rs13155752 | 5 | chr5:44680687 |  | 0.396 | 0.032 |
| rs152839 | 5 | chr5:50145266 |  | 0.584 | 0.026 |
| rs12187734 | 5 | chr5:51763665 |  | 0.515 | 0.029 |
| rs4865796 | 5 | chr5:53272664 |  | 0.692 | 0.047 |
| rs256904 | 5 | chr5:55810305 |  | 0.746 | 0.069 |
| rs4976033 | 5 | chr5:67714246 |  | 0.401 | 0.028 |
| rs253412 | 5 | chr5:74955841 | ANKDD1B | 0.662 | 0.046 |
| rs6878122 | 5 | chr5:76427311 |  | 0.319 | 0.055 |
| rs12519500 | 5 | chr5:78436905 | JMY | 0.650 | 0.038 |
| rs7719891 | 5 | chr5:86577352 |  | 0.247 | 0.040 |
| rs2410767 | 5 | chr5:87705268 |  | 0.787 | 0.033 |
| rs60519666 | 6 | chr6:107427166 |  | 0.676 | 0.035 |
| rs55812705 | 6 | chr6:111738793 |  | 0.755 | 0.031 |
| rs72951506 | 6 | chr6:118011723 |  | 0.844 | 0.042 |
| rs2008027 | 6 | chr6:126052359 |  | 0.516 | 0.027 |
| rs11759026 | 6 | chr6:126792095 |  | 0.228 | 0.065 |
| rs12194820 | 6 | chr6:127401978 |  | 0.755 | 0.046 |
| rs7739842 | 6 | chr6:131954797 |  | 0.189 | 0.033 |
| rs1573090 | 6 | chr6:137302159 |  | 0.538 | 0.045 |
| rs11155073 | 6 | chr6:139837128 |  | 0.409 | 0.030 |
| rs197482 | 6 | chr6:143069315 |  | 0.623 | 0.030 |
| rs9383649 | 6 | chr6:153428102 |  | 0.403 | 0.033 |
| rs727734 | 6 | chr6:15475051 |  | 0.761 | 0.030 |
| rs543159 | 6 | chr6:160776017 |  | 0.527 | 0.032 |
| rs4709746 | 6 | chr6:164133001 |  | 0.865 | 0.058 |
| rs9368112 | 6 | chr6:19718157 |  | 0.529 | 0.026 |
| rs7756992 | 6 | chr6:20679709 |  | 0.265 | 0.122 |
| rs3094682 | 6 | chr6:31264461 | HLA-C | 0.815 | 0.060 |
| rs9275184 | 6 | chr6:32654714 |  | 0.103 | 0.098 |
| rs9296095 | 6 | chr6:33542523 |  | 0.821 | 0.034 |
| rs10305420 | 6 | chr6:39016636 | GLP1R | 0.608 | 0.032 |
| rs34298980 | 6 | chr6:40409243 |  | 0.491 | 0.038 |
| rs4714422 | 6 | chr6:41012405 |  | 0.245 | 0.029 |
| rs11967262 | 6 | chr6:43760327 |  | 0.488 | 0.037 |
| rs3798519 | 6 | chr6:50788778 |  | 0.179 | 0.050 |
| rs1819564 | 6 | chr6:51505337 | PKHD1 | 0.028 | 0.076 |
| rs9379084 | 6 | chr6:7231843 | RREB1 | 0.884 | 0.075 |
| rs534043 | 7 | chr7:100312724 |  | 0.887 | 0.045 |
| rs1968204 | 7 | chr7:102800137 |  | 0.093 | 0.057 |
| rs39328 | 7 | chr7:103444978 |  | 0.424 | 0.028 |
| rs6976111 | 7 | chr7:117495667 |  | 0.302 | 0.032 |
| rs13237518 | 7 | chr7:12269593 | TMEM106B | 0.415 | 0.029 |
| rs1562398 | 7 | chr7:130457931 |  | 0.415 | 0.041 |
| rs1122518 | 7 | chr7:13900325 |  | 0.476 | 0.026 |
| rs62492368 | 7 | chr7:150537635 |  | 0.304 | 0.034 |
| rs2191349 | 7 | chr7:15064309 |  | 0.548 | 0.066 |
| rs6459733 | 7 | chr7:156930550 |  | 0.662 | 0.051 |
| rs38221 | 7 | chr7:15926228 |  | 0.253 | 0.033 |
| rs583769 | 7 | chr7:18331915 |  | 0.246 | 0.031 |
| rs4721089 | 7 | chr7:1872921 |  | 0.782 | 0.034 |
| rs75693095 | 7 | chr7:23440057 |  | 0.022 | 0.112 |
| rs798549 | 7 | chr7:2760750 |  | 0.269 | 0.030 |
| rs1513272 | 7 | chr7:28200097 |  | 0.498 | 0.081 |
| rs917195 | 7 | chr7:30728452 |  | 0.768 | 0.047 |
| rs17439448 | 7 | chr7:40816653 |  | 0.122 | 0.040 |
| rs2908286 | 7 | chr7:44234737 |  | 0.178 | 0.068 |
| rs62450857 | 7 | chr7:4683258 |  | 0.134 | 0.039 |
| rs12539264 | 7 | chr7:48839003 |  | 0.283 | 0.029 |
| rs2876826 | 7 | chr7:50581972 |  | 0.223 | 0.031 |
| rs2103132 | 7 | chr7:69782073 |  | 0.247 | 0.032 |
| rs67755137 | 7 | chr7:74108135 |  | 0.193 | 0.033 |
| rs10240790 | 7 | chr7:89880949 |  | 0.714 | 0.028 |
| rs34340810 | 8 | chr8:105661926 |  | 0.927 | 0.054 |
| rs4734193 | 8 | chr8:110140564 |  | 0.527 | 0.034 |
| rs2409742 | 8 | chr8:11069960 |  | 0.512 | 0.036 |
| rs2737226 | 8 | chr8:116639474 |  | 0.392 | 0.038 |
| rs11558471 | 8 | chr8:118185733 | SLC30A8 | 0.682 | 0.103 |
| rs12056338 | 8 | chr8:12643055 |  | 0.416 | 0.031 |
| rs17772814 | 8 | chr8:128711742 |  | 0.918 | 0.075 |
| rs1561927 | 8 | chr8:129568078 |  | 0.268 | 0.035 |
| rs17294565 | 8 | chr8:14124809 |  | 0.384 | 0.027 |
| rs3757969 | 8 | chr8:145551199 |  | 0.374 | 0.048 |
| rs17091891 | 8 | chr8:19843171 | LPL | 0.880 | 0.042 |
| rs1059592 | 8 | chr8:22477778 |  | 0.354 | 0.027 |
| rs17818197 | 8 | chr8:25872634 |  | 0.224 | 0.035 |
| rs2725371 | 8 | chr8:30854033 |  | 0.303 | 0.037 |
| rs13262861 | 8 | chr8:41508577 |  | 0.825 | 0.102 |
| rs117173251 | 8 | chr8:4186731 |  | 0.033 | 0.077 |
| rs62515938 | 8 | chr8:57483013 |  | 0.261 | 0.029 |
| rs10097617 | 8 | chr8:95961626 |  | 0.469 | 0.037 |
| rs149364428 | 8 | chr8:97737741 |  | 0.008 | 0.224 |
| rs34990153 | 8 | chr8:9996389 |  | 0.556 | 0.038 |
| rs756145 | 9 | chr9:1039939 |  | 0.306 | 0.029 |
| rs7858727 | 9 | chr9:111936128 | EPB41L4B | 0.205 | 0.034 |
| rs1431819 | 9 | chr9:116943357 |  | 0.696 | 0.029 |
| rs7026688 | 9 | chr9:125975397 |  | 0.863 | 0.044 |
| rs1752169 | 9 | chr9:126586563 |  | 0.250 | 0.032 |
| rs495203 | 9 | chr9:136145240 |  | 0.318 | 0.049 |
| rs448918 | 9 | chr9:136885979 |  | 0.265 | 0.033 |
| rs28429551 | 9 | chr9:139243334 | GPSM1 | 0.755 | 0.073 |
| rs10963942 | 9 | chr9:19080352 |  | 0.390 | 0.037 |
| rs7867635 | 9 | chr9:20241069 |  | 0.411 | 0.037 |
| rs10811661 | 9 | chr9:22134094 |  | 0.826 | 0.138 |
| rs11793831 | 9 | chr9:23362311 |  | 0.415 | 0.027 |
| rs1412234 | 9 | chr9:28410683 |  | 0.327 | 0.044 |
| rs12001437 | 9 | chr9:34074476 |  | 0.368 | 0.034 |
| rs10974438 | 9 | chr9:4291928 |  | 0.351 | 0.047 |
| rs1929883 | 9 | chr9:81344701 |  | 0.584 | 0.039 |
| rs17791513 | 9 | chr9:81905590 |  | 0.938 | 0.069 |
| rs2796441 | 9 | chr9:84308948 |  | 0.581 | 0.059 |
| rs555784 | 9 | chr9:85318704 |  | 0.617 | 0.030 |
| rs10821311 | 9 | chr9:96943059 |  | 0.318 | 0.036 |
| rs7046845 | 9 | chr9:97804641 |  | 0.911 | 0.047 |
| rs2862954 | 10 | chr10:101912064 | ERLIN1 | 0.502 | 0.029 |
| rs2250301 | 10 | chr10:104548393 | WBP1L | 0.751 | 0.032 |
| rs10787287 | 10 | chr10:112647195 |  | 0.767 | 0.036 |
| rs7903146 | 10 | chr10:114758349 |  | 0.287 | 0.280 |
| rs11257655 | 10 | chr10:12307894 |  | 0.207 | 0.091 |
| rs2280141 | 10 | chr10:124193181 | PLEKHA1 | 0.528 | 0.045 |
| rs878017 | 10 | chr10:13566204 |  | 0.527 | 0.035 |
| rs36051838 | 10 | chr10:34018730 |  | 0.086 | 0.044 |
| rs12263348 | 10 | chr10:65305252 |  | 0.348 | 0.028 |
| rs10998304 | 10 | chr10:70342775 | TET1 | 0.451 | 0.031 |
| rs177045 | 10 | chr10:71321279 | NEUROG3 | 0.315 | 0.039 |
| rs827237 | 10 | chr10:72648336 |  | 0.199 | 0.037 |
| rs12773019 | 10 | chr10:73835274 |  | 0.027 | 0.089 |
| rs2675662 | 10 | chr10:75599127 |  | 0.563 | 0.027 |
| rs7099048 | 10 | chr10:77647107 |  | 0.515 | 0.028 |
| rs703981 | 10 | chr10:80942855 |  | 0.543 | 0.061 |
| rs11201999 | 10 | chr10:88124501 |  | 0.541 | 0.026 |
| rs10788575 | 10 | chr10:89768584 |  | 0.164 | 0.035 |
| rs7071943 | 10 | chr10:93956552 |  | 0.659 | 0.044 |
| rs1111875 | 10 | chr10:94462882 |  | 0.591 | 0.092 |
| rs10882891 | 10 | chr10:99059645 |  | 0.403 | 0.031 |
| rs10750397 | 11 | chr11:128234144 |  | 0.278 | 0.048 |
| rs117316450 | 11 | chr11:14518419 |  | 0.021 | 0.131 |
| rs5219 | 11 | chr11:17409572 | KCNJ11 | 0.357 | 0.069 |
| rs4929965 | 11 | chr11:2197286 |  | 0.379 | 0.062 |
| rs2237895 | 11 | chr11:2857194 |  | 0.415 | 0.073 |
| rs62618693 | 11 | chr11:32956492 | QSER1 | 0.955 | 0.085 |
| rs11038672 | 11 | chr11:45846498 | CTD-2210P24.4 | 0.475 | 0.029 |
| rs116861182 | 11 | chr11:55588216 |  | 0.057 | 0.064 |
| rs174541 | 11 | chr11:61565908 |  | 0.640 | 0.029 |
| rs35169799 | 11 | chr11:64031241 | PLCB3 | 0.063 | 0.050 |
| rs1783541 | 11 | chr11:65294799 |  | 0.217 | 0.048 |
| rs144245804 | 11 | chr11:69453044 |  | 0.974 | 0.130 |
| rs11602873 | 11 | chr11:72460762 |  | 0.843 | 0.098 |
| rs480840 | 11 | chr11:74625997 |  | 0.429 | 0.025 |
| rs10899283 | 11 | chr11:76505202 |  | 0.778 | 0.031 |
| rs10769936 | 11 | chr11:8654528 | TRIM66 | 0.725 | 0.035 |
| rs10830963 | 11 | chr11:92708710 |  | 0.274 | 0.089 |
| rs2403221 | 11 | chr11:9852475 | SBF2 | 0.674 | 0.032 |
| rs3764002 | 12 | chr12:108618630 | WSCD2 | 0.738 | 0.040 |
| rs34965774 | 12 | chr12:118412373 |  | 0.130 | 0.052 |
| rs56348580 | 12 | chr12:121432117 |  | 0.692 | 0.058 |
| rs12820906 | 12 | chr12:123493123 |  | 0.753 | 0.043 |
| rs12823740 | 12 | chr12:124458002 | DNAH10OS | 0.666 | 0.041 |
| rs11830243 | 12 | chr12:132544694 |  | 0.108 | 0.044 |
| rs11614914 | 12 | chr12:133070294 |  | 0.327 | 0.039 |
| rs12305809 | 12 | chr12:133777466 | ANHX | 0.606 | 0.033 |
| rs10841868 | 12 | chr12:21781246 |  | 0.740 | 0.032 |
| rs11048458 | 12 | chr12:26465585 |  | 0.243 | 0.046 |
| rs10771372 | 12 | chr12:27962260 | MANSC4 | 0.803 | 0.072 |
| rs10771813 | 12 | chr12:31367856 |  | 0.554 | 0.026 |
| rs10844518 | 12 | chr12:33410780 |  | 0.279 | 0.033 |
| rs2733289 | 12 | chr12:41838235 |  | 0.478 | 0.030 |
| rs11181613 | 12 | chr12:43046449 |  | 0.849 | 0.043 |
| rs117233107 | 12 | chr12:4328521 |  | 0.985 | 0.325 |
| rs2732480 | 12 | chr12:48736303 | ZNF641 | 0.572 | 0.034 |
| rs7132908 | 12 | chr12:50263148 |  | 0.384 | 0.033 |
| rs1872635 | 12 | chr12:54541750 |  | 0.688 | 0.028 |
| rs2583921 | 12 | chr12:66170481 |  | 0.089 | 0.095 |
| rs67013744 | 12 | chr12:6681786 |  | 0.166 | 0.035 |
| rs1705263 | 12 | chr12:71523043 | TSPAN8 | 0.560 | 0.040 |
| rs11108094 | 12 | chr12:95928113 |  | 0.069 | 0.060 |
| rs113036477 | 12 | chr12:97848227 |  | 0.943 | 0.072 |
| rs9555581 | 13 | chr13:109944192 |  | 0.613 | 0.030 |
| rs314879 | 13 | chr13:23309382 |  | 0.212 | 0.039 |
| rs34584161 | 13 | chr13:26776999 |  | 0.764 | 0.052 |
| rs9319382 | 13 | chr13:28245127 |  | 0.685 | 0.028 |
| rs3742305 | 13 | chr13:31036642 |  | 0.732 | 0.030 |
| rs576674 | 13 | chr13:33554302 |  | 0.167 | 0.061 |
| rs4397977 | 13 | chr13:41688401 |  | 0.342 | 0.029 |
| rs9316500 | 13 | chr13:51094114 |  | 0.704 | 0.047 |
| rs9563574 | 13 | chr13:58656599 |  | 0.827 | 0.041 |
| rs11616380 | 13 | chr13:80705315 |  | 0.712 | 0.079 |
| rs1475655 | 13 | chr13:91963080 |  | 0.739 | 0.044 |
| rs73347525 | 14 | chr14:101255172 |  | 0.818 | 0.049 |
| rs12890750 | 14 | chr14:103860309 |  | 0.651 | 0.028 |
| rs8005994 | 14 | chr14:29744532 |  | 0.645 | 0.027 |
| rs17522122 | 14 | chr14:33302882 |  | 0.470 | 0.034 |
| rs799661 | 14 | chr14:35390146 |  | 0.891 | 0.043 |
| rs8018512 | 14 | chr14:38818723 |  | 0.749 | 0.037 |
| rs2933211 | 14 | chr14:47313541 |  | 0.493 | 0.027 |
| rs10137475 | 14 | chr14:58797953 |  | 0.422 | 0.026 |
| rs4899280 | 14 | chr14:69526307 |  | 0.332 | 0.028 |
| rs8008540 | 14 | chr14:74948180 |  | 0.563 | 0.030 |
| rs2056857 | 14 | chr14:77300863 |  | 0.582 | 0.026 |
| rs10145154 | 14 | chr14:79939525 |  | 0.222 | 0.055 |
| rs8010382 | 14 | chr14:91963722 |  | 0.414 | 0.032 |
| rs11073147 | 15 | chr15:36392562 |  | 0.551 | 0.025 |
| rs12912777 | 15 | chr15:38852386 |  | 0.125 | 0.059 |
| rs11639470 | 15 | chr15:39639171 |  | 0.535 | 0.027 |
| rs484943 | 15 | chr15:40398754 |  | 0.338 | 0.033 |
| rs2289739 | 15 | chr15:41801512 |  | 0.345 | 0.050 |
| rs74804697 | 15 | chr15:52588722 |  | 0.955 | 0.084 |
| rs75332279 | 15 | chr15:53099306 |  | 0.100 | 0.056 |
| rs2435907 | 15 | chr15:57333416 |  | 0.582 | 0.029 |
| rs8033609 | 15 | chr15:60938816 |  | 0.542 | 0.027 |
| rs7163757 | 15 | chr15:62391608 |  | 0.571 | 0.041 |
| rs34143602 | 15 | chr15:63940058 |  | 0.410 | 0.035 |
| rs1874832 | 15 | chr15:67260238 |  | 0.154 | 0.038 |
| rs4776970 | 15 | chr15:68080886 |  | 0.642 | 0.029 |
| rs12917449 | 15 | chr15:74331659 | PML | 0.196 | 0.036 |
| rs6495182 | 15 | chr15:75814388 |  | 0.749 | 0.041 |
| rs12910361 | 15 | chr15:77782335 |  | 0.712 | 0.072 |
| rs36111056 | 15 | chr15:83461873 | WHAMM | 0.789 | 0.034 |
| rs8031576 | 15 | chr15:90380214 |  | 0.280 | 0.057 |
| rs2290203 | 15 | chr15:91512067 |  | 0.198 | 0.056 |
| rs9927842 | 16 | chr16:15153717 |  | 0.155 | 0.038 |
| rs62034975 | 16 | chr16:20392415 |  | 0.302 | 0.031 |
| rs7188071 | 16 | chr16:28917644 |  | 0.353 | 0.029 |
| rs8054556 | 16 | chr16:29958216 |  | 0.467 | 0.036 |
| rs55857387 | 16 | chr16:300388 |  | 0.801 | 0.052 |
| rs12933120 | 16 | chr16:3634746 |  | 0.143 | 0.042 |
| rs1421085 | 16 | chr16:53800954 |  | 0.403 | 0.118 |
| rs2032912 | 16 | chr16:69568303 |  | 0.590 | 0.042 |
| rs72802342 | 16 | chr16:75234872 |  | 0.923 | 0.115 |
| rs2925979 | 16 | chr16:81534790 |  | 0.299 | 0.045 |
| rs11646052 | 16 | chr16:85716463 |  | 0.394 | 0.026 |
| rs11117364 | 16 | chr16:88132199 |  | 0.676 | 0.029 |
| rs12920022 | 16 | chr16:89564055 |  | 0.161 | 0.039 |
| rs4984980 | 16 | chr16:968292 |  | 0.182 | 0.035 |
| rs2297508 | 17 | chr17:17715317 |  | 0.351 | 0.033 |
| rs7220340 | 17 | chr17:27566326 |  | 0.460 | 0.027 |
| rs12602834 | 17 | chr17:29637308 |  | 0.385 | 0.029 |
| rs4796224 | 17 | chr17:34842521 |  | 0.472 | 0.025 |
| rs11657964 | 17 | chr17:36100767 |  | 0.397 | 0.059 |
| rs11078916 | 17 | chr17:37746307 | STARD3 | 0.276 | 0.037 |
| rs8071043 | 17 | chr17:3988451 |  | 0.330 | 0.054 |
| rs684214 | 17 | chr17:40696915 | MLX | 0.280 | 0.042 |
| rs9900074 | 17 | chr17:46124326 |  | 0.928 | 0.055 |
| rs35895680 | 17 | chr17:47060322 |  | 0.673 | 0.056 |
| rs11870735 | 17 | chr17:481604 |  | 0.180 | 0.034 |
| rs2243102 | 17 | chr17:4839149 |  | 0.406 | 0.026 |
| rs1451506 | 17 | chr17:57407019 |  | 0.115 | 0.042 |
| rs4325 | 17 | chr17:61563200 |  | 0.532 | 0.035 |
| rs11655898 | 17 | chr17:62201374 |  | 0.070 | 0.059 |
| rs2080090 | 17 | chr17:65828371 | C17orf58 | 0.189 | 0.053 |
| rs61736066 | 17 | chr17:70645032 | SLC39A11 | 0.913 | 0.051 |
| rs858519 | 17 | chr17:7531965 |  | 0.443 | 0.026 |
| rs1656794 | 17 | chr17:75386909 |  | 0.725 | 0.031 |
| rs62075585 | 17 | chr17:76762039 |  | 0.473 | 0.030 |
| rs9912236 | 17 | chr17:77895311 |  | 0.754 | 0.031 |
| rs7219033 | 17 | chr17:9787958 |  | 0.329 | 0.029 |
| rs11662800 | 18 | chr18:13271367 |  | 0.415 | 0.028 |
| rs303760 | 18 | chr18:21083738 |  | 0.345 | 0.034 |
| rs346240 | 18 | chr18:40063830 |  | 0.204 | 0.031 |
| rs72926932 | 18 | chr18:53050646 |  | 0.080 | 0.075 |
| rs17684074 | 18 | chr18:54675384 |  | 0.748 | 0.031 |
| rs1517037 | 18 | chr18:56878274 | GRP | 0.812 | 0.038 |
| rs663640 | 18 | chr18:57846077 |  | 0.219 | 0.050 |
| rs12454712 | 18 | chr18:60845884 |  | 0.623 | 0.041 |
| rs7240767 | 18 | chr18:7070642 |  | 0.387 | 0.037 |
| rs2658746 | 18 | chr18:74582340 |  | 0.378 | 0.030 |
| rs35004890 | 19 | chr19:1224286 |  | 0.231 | 0.036 |
| rs11666603 | 19 | chr19:12496934 |  | 0.764 | 0.033 |
| rs3111316 | 19 | chr19:13038415 |  | 0.587 | 0.044 |
| rs10404726 | 19 | chr19:18834514 |  | 0.533 | 0.028 |
| rs58542926 | 19 | chr19:19379549 | TM6SF2 | 0.074 | 0.089 |
| rs4805681 | 19 | chr19:31835516 |  | 0.605 | 0.027 |
| rs10406327 | 19 | chr19:33890838 |  | 0.521 | 0.037 |
| rs429358 | 19 | chr19:45411941 | APOE | 0.846 | 0.073 |
| rs10407429 | 19 | chr19:46157237 |  | 0.574 | 0.054 |
| rs11667244 | 19 | chr19:47580185 |  | 0.715 | 0.035 |
| rs12977104 | 19 | chr19:4949921 |  | 0.204 | 0.041 |
| rs17175860 | 19 | chr19:7235146 |  | 0.194 | 0.045 |
| rs2115107 | 19 | chr19:7968168 | LRRC8E | 0.382 | 0.038 |
| rs2268078 | 20 | chr20:32596704 |  | 0.641 | 0.039 |
| rs17265513 | 20 | chr20:39832628 | ZHX3 | 0.199 | 0.033 |
| rs419842 | 20 | chr20:42310811 |  | 0.842 | 0.041 |
| rs12625671 | 20 | chr20:42994812 |  | 0.107 | 0.065 |
| rs6066138 | 20 | chr20:45594711 |  | 0.718 | 0.045 |
| rs867489 | 20 | chr20:48833957 |  | 0.536 | 0.031 |
| rs2426439 | 20 | chr20:50999627 |  | 0.633 | 0.037 |
| rs2252221 | 20 | chr20:51621922 |  | 0.526 | 0.025 |
| rs911300 | 20 | chr20:57387262 |  | 0.543 | 0.035 |
| rs1815591 | 20 | chr20:61277014 |  | 0.389 | 0.034 |
| rs4809369 | 20 | chr20:62470785 |  | 0.548 | 0.034 |
| rs75756987 | 21 | chr21:47767295 | PCNT | 0.896 | 0.044 |
| rs75401573 | 22 | chr22:29805444 |  | 0.922 | 0.051 |
| rs5753043 | 22 | chr22:30588041 | MTMR3 | 0.908 | 0.061 |
| rs117001013 | 22 | chr22:32348841 |  | 0.914 | 0.047 |
| rs133015 | 22 | chr22:38572526 |  | 0.560 | 0.029 |
| rs5751061 | 22 | chr22:41593873 |  | 0.629 | 0.026 |
| rs3747207 | 22 | chr22:44324855 |  | 0.215 | 0.047 |
| rs5771069 | 22 | chr22:50435480 |  | 0.497 | 0.033 |

| **Table S16. Single-nucleotide polymorphisms associated with circulating n-3 PUFAs*** | | | | | | | | |  |
| --- | --- | --- | --- | --- | --- | --- | --- | --- | --- |
| **Type of FA** | **FA** | **SNP** | **Nearby gene** | **Chr** | **EA** | **NEA** | **Beta** | **SE** |  |
| n-3 PUFA | ALA | rs174547 | FADS1 | 11 | C | T | 0.02 | 0.001 |  |
|  |  | rs1535 | FADS2 | 11 | G | A | 0.05 | 0.005 |  |
|  |  | rs174555 | FADS1 | 11 | C | T | 0.06 | 0.004 |  |
|  |  | rs964184 | ZPR1 | 11 | G | C | 0.04 | 0.006 |  |
|  |  | rs968567 | FADS2 | 11 | T | C | 0.05 | 0.007 |  |
|  |  | rs7200543 | NTAN1 | 16 | A | G | 0.04 | 0.005 |  |
|  | EPA | rs3798713 | ELOVL2 | 6 | C | G | 0.04 | 0.005 |  |
|  |  | rs174538 | FADS1/C11orf10 | 11 | G | A | 0.08 | 0.005 |  |
|  |  | rs174556 | FADS1 | 11 | C | T | 0.04 | 0.004 |  |
|  |  | rs174535 | MYRF | 11 | T | C | 0.04 | 0.005 |  |
|  |  | rs174536 | MYRF | 11 | A | C | 0.13 | 0.008 |  |
|  |  | rs174546 | FADS1 | 11 | C | T | 0.1 | 0.005 |  |
|  |  | rs2295602 | ELOVL2 | 6 | T | C | 0.04 | 0.005 |  |
|  | DPA | rs780094 | GCKR | 2 | T | C | 0.02 | 0.003 |  |
|  |  | rs3734398 | ELOVL2 | 6 | C | T | 0.04 | 0.003 |  |
|  |  | rs174547 | FADS1 | 11 | T | C | 0.08 | 0.003 |  |
|  |  | rs9393915 | ELOVL2 | 6 | T | C | 0.04 | 0.004 |  |
|  |  | rs174538 | FADS1 | 11 | G | A | 0.03 | 0.004 |  |
|  | DHA | rs2236212 | ELOVL2 | 6 | G | C | 0.11 | 0.014 |  |
|  |  | rs102275 | TMEM258 | 11 | T | C | 0.07 | 0.005 |  |
|  |  | rs174535 | MYRF | 11 | T | C | 0.06 | 0.007 |  |
|  |  | rs174546 | FADS1 | 11 | C | T | 0.06 | 0.007 |  |
|  |  | rs174547 | FADS1 | 11 | T | C | 0.05 | 0.005 |  |
|  |  | rs1077989 | TMEM229B | 14 | A | C | 0.04 | 0.005 |  |
|  |  | rs3794991 | GATAD2A | 19 | C | T | 0.08 | 0.012 |  |
|  |  | rs2295602 | ELOVL2 | 6 | T | C | 0.03 | 0.004 |  |
|  |  | rs4711171 | ELOVL2 | 6 | C | T | 0.04 | 0.006 |  |
| *****SNPs associated with circulating n-3 PUFAs were obtained from GWASs: (1) Lemaitre, R. N. et al. Genetic loci associated with plasma phospholipid n-3 fatty acids: a meta-analysis of genome-wide association studies from the CHARGE Consortium. *PLoS Genet.* **7**, e1002193-e1002193 (2011). (2) Draisma, H. H. M. et al. Genome-wide association study identifies novel genetic variants contributing to variation in blood metabolite levels. *Nat Commun.* **6**, 7208 (2015). (3) Shin, S. Y. et al. An atlas of genetic influences on human blood metabolites. *Nat Genet*. **46**, 543–550 (2014). | | | | | | | | | |

| **Table S17. Diet composition of each animal group** | | |  |  |  |  |  |  |
| --- | --- | --- | --- | --- | --- | --- | --- | --- |
| Diet | AIN93G | |  | AIN93G+DHA | |  | AIN93G+EPA | |
| Group | *db/db* or WT | |  | *db/db*+DHA | |  | *db/db*+EPA | |
|  | gm% | *kcal%* |  | gm% | *kcal%* |  | gm% | *kcal%* |
| Protein | 20 | 20 |  | 20 | 20 |  | 20 | 20 |
| Carbohydrate | 64 | 64 |  | 64 | 64 |  | 64 | 64 |
| Fat | 7 | 16 |  | 7 | 16 |  | 7 | 16 |
| Total |  | 100 |  |  | 100 |  |  | 100 |
| kcal/gm | 4.00 |  |  | 4.00 |  |  | 4.00 |  |
| Content | gm | kcal |  | gm | kcal |  | gm | kcal |
| Casein | 200 | 800 |  | 200 | 800 |  | 200 | 800 |
| L-cystine | 3 | 12 |  | 3 | 12 |  | 3 | 12 |
| Corn starch | 397.486 | 1590 |  | 397.486 | 1590 |  | 397.486 | 1590 |
| Maltodextrin | 132 | 528 |  | 132 | 528 |  | 132 | 528 |
| Sucrose | 100 | 400 |  | 100 | 400 |  | 100 | 400 |
| Cellulose BW200 | 50 | 0 |  | 50 | 0 |  | 50 | 0 |
| Soybean oil | 70 | 630 |  | 60 | 540 |  | 60 | 540 |
| EPA (>99%, unesterified) | 0 | 0 |  | 0 | 0 |  | 10 | 90 |
| DHA (>99%, unesterified) | 0 | 0 |  | 10 | 90 |  | 0 | 0 |
| TBHQ | 0.014 | 0 |  | 0.014 | 0 |  | 0.014 | 0 |
| Mineral mix S10022G | 35 | 0 |  | 35 | 0 |  | 35 | 0 |
| Vitamin Mix V10037 | 10 | 40 |  | 10 | 40 |  | 10 | 40 |
| Choline Bitartrate | 2.5 | 0 |  | 2.5 | 0 |  | 2.5 | 0 |
| Total | 1000 | 4000 |  | 1000 | 4000 |  | 1000 | 4000 |

| **Table S18. qPCR primers** | |  |
| --- | --- | --- |
| **Gene** | **Forward Primer (5'→3')** | **Reverse Primer (5'→3')** |
| *Rab4* | TGGGGCACTCCTTGTCTATG | GCGCATTGTAGGTTTCTCGG |
| *Rab5* | GCTAATCGAGGAGCAACAAGAC | CCAGGCTTGATTTGCCAACAG |
| *Rab8a* | GACAGGTGTCCAAGGAACGG | GATCCCATAGTCGAGTGCCAG |
| *Rab13* | CTACCAGTGTTGGCTCTTCCC | GGATCTTGAAATCAATTCCGATGG |
| *Rab14* | GGTGCGCTCATGGTGTATGA | TGGTGAGATTCCTTGCGTCT |
| *VAMP2* | TCATCTTGGGAGTGATCTGCG | GGGCAGACTCCTCAGGGATT |
| *SNAP23* | CCGCCGGAGTATCCGTATTT | TGTTCCCCTTGCTCATCCAG |
| *Syntaxin4* | AGCAGTTGGAGCGCAGTATC | TGTTCTTCTCGATGCGGTTGA |
| *GAPDH* | AGGTCGGTGTGAACGGATTTG | TGTAGACCATGTAGTTGAGGTCA |
|  |  |  |

| **Table S19. Antibodies used in this study** | |  |  |
| --- | --- | --- | --- |
| **Antibody** | **Manufacturer** | **Catalog #** | **Dilution** |
| SNAP23 | Abcam | Cat# ab4114 | 1:1000 |
| Syntaxin4 | Abcam | Cat# ab184545 | 1:1000 |
| VAMP2 | Abcam | Cat# ab3347 | 1:1000 |
| Rab8a | Abcam | Cat# ab188574 | 1:1000 |
| Glycogen synthase 1 | Abcam | Cat# ab40810 | 1:1000 |
| AKT (pan) | Cell Signalling Technology | Cat# 4691 | 1:1000 |
| Phospho-Akt (Ser473) | Cell Signalling Technology | Cat# 4060 | 1:1000 |
| GLUT1 | Affinity Biosciences | Cat# AF0173 | 1:1000 |
| GLUT4 | Affinity Biosciences | Cat# AF5386 | 1:1000 |
| PDK4 | Affinity Biosciences | Cat# DF7169 | 1:1000 |
| HA-Tag Antibody | Affinity Biosciences | Cat# T0008 | 1:100 |
| Na, K-ATPase α1 | SANTA CRUZ | Cat# sc-21712 | 1:200 |
| GAPDH | EarthOx Life Sciences | Cat#E021060 | 1:1000 |
| Goat Anti-Rabbit IgG | EarthOx Life Sciences | Cat#E031620 | 1:10000 |
| Goat Anti-Mouse IgG | EarthOx Life Sciences | Cat#E031610 | 1:10000 |
| Dylight594, Goat Anti-Mouse IgG | EarthOx Life Sciences | Cat#E032410 | 1:500 |
